# Exploring the 5-Substituted 2-Aminobenzothiazole-Based DNA Gyrase B Inhibitors Active against ESKAPE Pathogens

**DOI:** 10.1021/acsomega.3c01930

**Published:** 2023-06-28

**Authors:** Maša Sterle, Martina Durcik, Clare E. M. Stevenson, Sara R. Henderson, Petra Eva Szili, Marton Czikkely, David M. Lawson, Anthony Maxwell, Dominique Cahard, Danijel Kikelj, Nace Zidar, Csaba Pal, Lucija Peterlin Mašič, Janez Ilaš, Tihomir Tomašič, Andrej Emanuel Cotman, Anamarija Zega

**Affiliations:** † Faculty of Pharmacy, University of Ljubljana, Aškerčeva Cesta 7, Ljubljana 1000, Slovenia; ‡ Department of Biochemistry and Metabolism, John Innes Centre, Norwich Research Park, Norwich NR4 7UH, U.K.; § Institute of Microbiology and Infection, College of Medical and Dental Sciences, University of Birmingham, Birmingham B15 2TT, U.K.; ∥ Synthetic and Systems Biology Unit, Biological Research Centre, Institute of Biochemistry, Szeged H-6726, Hungary; ⊥ CNRS UMR 6014 COBRA, Normandie Université, Mont Saint Aignan 76821, France

## Abstract

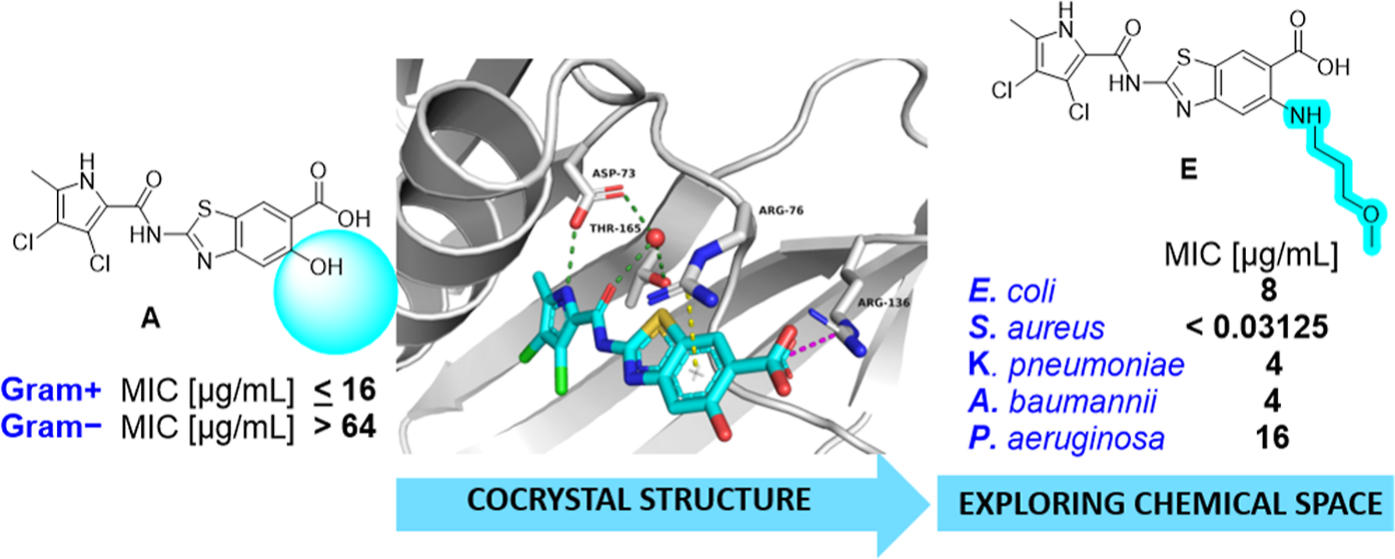

We present a new series of 2-aminobenzothiazole-based DNA gyrase B inhibitors with promising activity against ESKAPE bacterial pathogens. Based on the binding information extracted from the cocrystal structure of DNA gyrase B inhibitor **A**, in complex with *Escherichia coli* GyrB24, we expanded the chemical space of the benzothiazole-based series to the C5 position of the benzothiazole ring. In particular, compound **E** showed low nanomolar inhibition of DNA gyrase (IC_50_ < 10 nM) and broad-spectrum antibacterial activity against pathogens belonging to the ESKAPE group, with the minimum inhibitory concentration < 0.03 μg/mL for most Gram-positive strains and 4–16 μg/mL against Gram-negative *E. coli*, *Acinetobacter baumannii*, *Pseudomonas aeruginosa,* and *Klebsiella pneumoniae*. To understand the binding mode of the synthesized inhibitors, a combination of docking calculations, molecular dynamics (MD) simulations, and MD-derived structure-based pharmacophore modeling was performed. The computational analysis has revealed that the substitution at position C5 can be used to modify the physicochemical properties and antibacterial spectrum and enhance the inhibitory potency of the compounds. Additionally, a discussion of challenges associated with the synthesis of 5-substituted 2-aminobenzothiazoles is presented.

## 1 Introduction

Overuse and misuse of antibacterials in human medicine, animal health, and agriculture have led to the spread of antibiotic-resistant bacteria. This is a great concern and one of the most complex global health challenges. Multidrug-resistant and highly virulent pathogens from the ESKAPE group, which consists of Gram-negative *Acinetobacter baumannii*, *Pseudomonas aeruginosa*, and *Enterobacteriaceae* and Gram-positive *Staphylococcus aureus* and *Enterococcus faecium*, are all on the WHO global critical and high-priority list (2017) to promote research, discovery, and development of new antibiotics. To effectively combat antibacterial resistance, new antibacterial drugs are urgently needed.^1−4^

**Figure 1:**
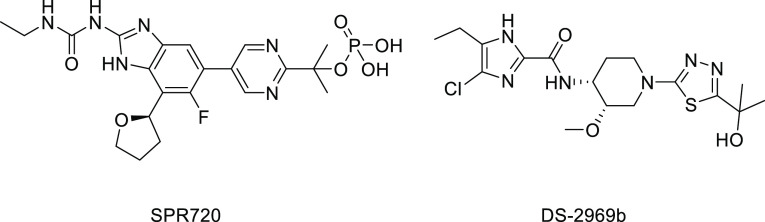
Structures of fobrepodacin (SPR720) and DS-2969b.

DNA gyrase and topoisomerase IV (Topo IV), both are type II topoisomerases, are attractive and well-established targets for antibacterial drug discovery with potential for dual targeting, which prolongs the onset of resistance. These enzymes catalyze reactions involving transient breaks of both strands of DNA: relaxation of supercoiled DNA and introduction of negative supercoils into the molecule. Both of these reactions are ATP dependent. DNA gyrase is a heterotetrameric protein consisting of two GyrA and two GyrB subunits (A_2_B_2_), whereas Topo IV is composed of two ParC and two ParE subunits (C_2_E_2_), homologous to GyrA and GyrB, respectively. Fluoroquinolones, by far the most successful antibacterials targeting DNA gyrase, interact with the GyrA and ParC subunits. The GyrB and ParE subunits contain the ATP-binding site and are the site of action for coumarin antibiotics.^5,6^ The aminocoumarin novobiocin was the first and thus far the only ATP-competitive GyrB inhibitor in clinical use but was withdrawn due to safety issues and lack of efficacy. Despite the wide array of new structurally diverse GyrB and ParE inhibitors discovered, none are currently in clinical use.^7,8^ However, DNA gyrase remains a promising target, as fobrepodacin (SPR720) and DS-2969b are currently in clinical trials for the treatment of nontuberculous mycobacterial and *Clostridium difficile* infections, respectively (Figure 1).^9,10^

During our ongoing research on DNA gyrase and Topo IV inhibitors, we discovered ATP-competitive benzothiazole scaffold-based inhibitors that display potent antibacterial activity against ESKAPE pathogens.^11−20^ As a part of our hit-to-lead development stage, we expanded the chemical space of the benzothiazole-based series to the synthetically challenging C5 position of the benzothiazole ring. In this paper, we present new inhibitors with a broad spectrum of activity against Gram-positive and Gram-negative bacteria and investigate their possible binding modes by a combination of docking calculations, molecular dynamics (MD) simulations, and MD-derived structure-based pharmacophore modeling. Additionally, an in-depth description of the synthesis of C5-substituted benzothiazoles is presented.

## 2 Results and Discussion

### 2.1 Design

**Figure 2:**
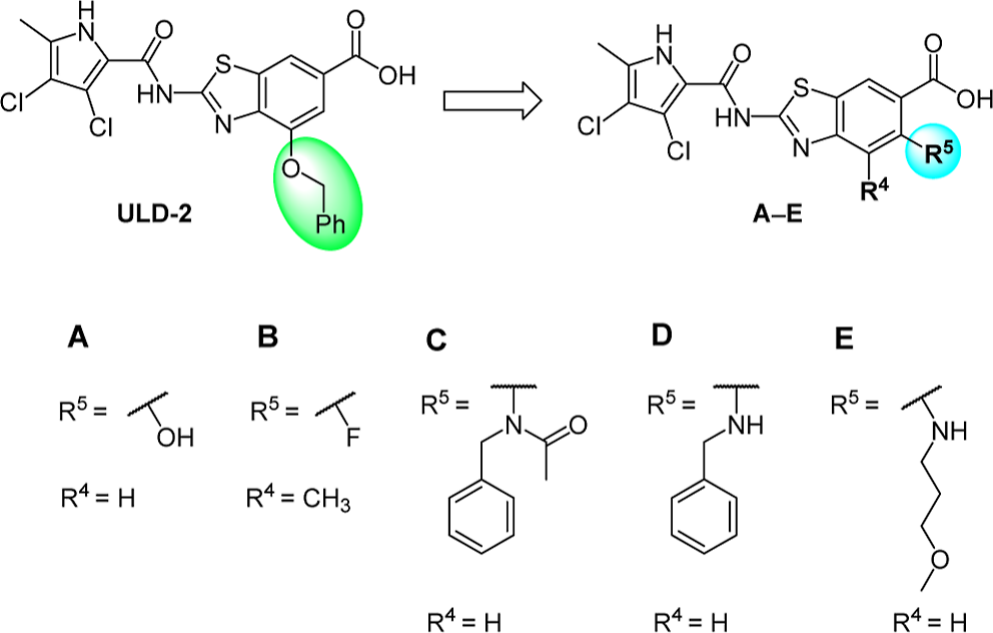
New series of compounds (A–E) with substitution at the C5 position of the benzothiazole scaffold.

Our recent series of topoisomerase inhibitors, which exhibit favorable low nanomolar enzyme inhibition and potent antibacterial activity, focused on the study of the substituent at position C4 of the 2-aminobenzothiazole core, which plays an important role in providing additional interactions with the ATP-binding site (Figure 2).

However, the C5 position on the benzothiazole ring remained underexplored. The only compound with a substituent on the C5 position previously prepared and tested was inhibitor **A**(18) bearing an −OH group at C5. The compound displayed good activity against DNA gyrase (IC_50_ < 10 nM) and Topo IV (IC_50_ = 95 ± 4 nM) from *E. coli* and relatively good antibacterial activity against Gram-positive bacteria but is inactive against Gram-negative bacterial strains [minimum inhibitory concentration (MIC) > 64 μg/mL] (Table 1).

**Table 1:**
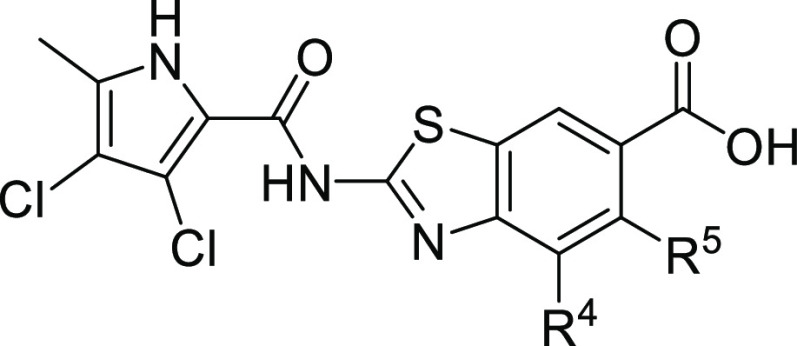

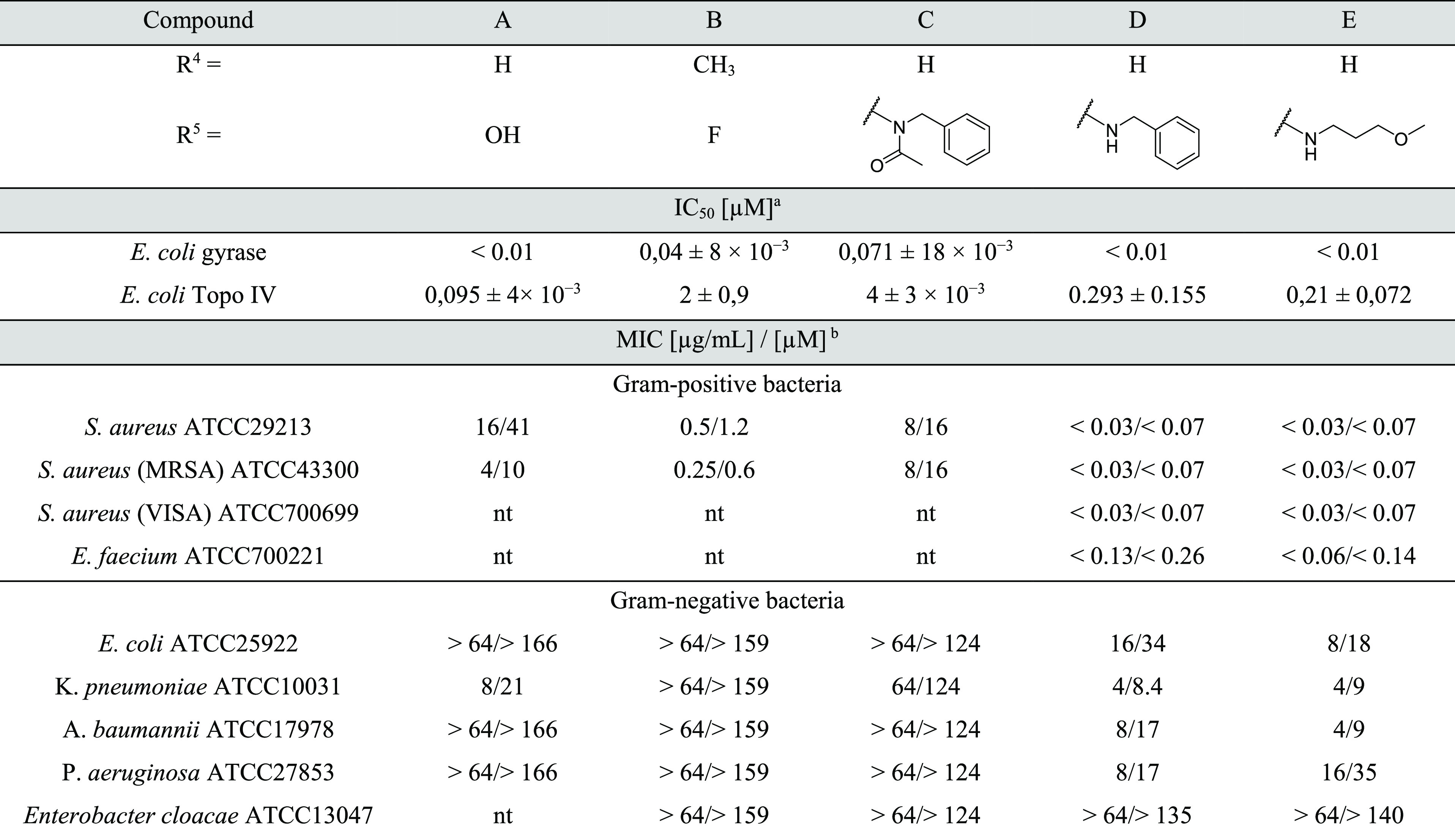
MICs and IC_50_ Values for Inhibitors **A–E** against Gram-Positive and Gram-Negative Bacterial Strains

a IC_50_, concentration (mean ± SD of three independent experiments) that inhibits enzyme activity by 50%.

b MIC, minimum inhibitory concentration. MIC measurements were performed according to the Clinical and Laboratory Standards Institute guidelines, with three independent measurements. nt, not tested.

**Figure 3:**
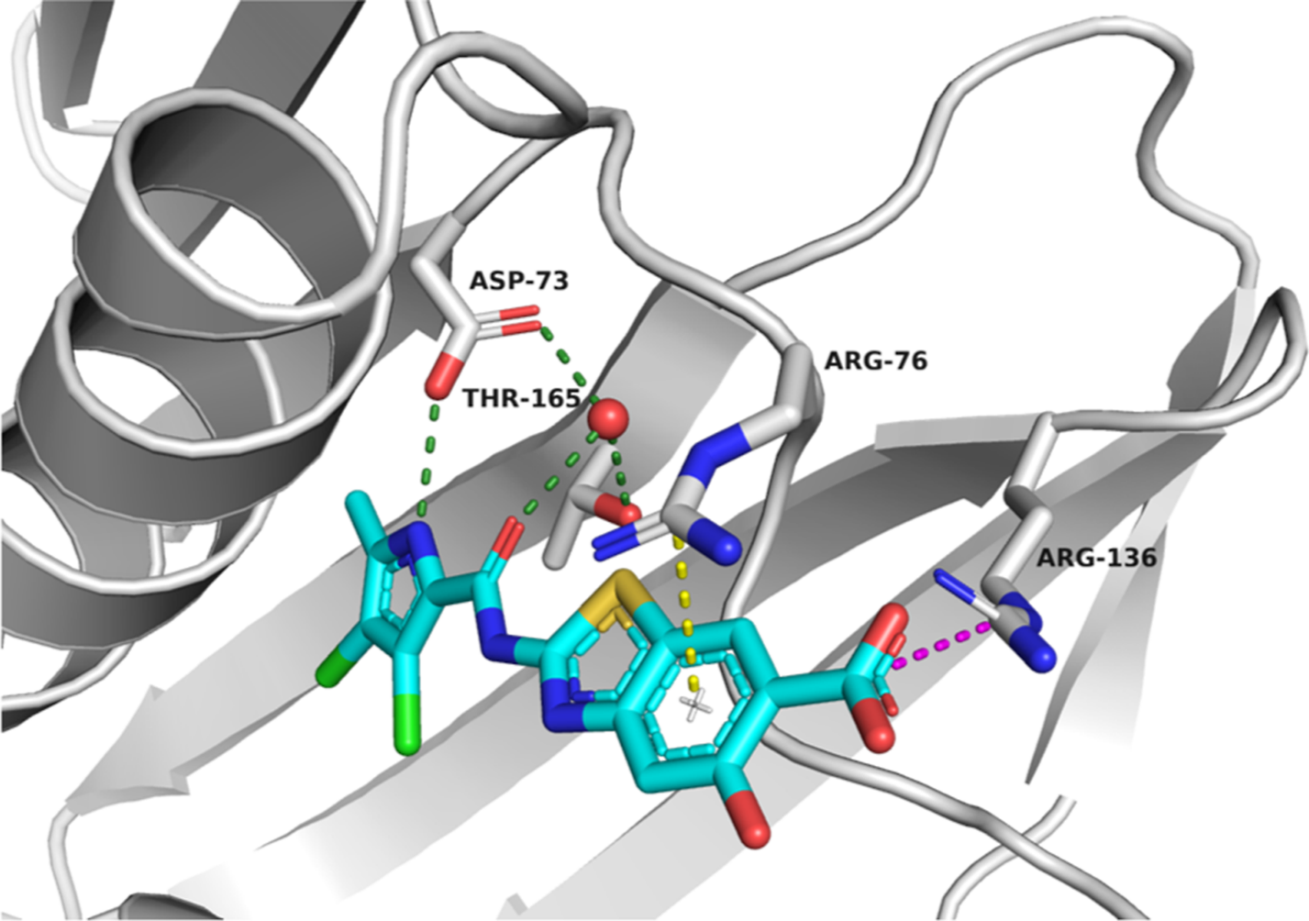
Cocrystal structure of compound **A** (in cyan sticks) in the ATP-binding site of *E. coli* DNA gyrase B (in gray cartoon; PDB ID 7P2N). The ligand and the amino acid residues that interact with it are shown as stick models and colored according to atom type (blue, N; red, O; light green, Cl; and brown, S). The water molecule is presented as a red sphere, hydrogen bonds are indicated by green dotted lines, the salt bridge is indicated by a magenta dotted line, and the cation−π interaction is indicated by a yellow dotted line.

To investigate the potential of position C5 of the benzothiazole for optimization, we first solved a crystal structure of **A** in a complex with a 24 kDa fragment of *E. coli* GyrB (GyrB24) at a resolution of 1.16 Å (PDB code: 7P2N) (Figure 3). As expected, the inhibitor is bound in the ATP-binding site of GyrB24. The benzothiazole ring is involved in a cation-π-stacking interaction with the Arg76 side chain. The aromatic carboxylate group at C6 of the benzothiazole of inhibitor **A** and the Arg136 side chain form a salt bridge. The carboxamide oxygen interacts with a conserved water molecule and the hydroxyl group in the side chain of Thr165. The 3,4-dichloro-5-methylpyrrole moiety is involved in hydrophobic interactions in the lipophilic pocket with residues Val167, Val120, Ile78, Val71, Ala47, and Val43, while the NH group of pyrrole forms a hydrogen bond with the side chain of Asp73. The hydroxy group at C5 of the benzothiazole scaffold is oriented toward the solvent-exposed area. This allows introduction of larger substituents to C5, which could influence the pharmacokinetic properties of benzothiazole-based compounds and possibly broaden the antibacterial activity.

Based on the data obtained from the crystal structure and favorable on-target activity of inhibitor **A**, we decided to further explore the chemical space by the introduction of a fluorine atom and amine substituents at the C5 position (Figure 2). To probe potential interactions of new substituents with the enzyme, docking calculations and MD simulations were performed using the cocrystal structure of *E. coli* GyrB containing the flexible loop Leu98-Gly117, which is absent in the crystal structure of **A** and GyrB (PDB ID 7P2N).

Our objective was to synthesize novel compounds that would retain potent inhibitory activity against the target enzymes, improve antibacterial activity against Gram-positive strains, and at the same time broaden the spectrum of activity to Gram-negative bacterial strains.

### 2.2 Chemistry

**Scheme 1:**
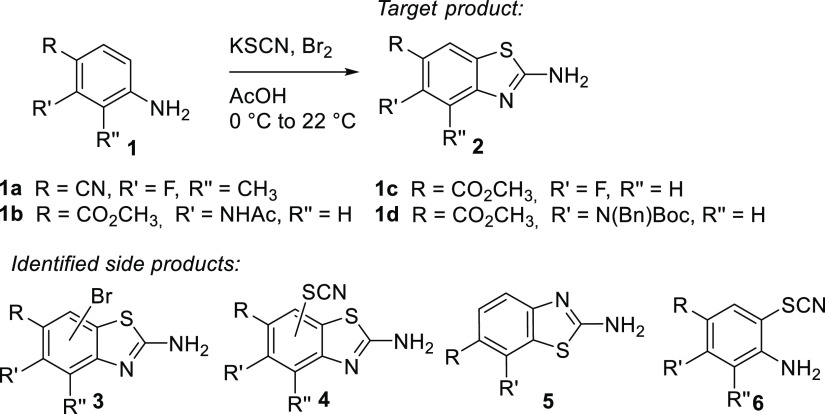
Target Products **2** and Identified Side Products: Products with Additional Br or SCN on Benzothiazole Scaffolds **3** and **4**, Respectively, Incorrectly Cycled Product **5**, and Acyclic Side Product **6**

For the synthesis of inhibitors, one of the crucial synthetic steps was the formation of 5-substituted methyl 2-aminobenzo[*d*]thiazole-6-carboxylates **2** from the corresponding 2-substituted methyl 4-aminobenzoates **1** (Scheme 1). We first attempted the synthesis of the 2-aminobenzothiazoles **2** using our general synthetic protocol involving potassium thiocyanate (4 equiv) and bromine (2 equiv) in glacial acetic acid.^21^ Starting from 4-amino-2-fluoro-3-methylbenzonitrile (**1a**), methyl 2-acetamido-4-aminobenzoate (**1b**), or methyl 4-amino-2-fluorobenzoate (**1c**), the formation of the target benzothiazoles **2** was very low yielding due to the formation of side products with a similar solubility profile and chromatographic retention factor, which made isolation difficult. The identified side products **3–6** are presented in Scheme 1. For detailed side product analysis, see Schemes S1–S4 and Figures S1–S4.

To potentially reduce the number of side products **3** and **4**, the excess of KSCN and Br_2_ was reduced (from 4 and 2 equiv to 3 and 1.5 equiv, respectively), and some modifications in the reaction protocol were implemented. In particular, instead of mixing KSCN and the starting aniline **1**, followed by adding Br_2_ dropwise at 0 °C, the thiocyanogen reagent, (SCN)_2_, was pre-formed from KSCN and Br_2_ in acetic acid at 22 °C and added dropwise to the solution of aniline **1** in a small volume of acetic acid. This solved the issue of brominated side products **3**.

**Scheme 2:**
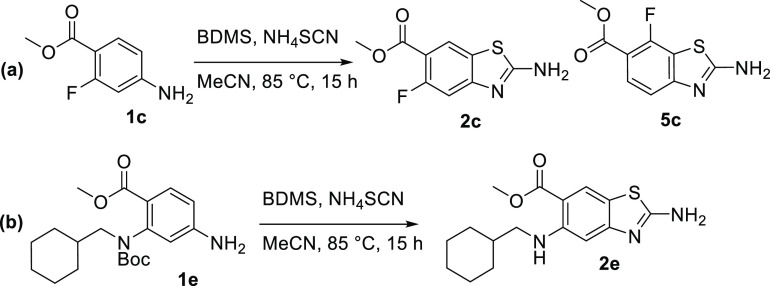
(a) Synthesis of the Target Product **2c** and Its Regioisomer **5c** and (b) Synthesis of Compound **2e** where the Formation of Its Regioisomer Is Prevented

Instead of highly toxic and corrosive molecular Br_2_ that is hazardous and difficult to handle, alternative sources of Br_2_ for in situ formation of thiocyanogen have been described, such as solid benzyltrimethylammonium dichloroiodate^22^ and bromodimethylsulfonium bromide (BDMS).^23^ In our hands, using the first one resulted in no conversion, while using a combination of BDMS and ammonium thiocyanate for the conversion of aniline **1c**, the formation of side products **3c**, **4c**, and **6c** was prevented. Only the regioisomers **2c** and **5c** were formed, and we managed to isolate the analytically pure desired product **2c** after chromatographic purification, although the isolated yield was less than 5% (Scheme 2a). The same protocol was used to prepare the (cyclohexylmethyl)amino analogue **2e** from the Boc-protected **1e** (Scheme 2b). In this case, the formation of the unwanted regioisomer **5e** was prevented by the bulky *N*-(cyclohexylmethyl)Boc-amino substituent. During the reaction or workup, the Boc protection was cleaved, and the diamine **2e** was isolated in 14% yield, which was not suitable for further elaboration to the target topoisomerase inhibitors.

**Scheme 3:**
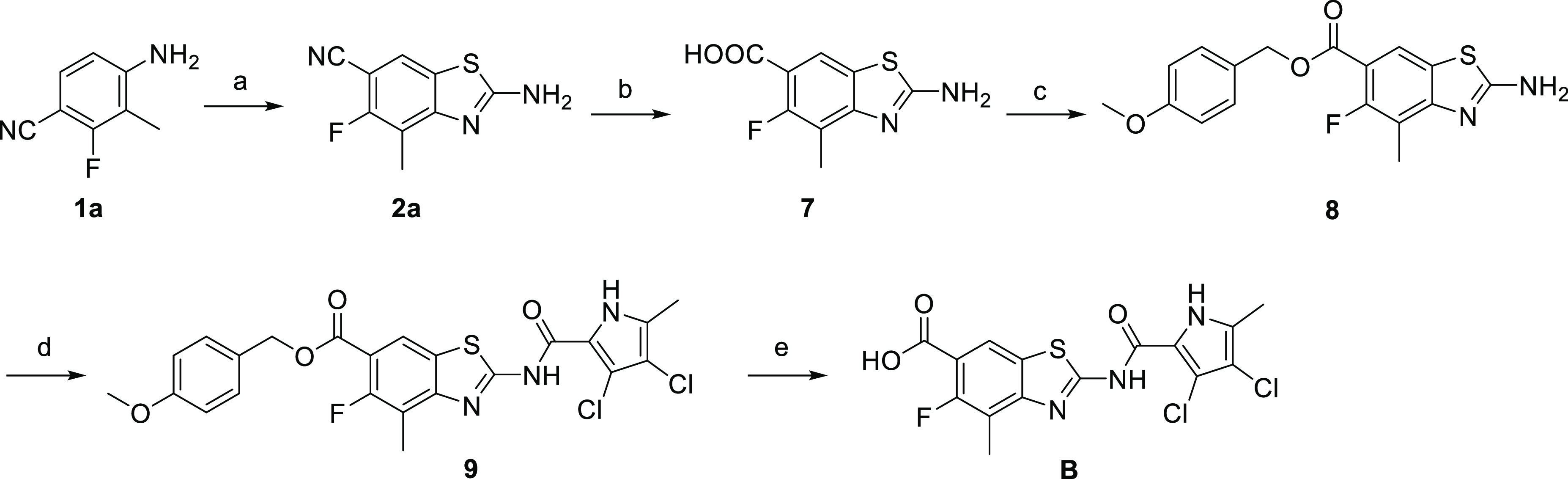
Synthesis of Compound **B** Reagents and conditions: (a) KSCN, Br_2_, acetic acid, 22 °C, overnight; (b) H_2_O/H_2_SO_4_/acetic acid = 1:1:1, 130 °C, overnight; (c) *p*-methoxybenzyl chloride, K_2_CO_3_, dry DMF, 40 °C, overnight; (d) 2-trichloroacetyl-3,4-dichloro-5-methyl-1*H*-pyrrole, Na_2_CO_3_, DMF, 60 °C, overnight; and (e) 1 M HCl/acetic acid, then 4 M HCl in 1,4-dioxane, 22–50 °C, 30 h.

After these preliminary synthetic attempts, it became clear that bulky substituents at the meta position of anilines **1** can prevent the formation of the regioisomeric products **5** and that the pre-forming thiocyanogen or the use of alternative oxidizing agents can prevent the formation of bromo and thiocyano side products **3** and **4**. These synthetic tools were eventually adequate to prepare the designed inhibitors of DNA gyrase and Topo IV **B–E**. To evaluate the influence of the fluorine atom at C5 on biological activity, we prepared compound **B** with a methyl group at C4 (Scheme 3). Because of its small size, the methyl group should not affect the on-target or antibacterial activity of the final compound, but it does improve synthetic feasibility by blocking the formation of the corresponding regioisomer **5a**. First, **1a** was dissolved in glacial acetic acid and cooled to 0 °C. (SCN)_2_, prepared ex situ by mixing KSCN (4 equiv) and Br_2_ (2 equiv) in glacial acetic acid, was added dropwise to the solution of the starting compound and stirred overnight at 22 °C to get **2a**. Hydrolysis of the cyano group of **2a** gave **7**. The newly formed carboxylic group was protected as *p*-methoxybenzyl ester. The resulting amine **8** was coupled with 2-trichloroacetyl-3,4-dichloro-5-methyl-1*H*-pyrrole in DMF in the presence of Na_2_CO_3_ at 60 °C to obtain **9**. In the last step, the carboxylic group was deprotected by acidolysis to yield the final product **B**.

**Scheme 4:**
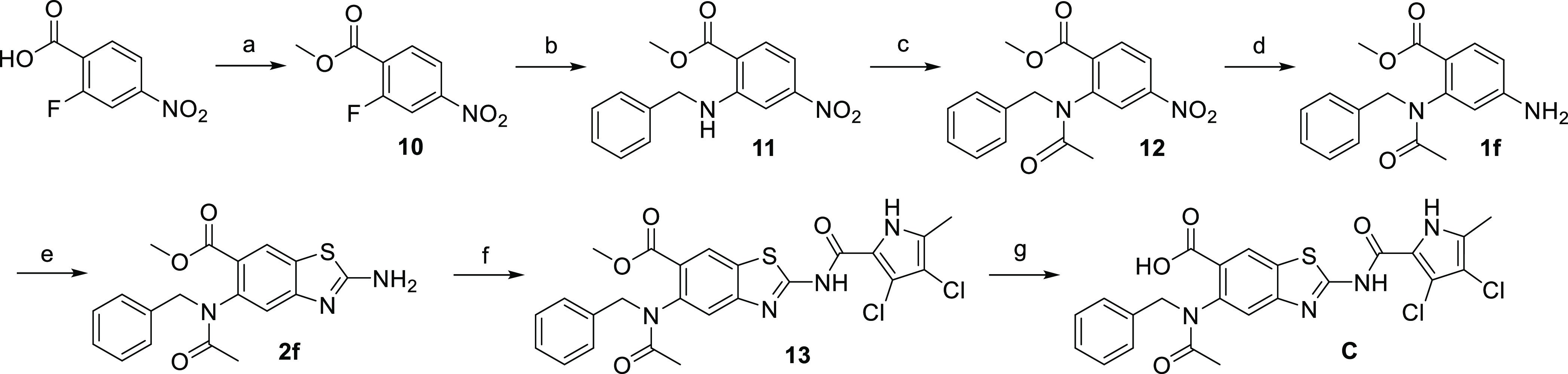
Synthesis of Compound C Reagents and conditions: (a) H_2_SO_4_, MeOH, 65 °C, overnight; (b) benzylamine, K_2_CO_3_, CH_3_CN, 60 °C, overnight; (c) acetyl chloride, 4-DMAP, DIPEA, dry DCM, 22–35 °C, overnight; (d) Fe^0^, acetic acid, 22 °C, 2 h; (e) KSCN, Br_2_, acetic acid, 22 °C, overnight; (f) 2-trichloroacetyl-3,4-dichloro-5-methyl-1*H*-pyrrole, Na_2_CO_3_, dry DMF, 22–60 °C, overnight; and (g) 2 M NaOH, MeOH, 60 °C, 3 days.

Compound **C** was synthesized according to Scheme 4. 2-Fluoro-4-nitrobenzoic acid was first converted to methyl ester **10** using H_2_SO_4_ in methanol. The fluorine atom was displaced by phenylmethanamine to give the secondary amine **11** in a nucleophilic aromatic substitution reaction. The amine was acylated with acetyl chloride in the presence of 4-dimethylaminopyridine (4-DMAP) and *N*,*N*-diisopropylethylamine (DIPEA) to obtain **12**. The nitro group was then reduced using iron in glacial acetic acid to give **1f**. The above described cyclization procedure was applied to obtain the desired product **2f**, which was coupled with 2-trichloroacetyl-3,4-dichloro-5-methyl-1*H*-pyrrole to yield the pyrrolamide **13**, which was then subjected to alkaline hydrolysis of methyl ester to give the final compound **C**.

**Scheme 5:**
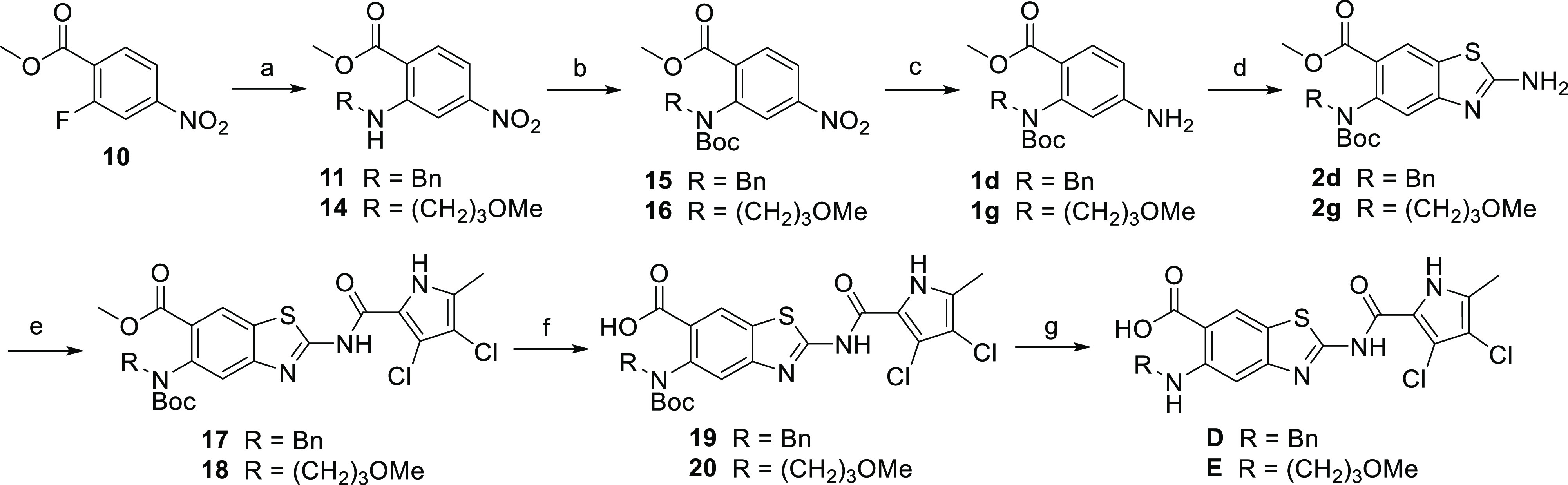
Synthesis of Compounds **D** and **E** Reagents and conditions: (a) R–NH_2_, K_2_CO_3_, CH_3_CN, 60 °C, overnight; (b) Boc_2_O, 4-DMAP, THF, 40 °C, overnight; (c) Fe^0^, acetic acid, 22 °C, 2 h; (d) KSCN, Br_2_, acetic acid, 22 °C, overnight; (e) 3,4-dichloro-5-methylpyrrole-2-carbonyl chloride, toluene, 130 °C, overnight; (f) 2 M NaOH, MeOH, 60 °C, 3 days; and (g) 4 M HCl in 1,4-dioxane, 22 °C, 2 h.

Compounds **D** and **E** were synthesized according to Scheme 5. 2-Fluoro-4-nitrobenzoic acid was first converted to the methyl ester **10** using H_2_SO_4_ in methanol. The fluorine atom was displaced by the corresponding amine (phenylmethanamine or 3-methoxypropan-1-amine) to give a secondary amine **11** or **14** in a nucleophilic aromatic substitution reaction. The amino group of **11** or **14** was Boc-protected to obtain **15** or **16**. The nitro group was then reduced to amino using iron in glacial acetic acid to give **1d** or **1g**. The cyclization procedure described above was applied to **1d** and **1g** to obtain the desired product **2d** and **2g**, respectively. These were coupled with 3,4-dichloro-5-methylpyrrole-2-carbonyl chloride to yield pyrrolamides **17** and **18**, which were subjected to alkaline hydrolysis of methyl ester to give **19** and **20**. Boc deprotection then yielded the final compounds **D** and **E**.

### 2.3 In Vitro Enzyme Inhibition and Antibacterial Activity

Compounds **B**, **C**, **D**, and **E** were evaluated against DNA gyrase from *E. coli* supercoiling and against Topo IV in relaxation assay. The results are presented in Table 1 and Figure S5 as concentrations of compounds that inhibit the enzyme activity by 50% (IC_50_ values). All new inhibitors were found to have nanomolar inhibition of DNA gyrase with IC_50_ values < 71 nM. Replacement of the hydroxyl group (**A**; IC_50_ < 10 nM) at C5 of the benzothiazole ring with a fluorine atom (**B**; IC_50_ = 40 nM), and the *N*-benzylacetamido group (**C**; IC_50_ = 71 nM), resulted in weaker inhibition of gyrase, whereas compounds with secondary amine substituents, a benzylamino group in **D** and a 3-methoxypropylamino group in **E**, retained low nanomolar inhibition (IC_50_ < 10 nM). Larger differences were observed for activity against Topo IV, where all substitutions implemented resulted in weaker activity, with **B** and **C** being inactive (having IC_50_ values of 2000 and 4000 nM, respectively). Compounds **D** and **E** showed IC_50_ values of 293 and 210 nM, respectively, and can be considered to act as dual-targeting DNA gyrase/Topo IV inhibitors. Compounds **B**, **C**, **D**, and **E** were tested for antibacterial activity against Gram-positive and Gram-negative bacteria, including ESKAPE pathogens.

The three compounds, **B**, **D**, and **E**, have, in comparison to inhibitor **A**, demonstrated improved MIC values against Gram-positives *S. aureus*, MRSA, VISA, and *E. faecium*, with excellent results for compounds **D** and **E**, showing MIC < 0.03 μg/mL for most strains. Moreover, whereas **B** and **C**, similarly to compound **A**, showed no activity against Gram-negative strains (MIC values > 64 μg/mL); for inhibitors **D** and **E**, potent activities in the range of 4–16 μg/mL were detected against *E. coli*, *A. baumannii*, *P. aeruginosa,* and *K. pneumoniae*. In spite of having similar on-target activity on DNA gyrase and being even less potent inhibitors of Topo IV than compound **A**, inhibitors **D** and **E** bearing secondary amine substituents on position C5 show better, broad-spectrum antibacterial activity. To evaluate selectivity against a related human target, compounds **D** and **E** were tested for their inhibitory activity on human topoisomerase IIα. Both compounds showed good selectivity, with an enzyme residual activity of 100% for **D** and 55% for **E** at 100 μM.

### 2.4 Docking Calculations, MD Simulations, Structure-Based Pharmacophore Modeling

**Figure 4:**
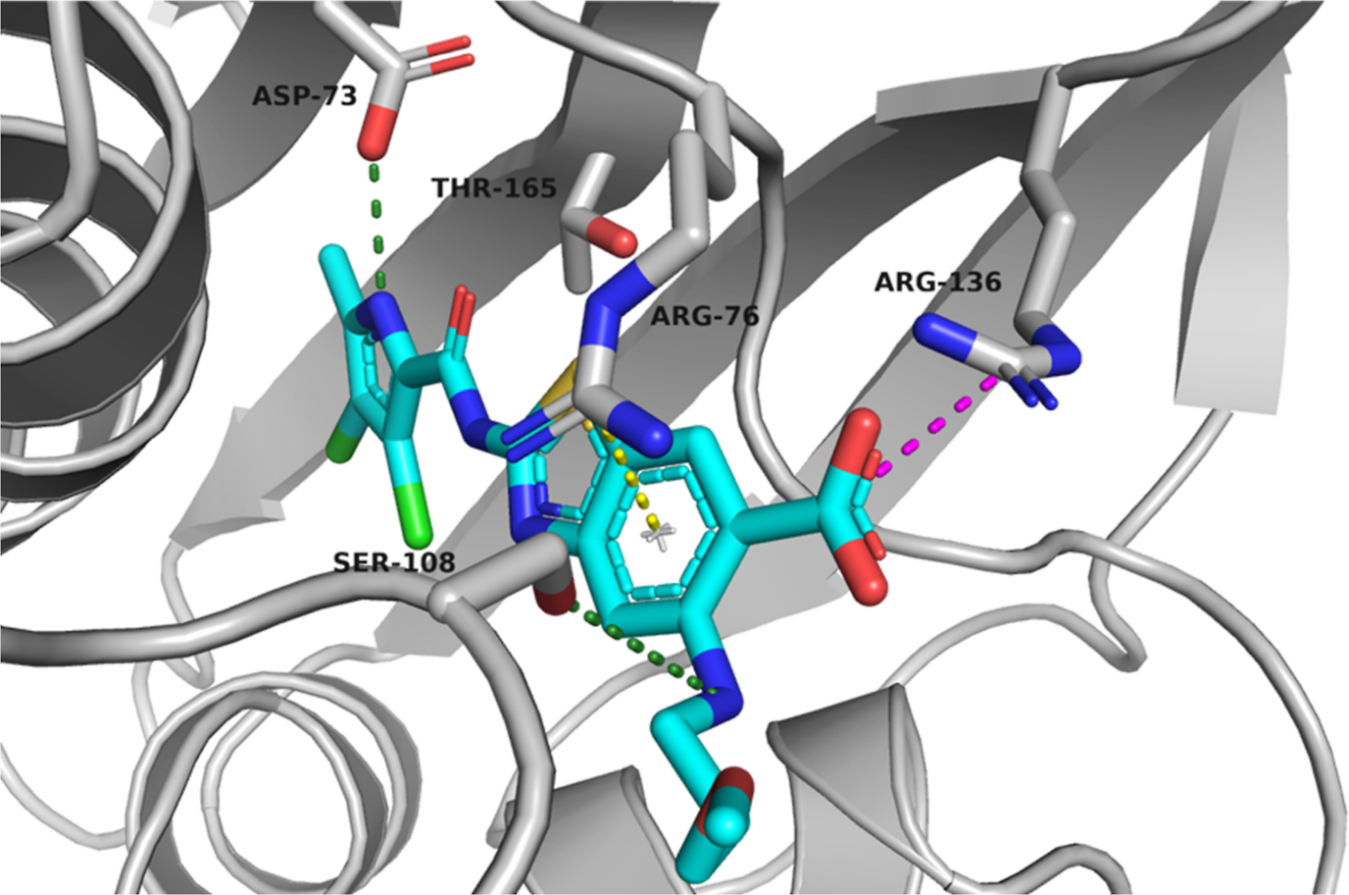
Representative binding mode from the MD simulation trajectory of compound **E** in the ATP-binding site of *E. coli* DNA gyrase B (in gray cartoon; PDB ID 4WUB). The ligand and amino acid residues that interact with ligands are shown as stick models, according to atom types (blue, N; red, O; light green, Cl; and brown, S). Hydrogen bonds are indicated by green dotted lines, the salt bridge is indicated by a magenta dotted line, and the cation−π interaction is indicated by a yellow dotted line.

The binding modes of inhibitors **D** and **E** in the ATP-binding site of *E. coli* DNA gyrase were studied by a combination of docking calculations, MD simulations, and MD-derived structure-based pharmacophore modeling. For molecular modeling, we used the cocrystal structure of *E. coli* GyrB in complex with phosphoaminophosphonic acid-adenylate ester (PDB ID 4WUB),^24^ in which the flexible loop Leu98-Gly117, absent in the complex with **A** (PDB ID 7P2N), is present and allows investigation of the potential interactions between the substituent at C5 and the enzyme. Docking calculations showed that the binding mode of the core 2-(3,4-dichloro-5-methyl-1*H*-pyrrole-2-carboxamido)benzo[*d*]thiazole-6-carboxylic acid is identical to that of **A** (Figure 3). No additional interactions with GyrB and the benzylamino group of **D** were predicted by docking. However, in the case of **E**, a hydrogen bond was suggested between the oxygen atom of the 3-methoxypropylamino group and the Ser108 side chain. To investigate the possible interactions between the benzylamino group of **D** or the 3-methoxypropylamino group of **E** with the flexible loop Leu98-Gly117 of *E. coli* GyrB, we performed 100 ns MD simulations starting from the docking complexes. Analysis of the interactions in the MD trajectory using structure-based pharmacophore modeling in LigandScout revealed that the 3-methoxypropylamino group of **E** rarely interacted with GyrB, as hydrogen bonding with Ser108 was present for only 1% of the simulation time (Figure 4).

**Figure 5:**
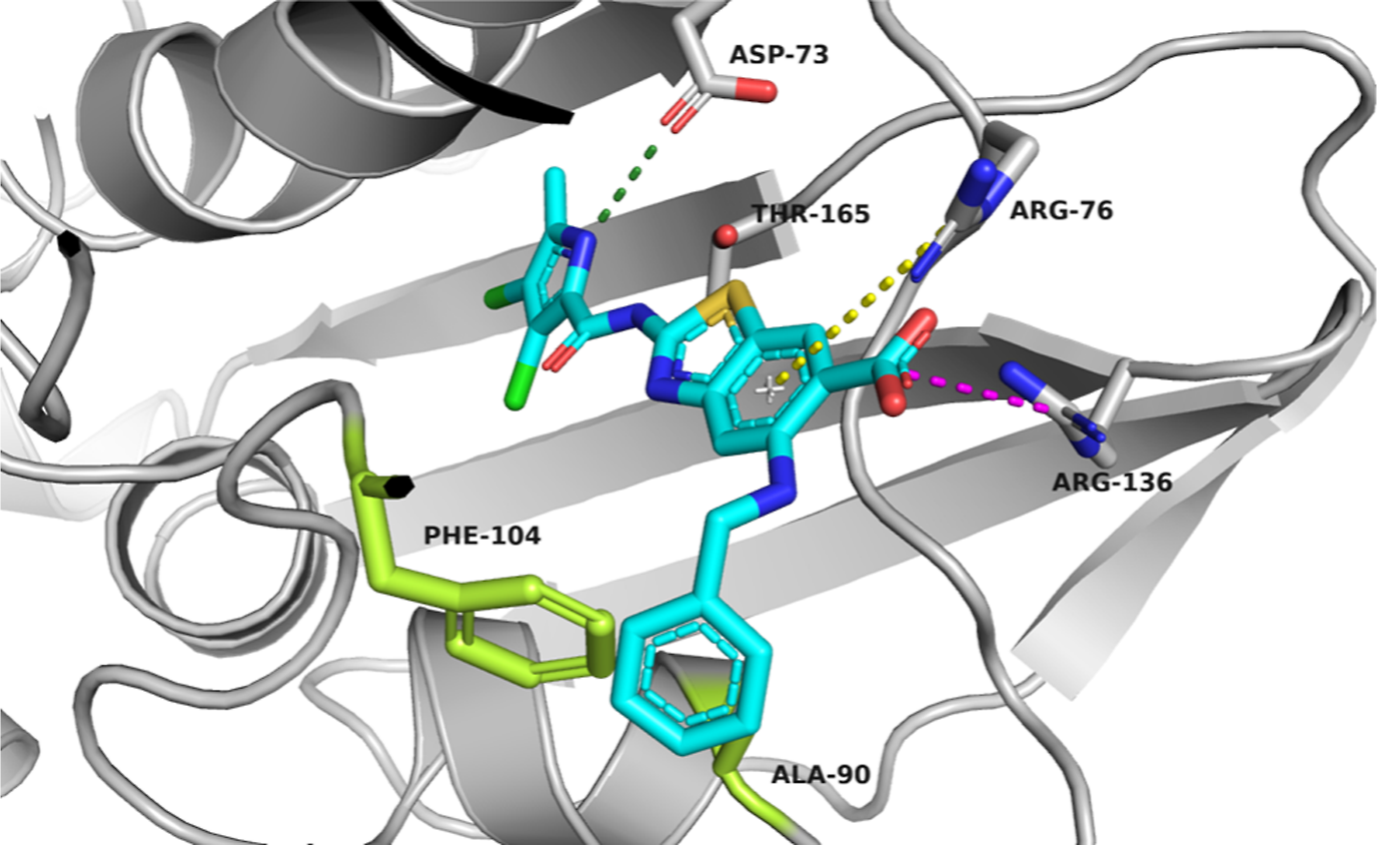
Representative binding mode from the MD simulation trajectory of compound **D** in the ATP-binding site of *E. coli* DNA gyrase B (in gray cartoon; PDB ID 4WUB). The ligand and the amino acid residues that interact with it are shown as sticks models and colored according to atom type (blue, N; red, O; light green, Cl; and brown, S). Hydrogen bonds are indicated by green dotted lines, the salt bridge is indicated by a magenta dotted line, and the cation−π interaction is indicated by a yellow dotted line.

In contrast, the benzylamino group of **D** formed additional interactions with the amino acid residues in the flexible loop, namely, a π-stacking interaction with the Phe104 side chain and hydrophobic interactions with Ala90, Val93, and Phe104 side chains (Figure 5). From these analyses, we corroborated that the substitution at position C5 can also be used to boost the inhibitory potency of the compounds and not only affect the physicochemical properties.

## 3 Conclusions

Growing the 2-aminobenzothiazole-cored topoisomerase inhibitors at C5 was deemed promising based on the cocrystal structure of inhibitor **A** in complex with *E. coli* GyrB24 and on the corresponding in silico design. The synthesis of 5-substituted 2-aminobenzothiazoles has proven to be challenging due to the low reaction yields and an unfavorable impurity profile, but we have developed synthetic tools for the preparation of the model dual-targeting 2-aminobenzothiazole-based DNA gyrase and Topo IV inhibitors with substitution at C5. In particular, compounds **D** and **E** showed low nanomolar inhibition of gyrase and nanomolar inhibition of Topo IV from *E. coli*. Both compounds showed broad-spectrum antibacterial activity against pathogens belonging to the ESKAPE group.

## 4 Experimental Section

### 4.1 Synthetic Procedures and Analytical Data

The synthetic procedures and analytical data, including NMR, MS, and HPLC data, are available in the Supporting Information.

### 4.2 Determination of Inhibitory Activities on *E. coli* DNA Gyrase, Topoisomerase IV, and Human Topoisomerase IIα

Commercially available assay kits (Inspiralis Limited, Norwich, UK) were used for the determination of IC_50_ values for test compounds for inhibition of DNA gyrase supercoiling and Topo IV and TopoIIα relaxation. Assays were performed according to previously reported procedures.^25^ For details on enzyme assays, see the Supporting Information.

### 4.3 Determination of Antibacterial Activity

Antimicrobial assays (MICs) were performed by a standard serial broth microdilution method. For details on antimicrobial assays, see the Supporting Information.

### 4.4 X-Ray Crystallography

The X-ray structure of compound **A** in complex with *E. coli* GyrB24 was obtained at a resolution of 1.16 Å (PDB code: 7P2N). For details on protein expression and purification, as well as crystallization, X-ray data acquisition, and structure solution, see the Supporting Information.

### 4.5 Molecular Modeling

For molecular modeling, the cocrystal structure of *E. coli* GyrB in complex with phosphoaminophosphonic acid–adenylate ester (PDB ID 4WUB)^24^ was used. Molecular docking calculations were performed in Schrödinger Release 2022-1 (Schrödinger, LLC, New York, NY, USA, 2022). MD simulation of compound **D** or compound **E** in complex with *E. coli* GyrB was performed using the NAMD package (version 2.9)^26^ and the CHARMM36m^27^ force field. Pharmacophore feature analysis of *E. coli* GyrB in complex with compound **D** or compound **E** was performed using LigandScout 4.4 Expert.^28^ For details on molecular docking, molecular dynamic simulations, and structure-based pharmacophore modeling, see the Supporting Information.

## Supporting Information Available

Additional experimental data, X-ray crystallography details, NMR spectra, and HPLC traces (PDF)

## Accession Codes

PDB Accession Codes—complex of *E. coli* GyrB24 with inhibitor **A**: 7P2N.

## Notes

The views expressed in this article are the views of the authors and neither IMI nor the European Union or EFPIA are responsible for any use that may be made of the information contained herein.

## Abbreviations

CCP4: collaborative computational project no. 4
DCM: dichloromethane
DMF: *N*,*N*-dimethylformamide
DTT: dithiothreitol
MIC: minimum inhibitory concentration
NAMD: nanoscale molecular dynamics
NPT: isothermal-isobaric ensemble
THF: tetrahydrofuran
TLC: thin-layer chromatography
