# Supplementary material for: Exploring the 5-Substituted 2-Aminobenzothiazole-Based DNA Gyrase B Inhibitors Active against ESKAPE Pathogens
Source: ACS Omega. 2023 Jun 28;8(27):24387–95. doi: 10.1021/acsomega.3c01930 (PMC10339456; doi:10.1021/acsomega.3c01930)
Supplement: Supplementary file 1 — ao3c01930_si_001.pdf [file ao3c01930_si_001.pdf]

Exploring the 5-substituted 2-aminobenzothiazole-based DNA gyrase B inhibitors active against ESKAPE pathogens

Mas☒a Sterle,[1] Martina Durcik,[1] Clare E. M. Stevenson,[2] Sara R. Henderson,[3] Petra Eva Szili,[4] Marton Czikkely,[4] David M. Lawson,[2] Anthony Maxwell,[2] Dominique Cahard,[5] Danijel Kikelj,[1] Nace Zidar,[1] Csaba Pal,[4] Lucija Peterlin Mas☒ic☒,[1] Janez Ilas☒,[1] Tihomir Tomas☒ic☒,[1] Andrej Emanuel Cotman,*,[1] Anamarija Zega*,[1]

[1] University of Ljubljana, Faculty of Pharmacy, Aškerčeva cesta 7, 1000 Ljubljana, Slovenia

[2] Department of Biochemistry and Metabolism, John Innes Centre, Norwich Research Park, Norwich NR4 7UH, U.K.

[3] Institute of Microbiology and Infection, College of Medical and Dental Sciences, University of Birmingham, B15 2TT, UK.

[4] Synthetic and Systems Biology Unit, Institute of Biochemistry, Biological Research Centre, Szeged H-6726, Hungary

[5] CNRS UMR 6014 COBRA, Normandie Université, 76821 Mont Saint Aignan, France

SUPPORTING INFORMATION

Table of contents

LC-MS and NMR analysis of side products of cyclization reaction 2

^{1}H, ^{19}F and ^{13}C NMR spectra, and LC-MS analysis of compounds. 9

Enzyme inhibition 31

X-ray Data Collection and Refinement Statistics 32

Experimental section 33

References 41

LC-MS and NMR analysis of side products of cyclization reaction

Scheme S1. Formation of the target compound 2a and acyclic compound 6a as the main side product.
No conversion of **6a** to **2a** was observed after prolonged reaction time at 22 °C or after heating the reaction mixture at 60 °C overnight. Only the identified side product **6a** is shown.

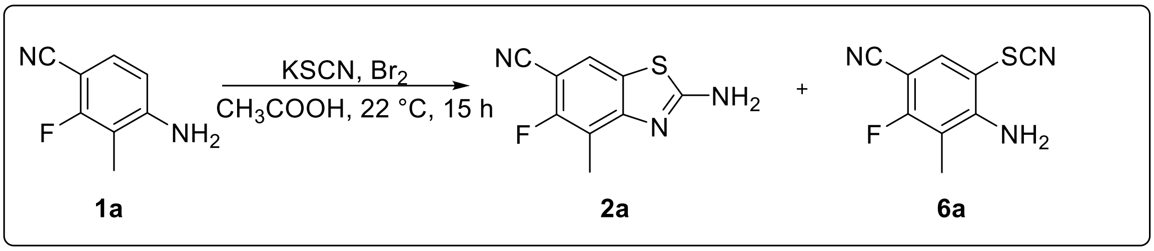

Figure S1. LC-MS and ^{1}H NMR analysis of the identified side products in synthesis of the target compound **2a**.

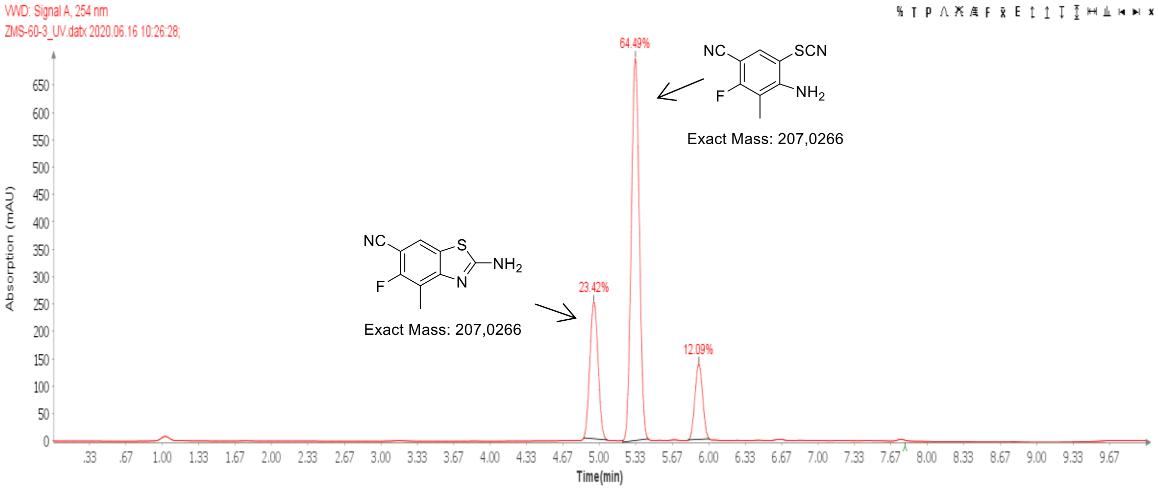

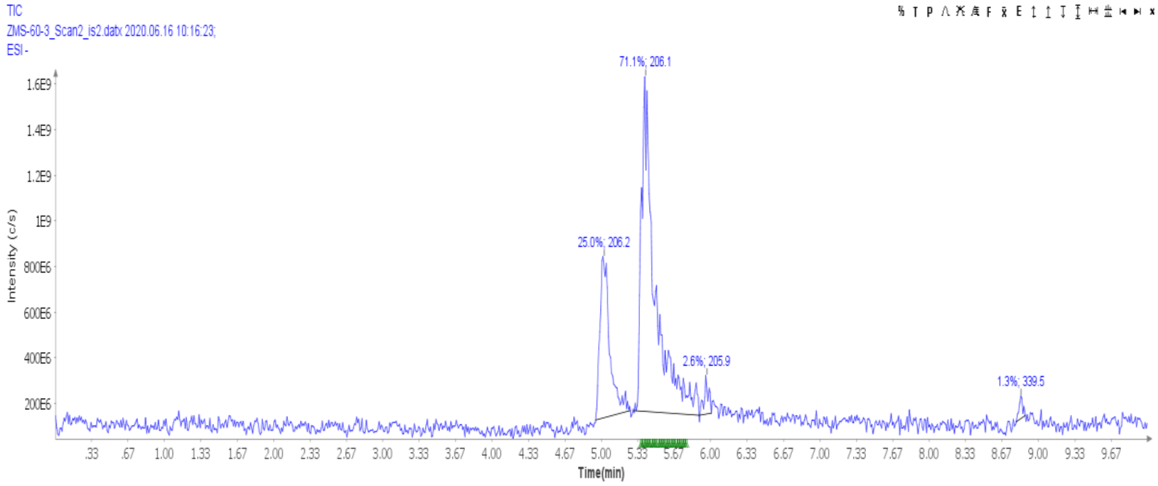

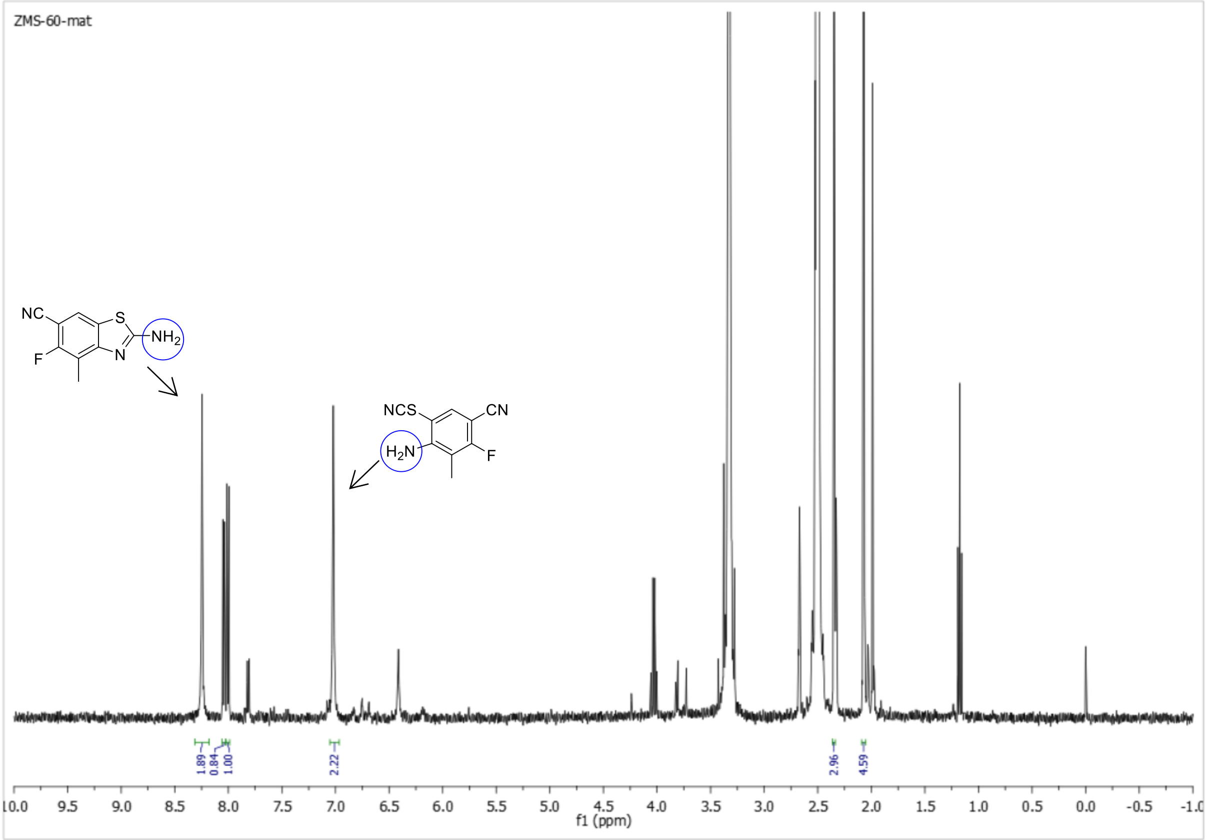
ZMS-60-mat
NC
S
NH2
F
N
NCS
CN
H2N
F
1.89
0.84
1.00
2.22
2.96
4.59
10.0 9.5 9.0 8.5 8.0 7.5 7.0 6.5 6.0 5.5 5.0 4.5 4.0 3.5 3.0 2.5 2.0 1.5 1.0 0.5 0.0 -0.5 -1.0
f1 (ppm)

Scheme S2. Formation of the target compound 2b and the two main side products 3b and 4b.
According to LC-MS analysis, the side products **3b** and **4b** were identified with additional Br- and SCN-substituent, respectively.

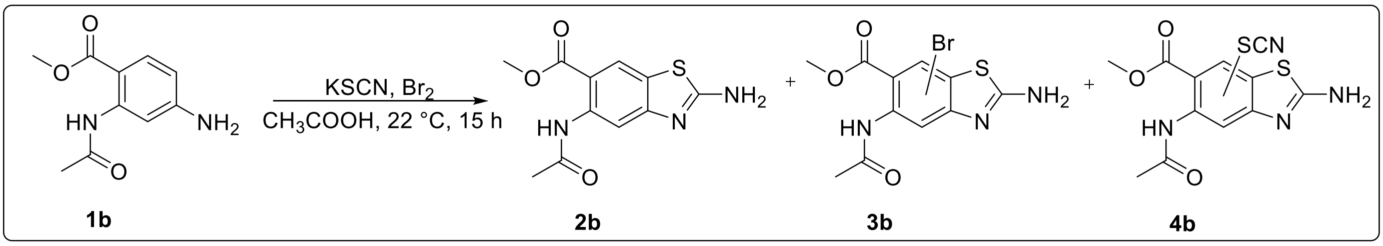

Figure S2. LC-MS analysis of the side products in synthesis of the target compound **2b**.

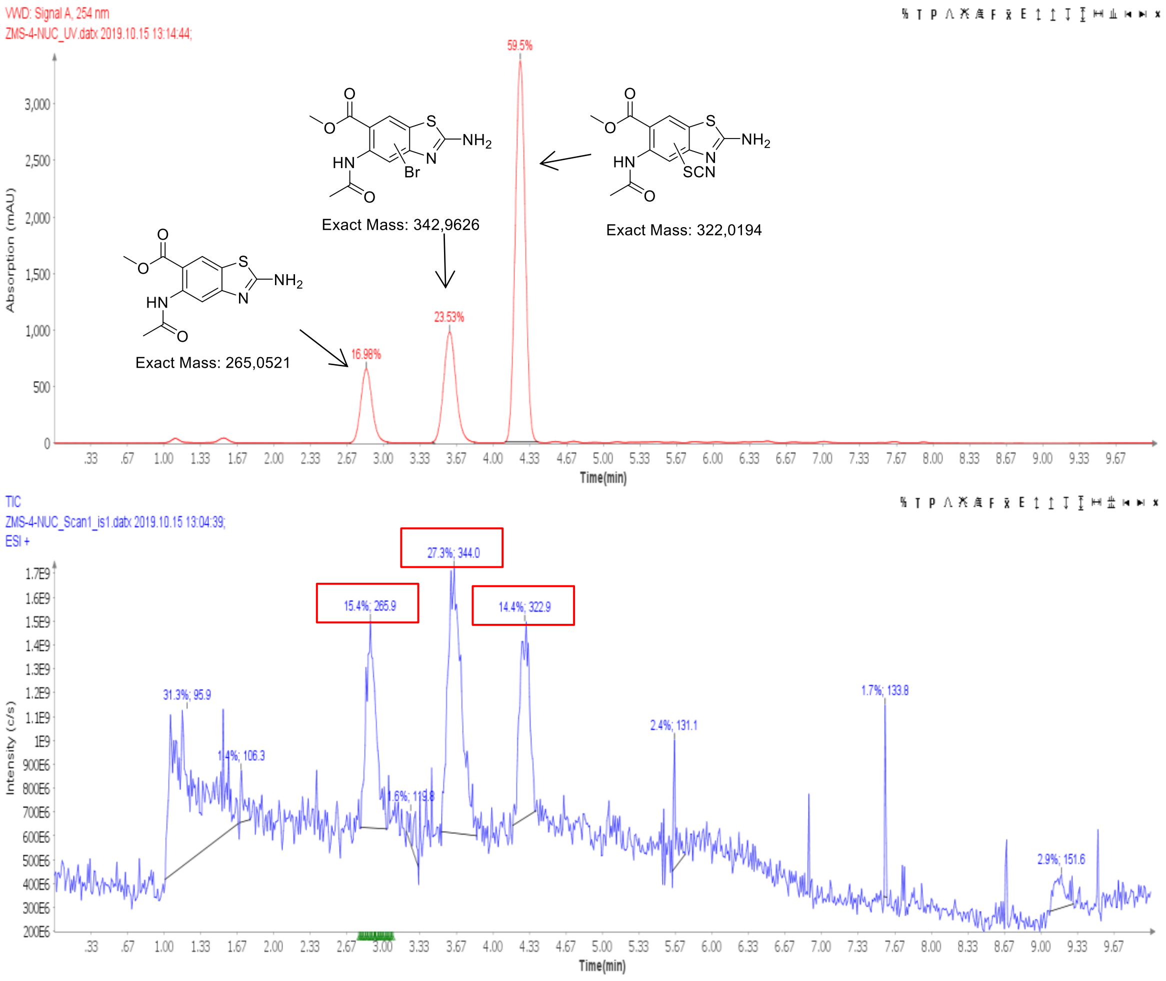

Scheme S3. Formation of the target compound 2c and its regioisomer 5c.

Both regioisomers are formed because of low difference in steric hindrance of respective ortho positions of aniline **1c**.

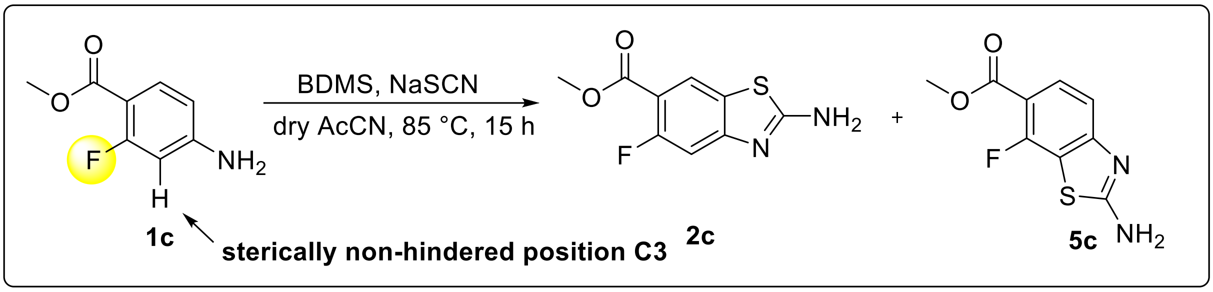

Figure S3. LC-MS and NMR analysis of side products in synthesis of target compound **2c**.

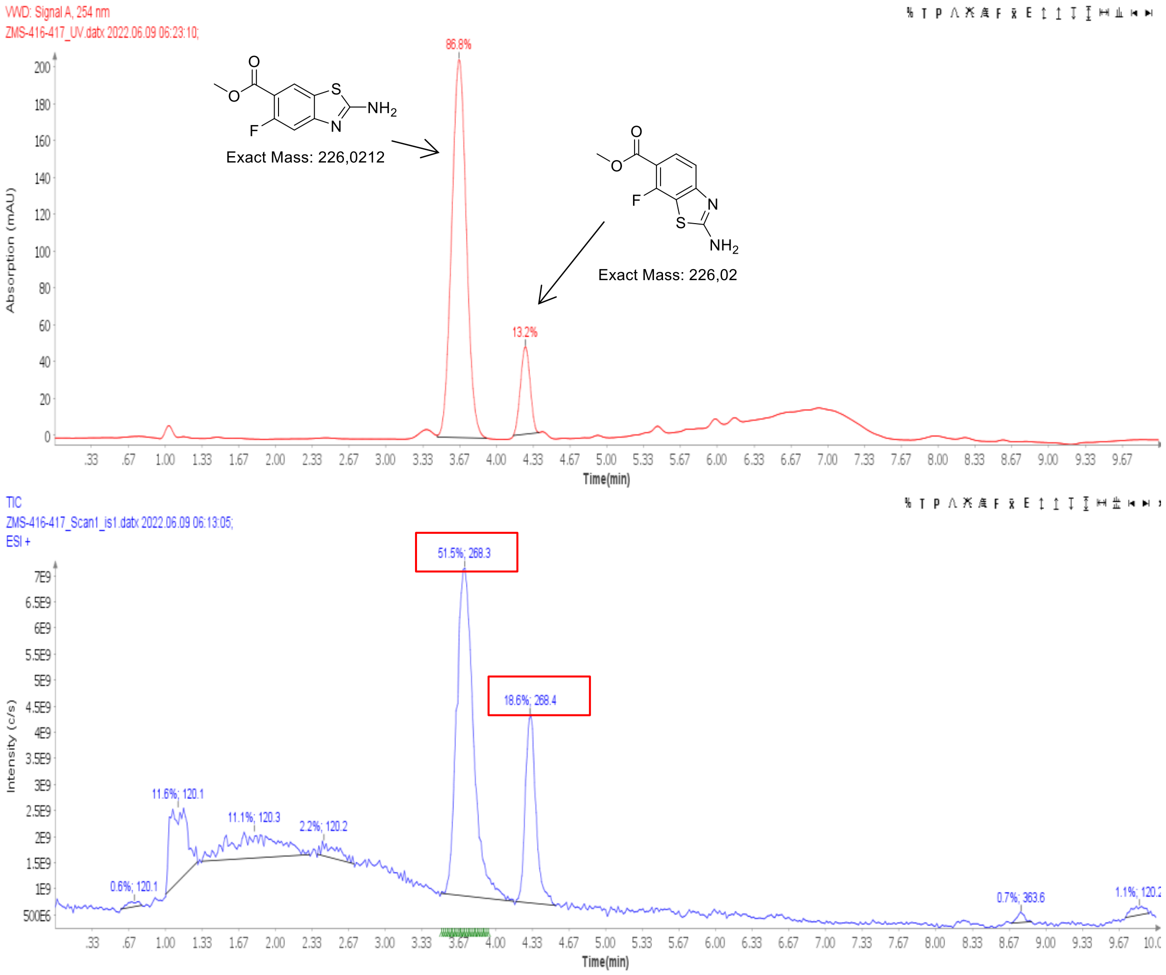

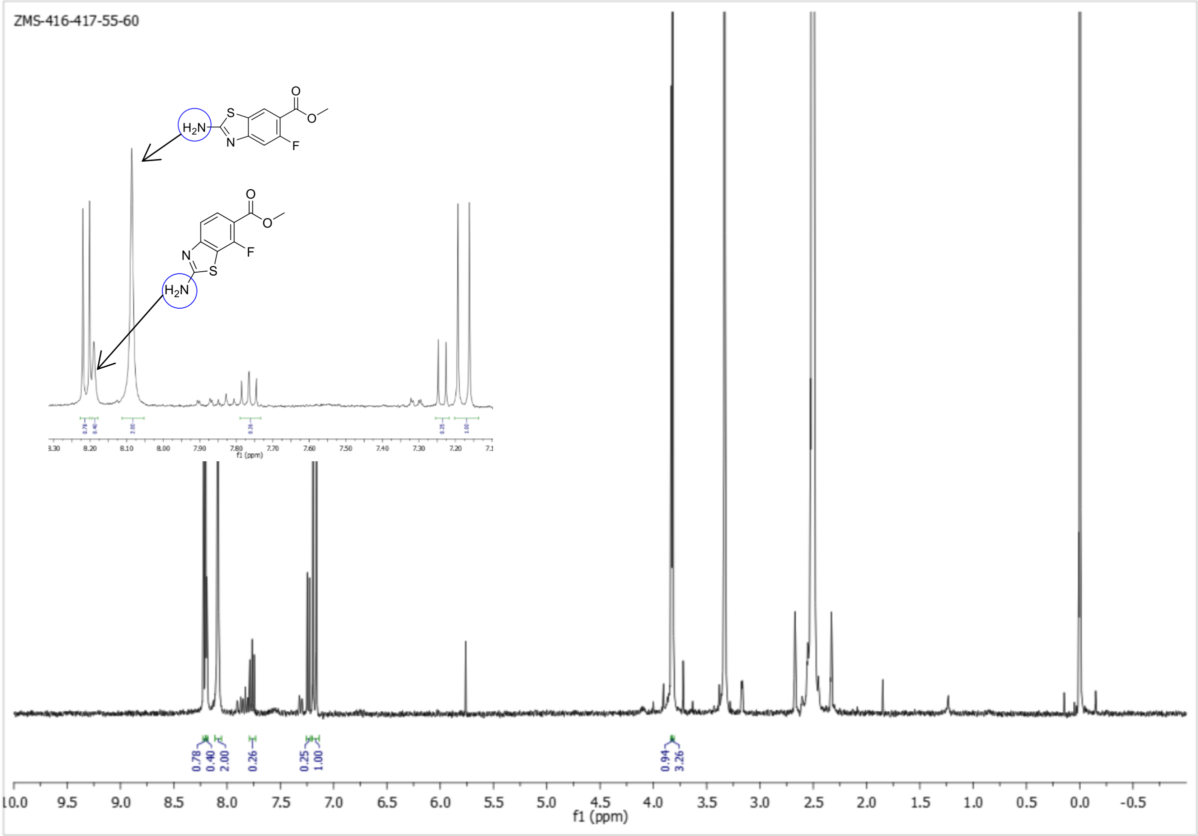
ZMS-416-417-55-60
H2N
S
N
O
O
F
f1 (ppm)

Scheme S4. Formation of the target compound 2d and its main side product 4d.

According to LC-MS analysis, the side product **4d** with additional SCN-substituent was identified. Bulky substituent at C2 of the starting compound **1a** blocks the formation of regioisomer **5d**.

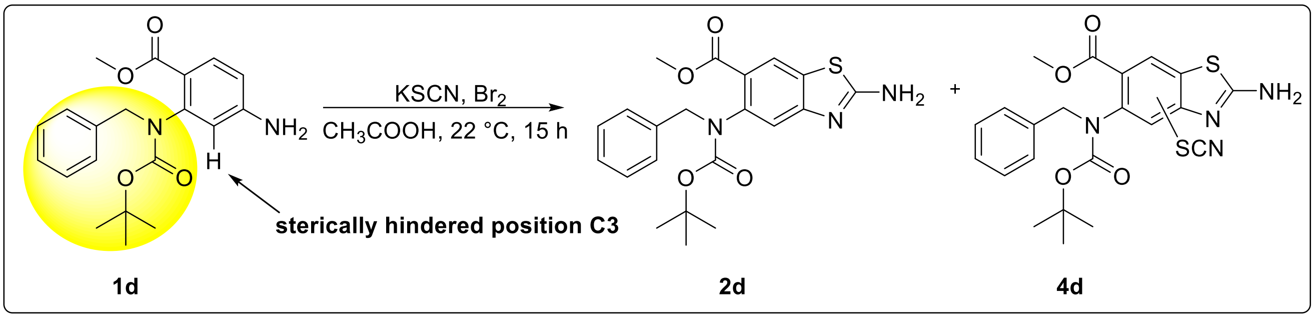

Figure S4. LC-MS analysis of the side products in synthesis of the target compound **2d**.

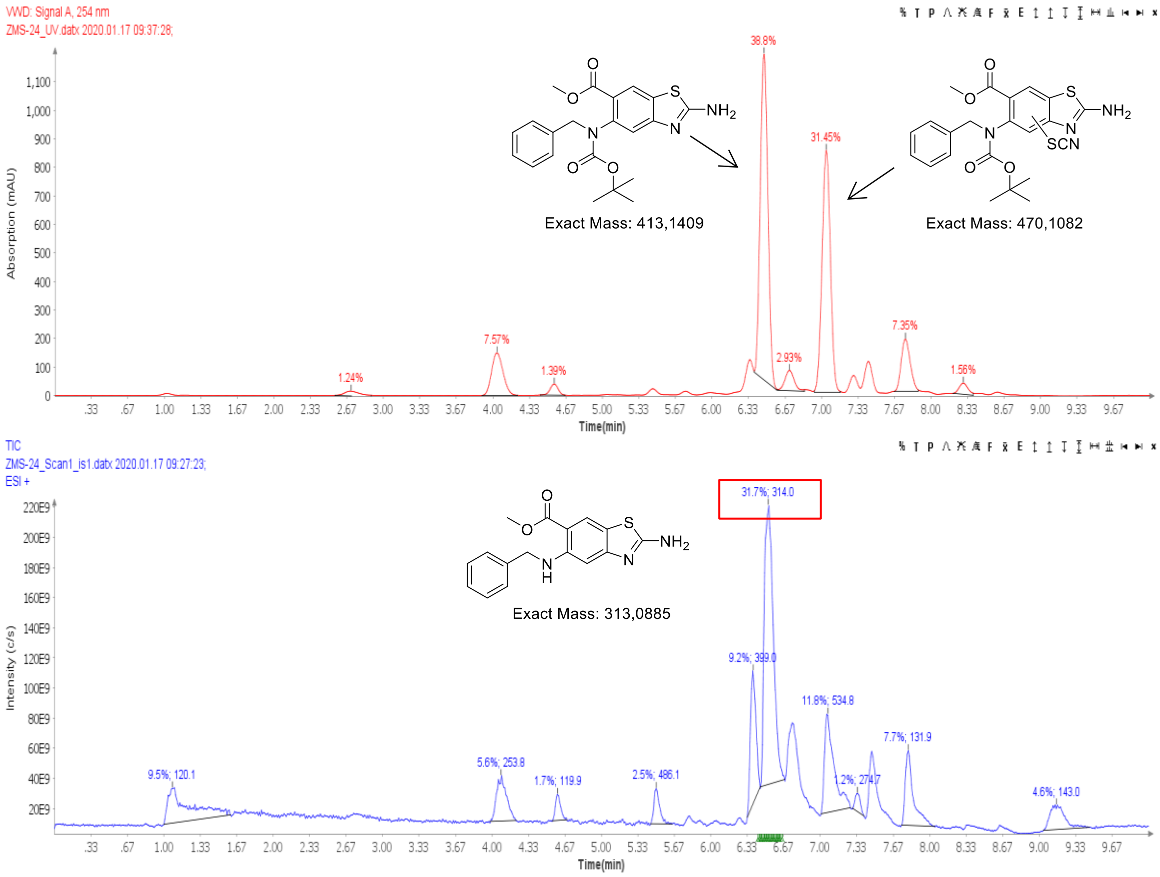

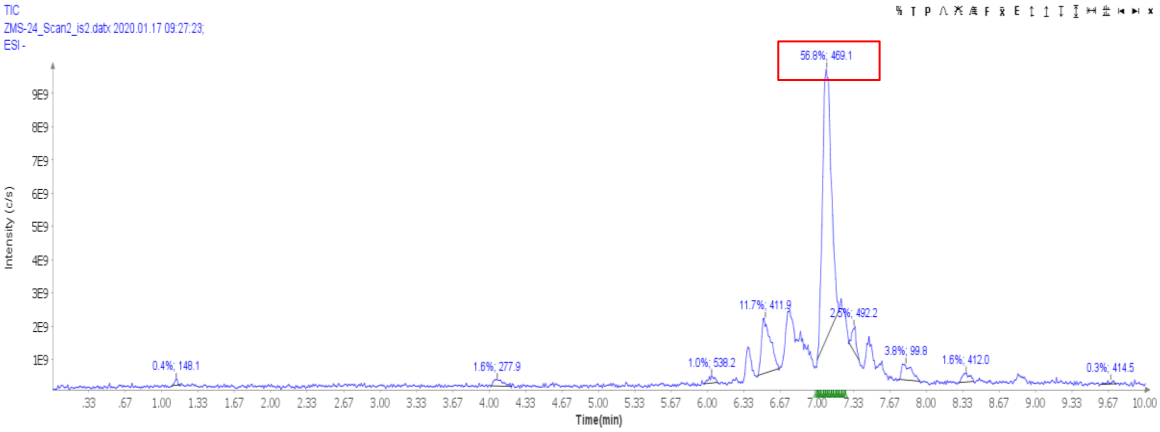
TIC
ZMS-24_Scan2_is2.datx 2020.01.17 09:27:23;
ESI -
56.8%; 469.1
11.7%; 411.9
2.5%; 492.2
3.8%; 99.8
1.6%; 412.0
0.3%; 414.5
1.0%; 538.2
1.6%; 277.9
0.4%; 148.1
Intensity (c/s)
Time(min)

^{1}H, ^{19}F and ^{13}C NMR spectra, and LC-MS analysis of compounds.

Compound **2c**, ^{1}H NMR:

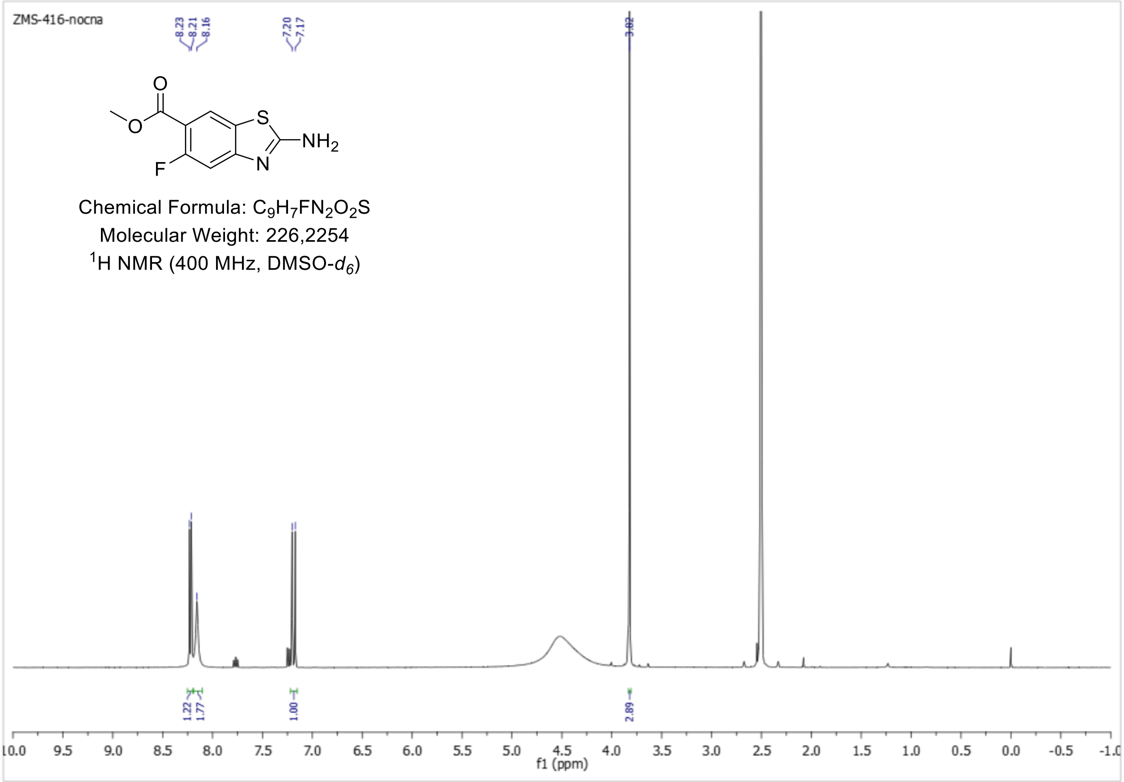

Compound **2c**, ^{19}F NMR:

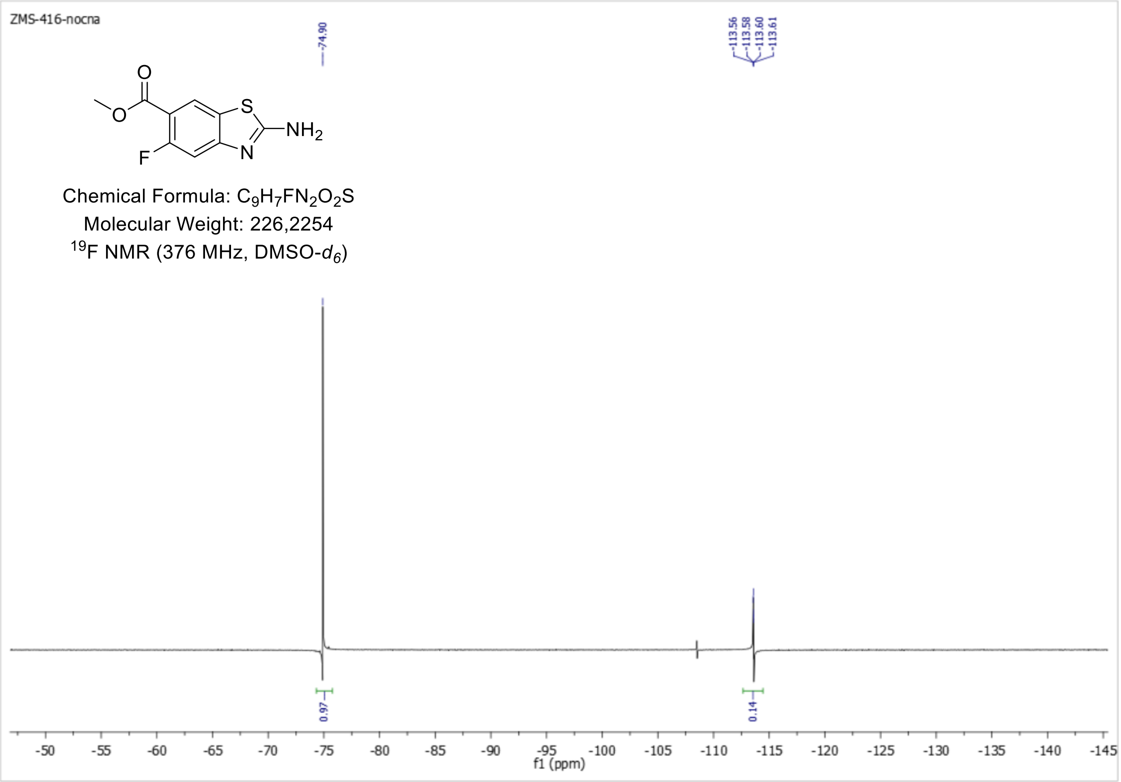

Compound **2c**, ^{13}C NMR:

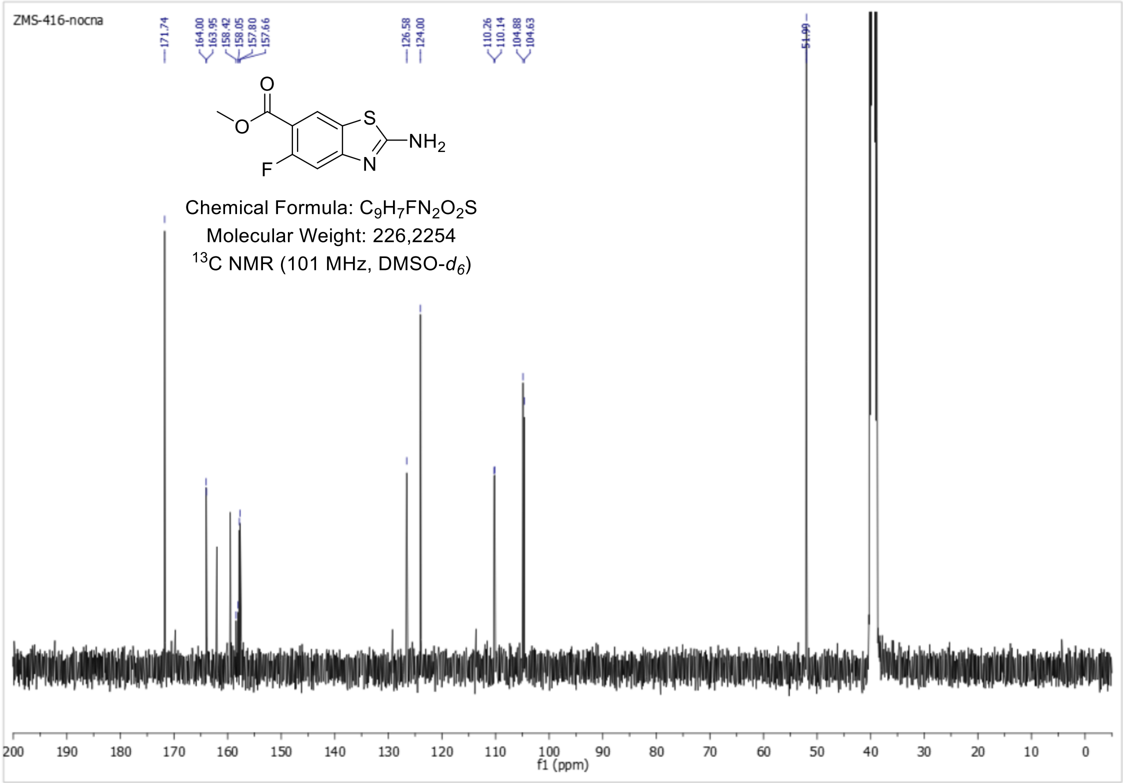

Compound **2e**, ^{1}H NMR:

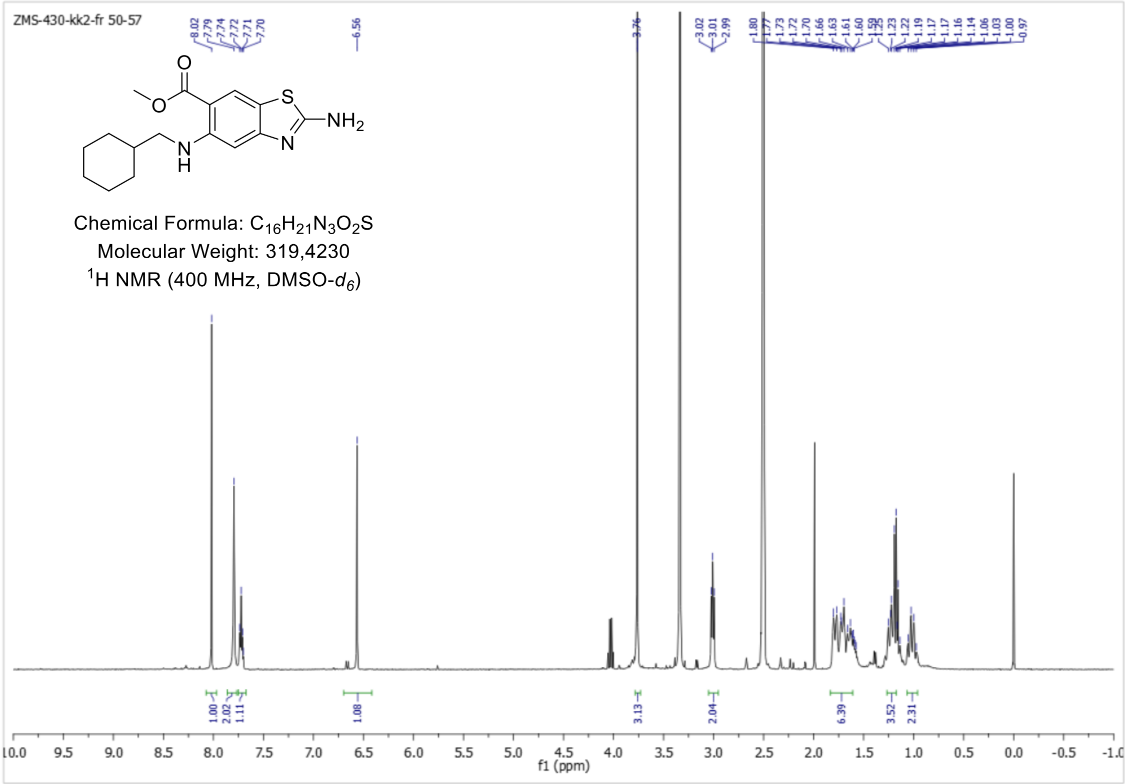

Compound **2e**, ^{13}C NMR:

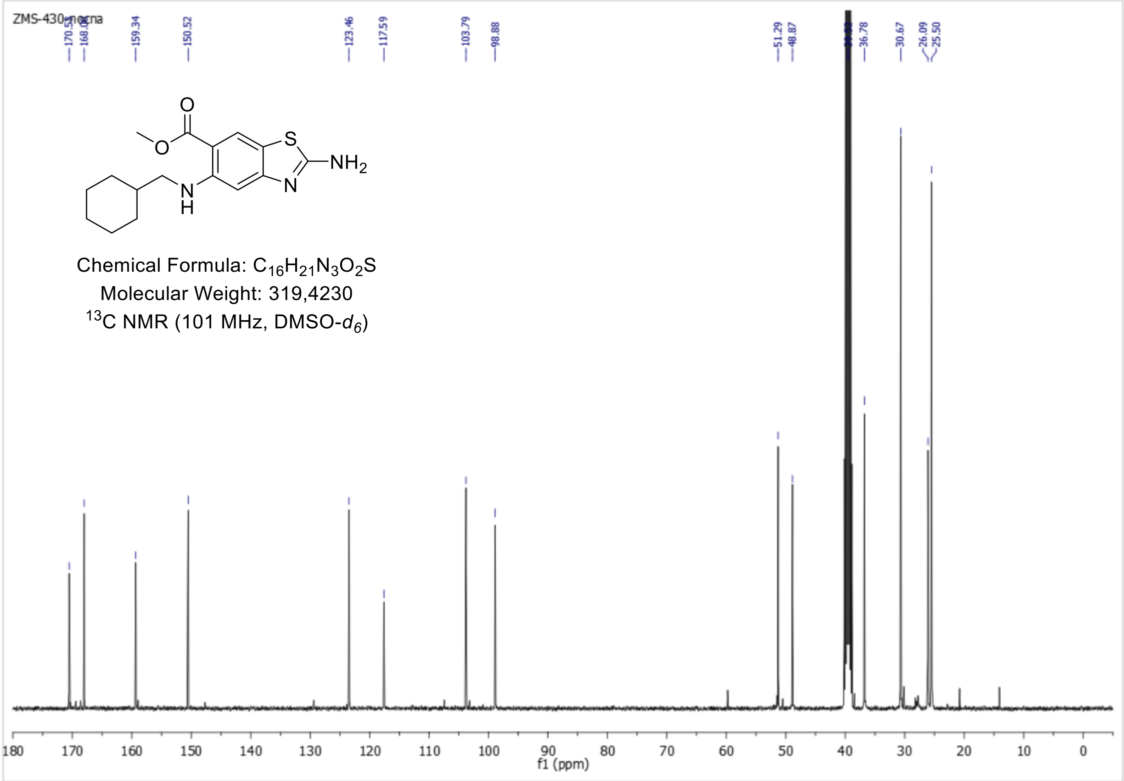

Compound **2a**, ^{1}H NMR:

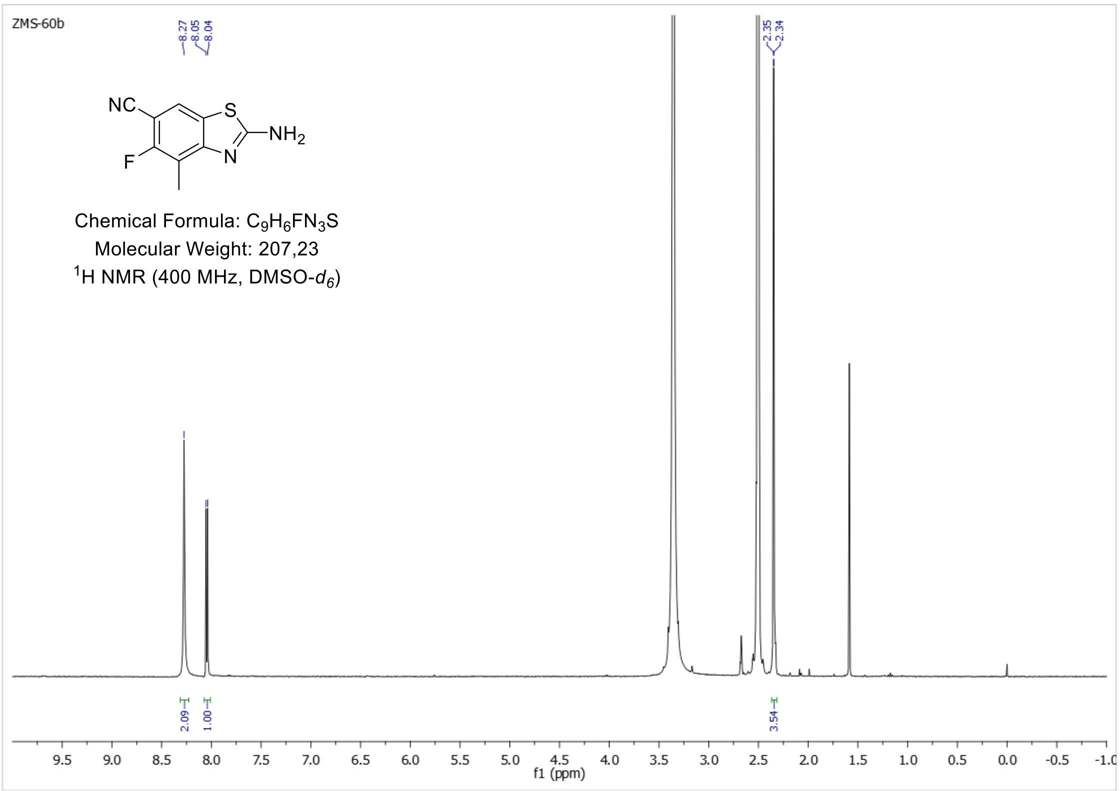

Compound **2a**, ^{19}F NMR:

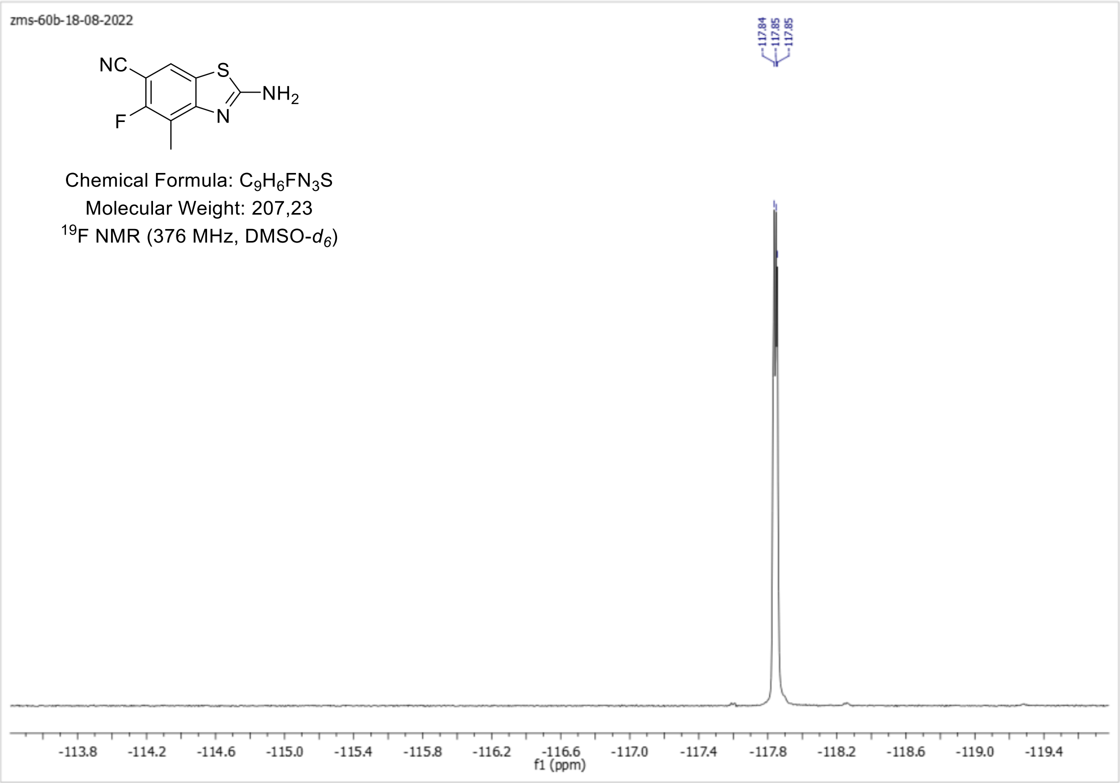

Compound **7**, ^{1}H NMR:

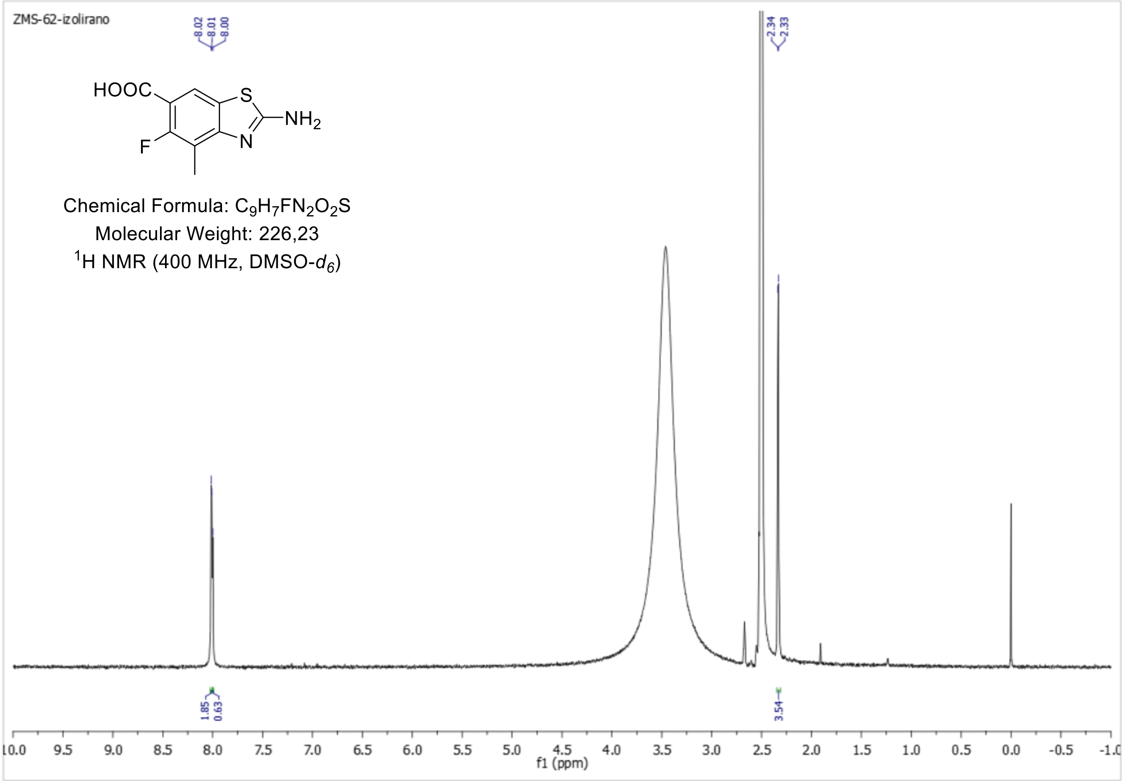

Compound **7**, ^{19}F NMR:

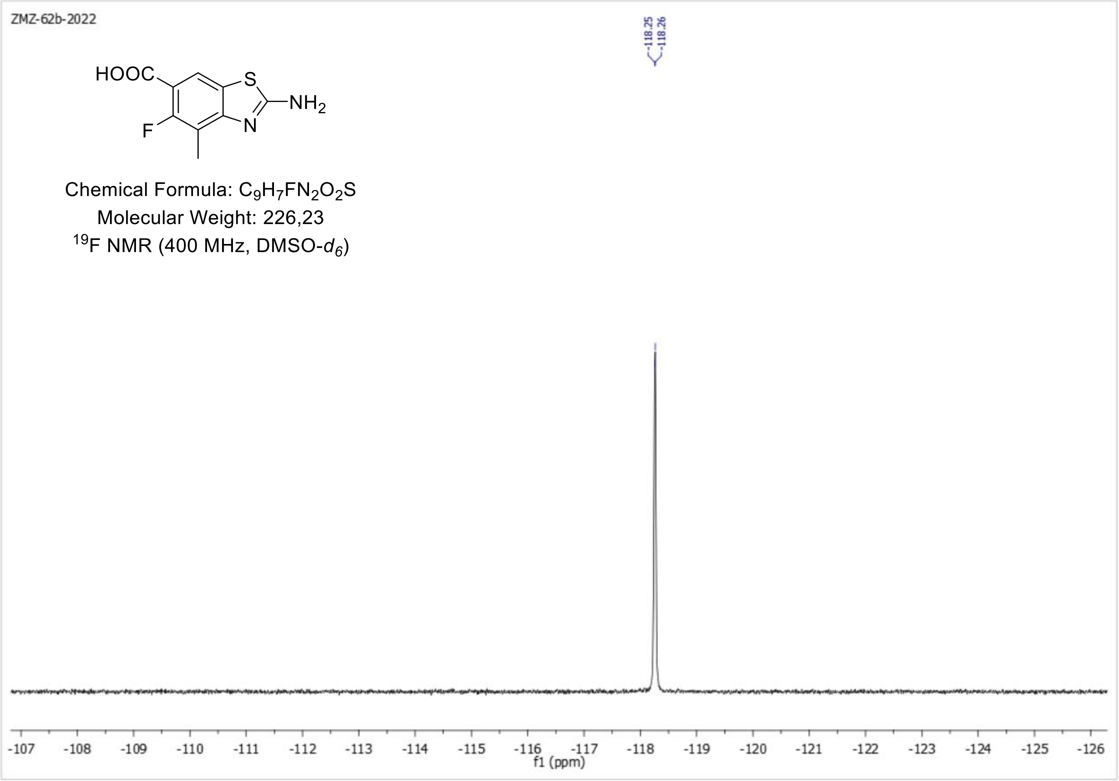

Compound **8**, ^{1}H NMR:

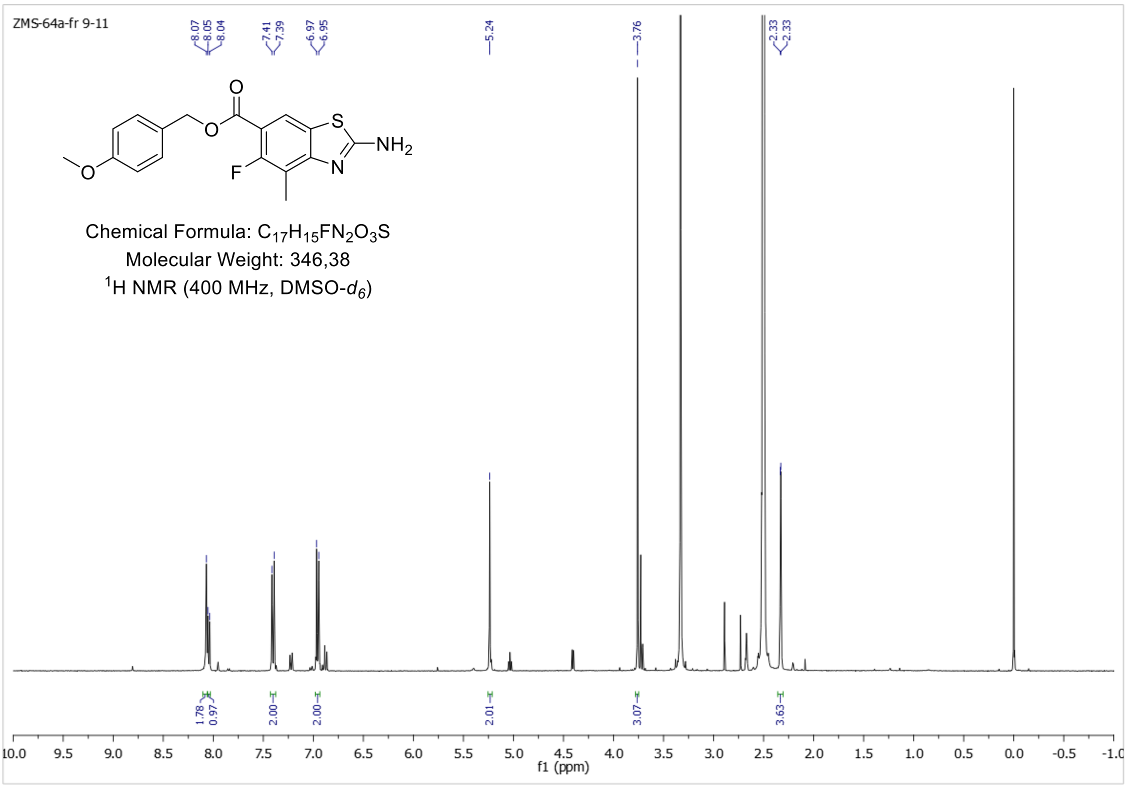

Compound **9**, ^{1}H NMR:

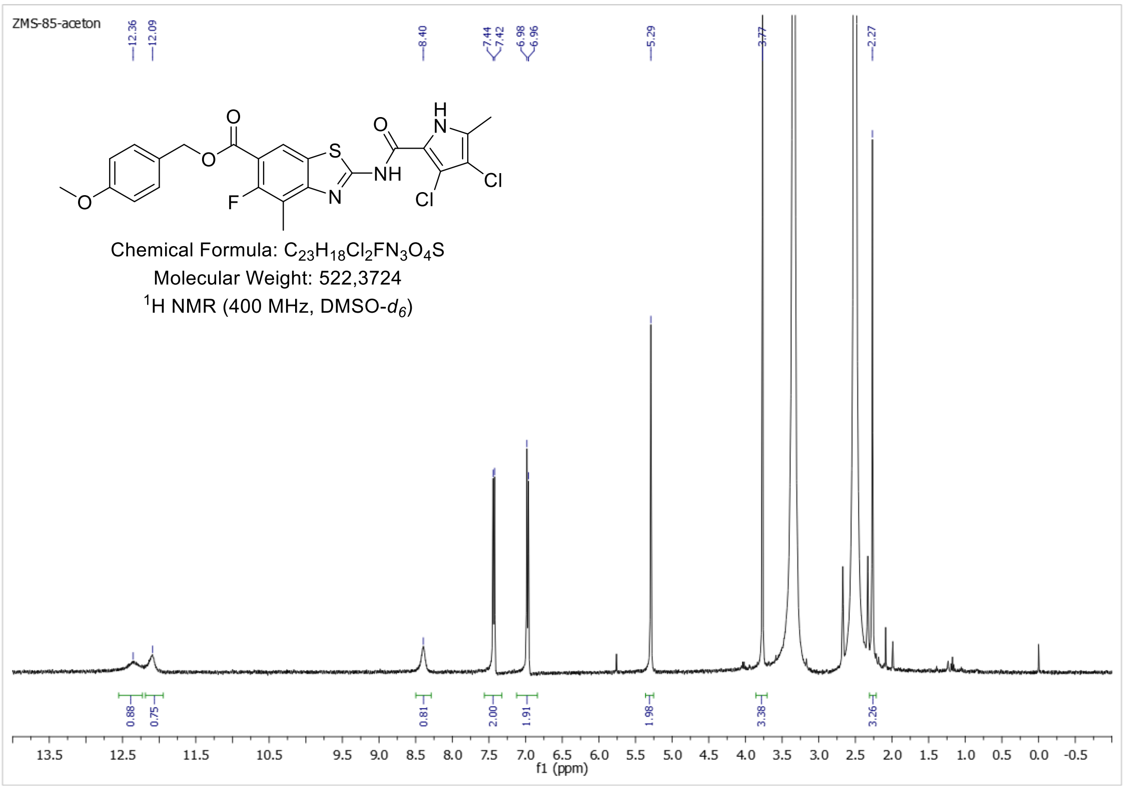

Compound **9**, ^{19}F NMR:

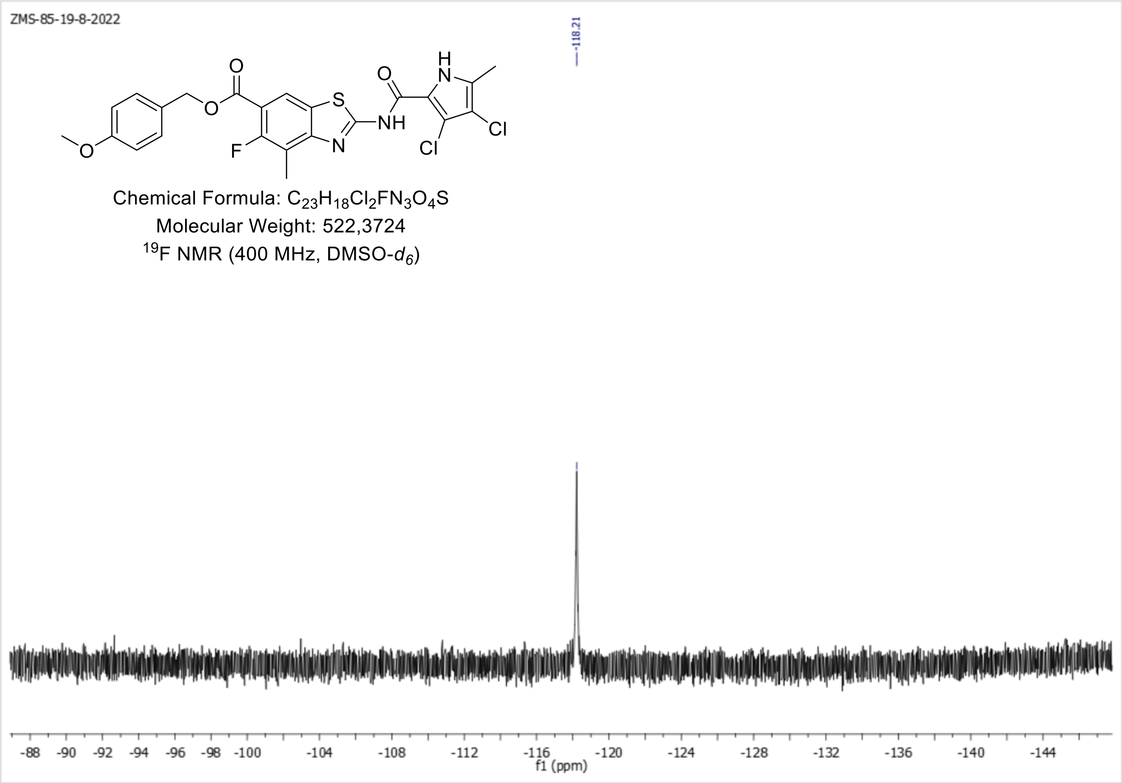

Compound **B**, ^{1}H NMR:

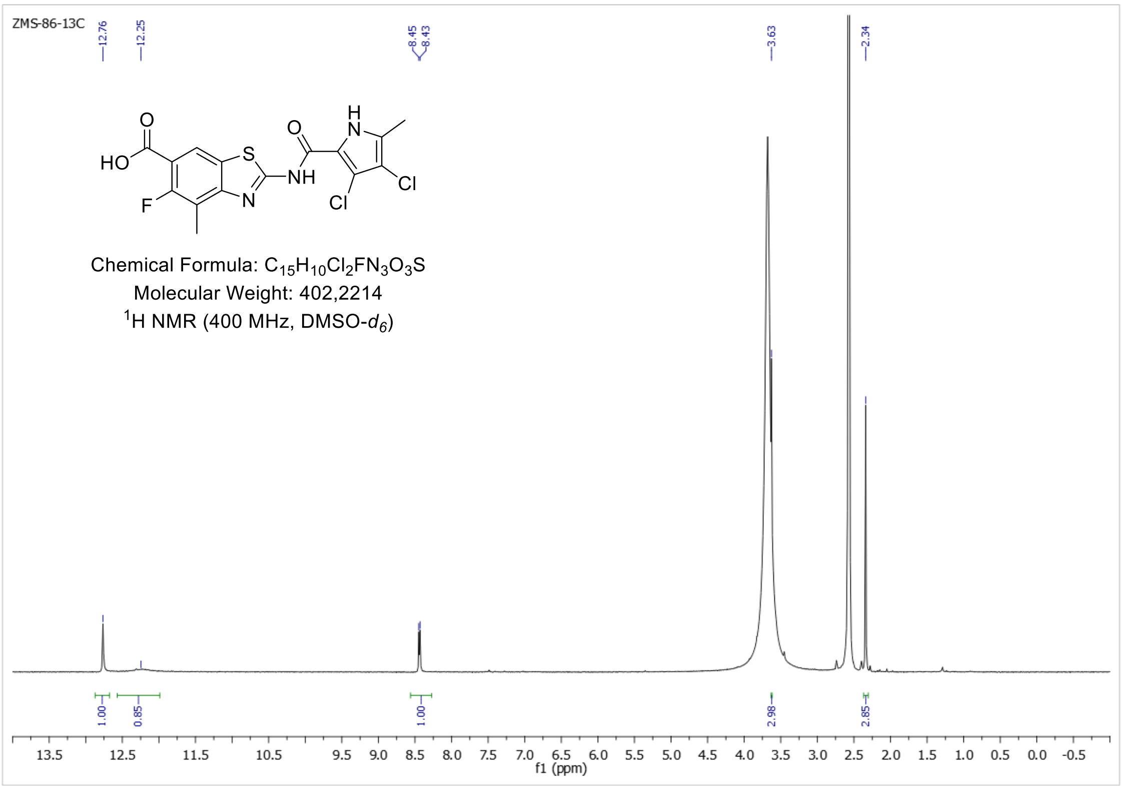

Compound **B**, ^{19}F NMR:

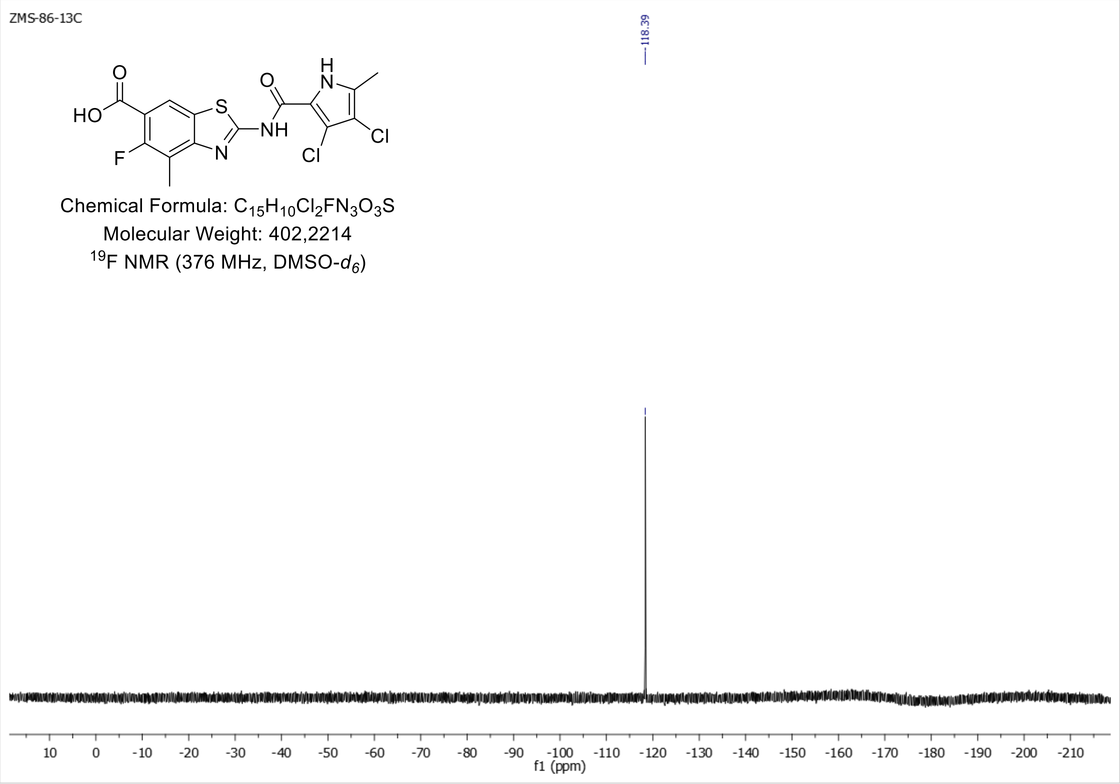

Compound **B**, ^{13}C NMR:

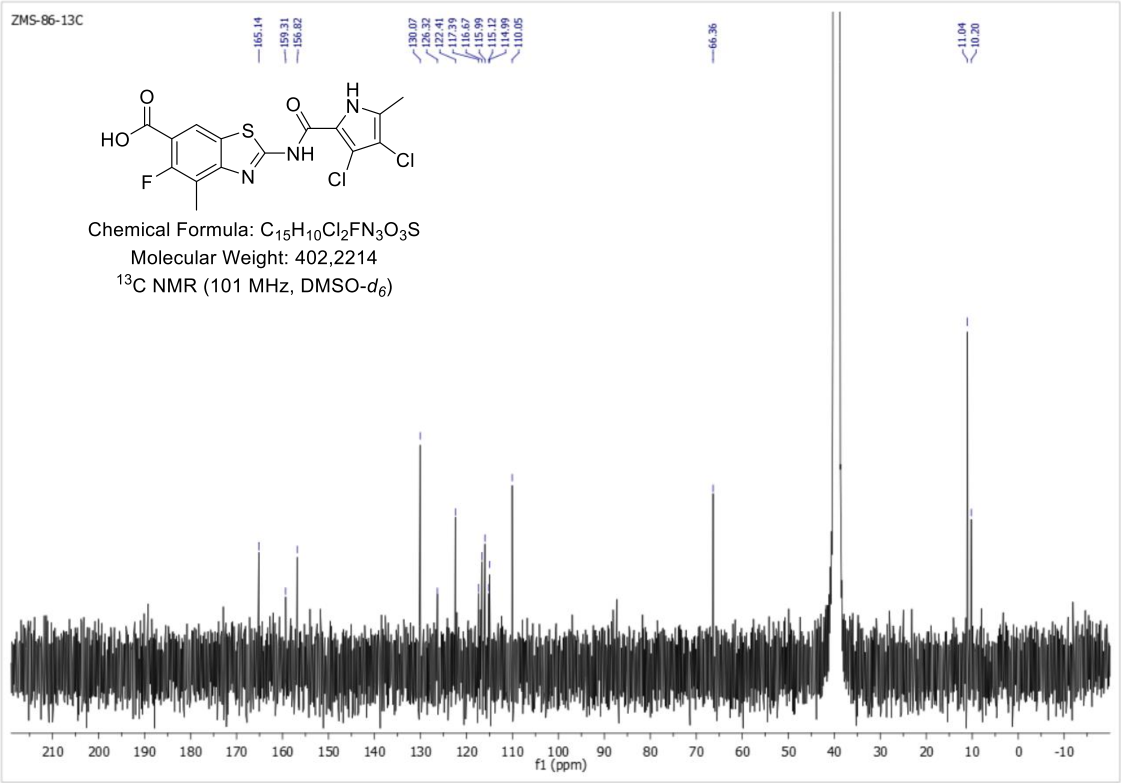

Compound **B**, HPLC trace:

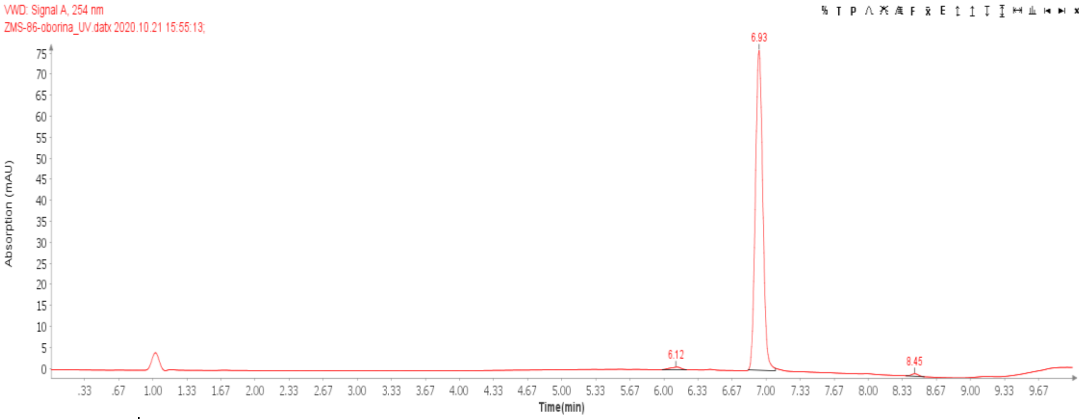

Peak	t_r (min)	Area	Area %
**1**	6.12	4.31	1.1
**2**	6.45	0.78	0.2
**B**	6.93	384.95	98.0
**3**	8.45	2.87	0.7

Compound **10**, ^{1}H NMR:

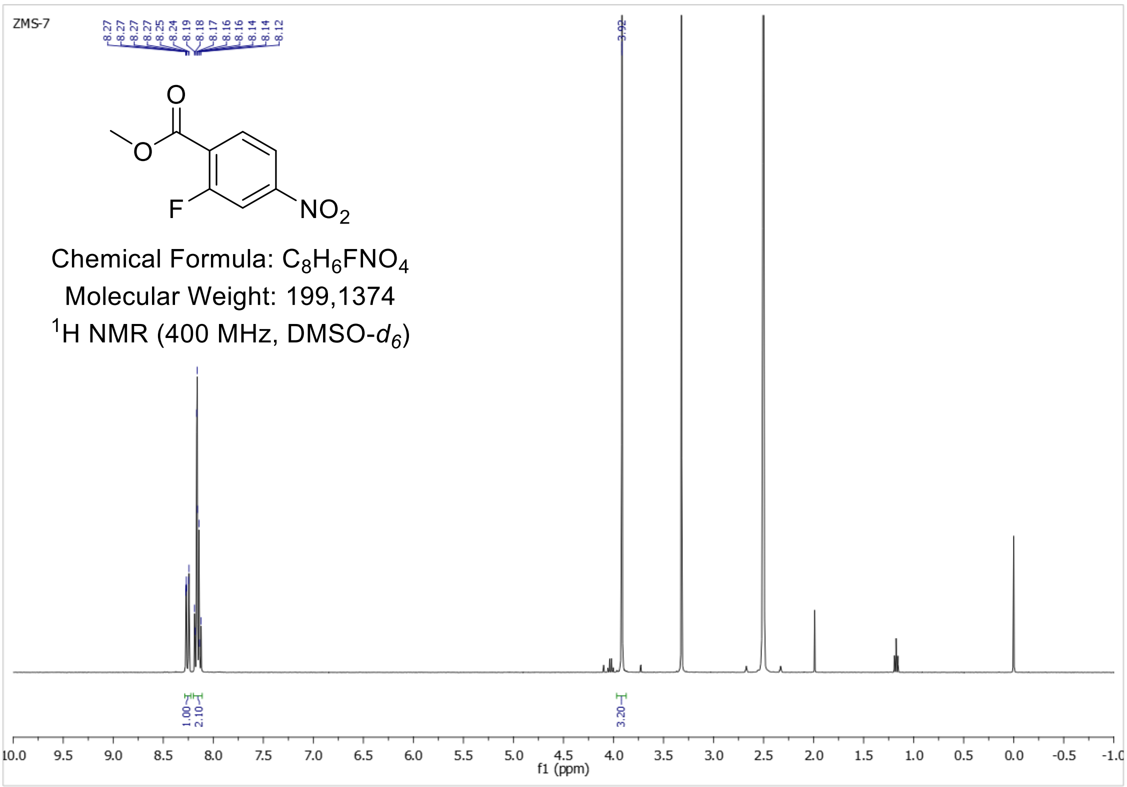

*Traces of ethyl acetate at 1.99 ppm, 4.03 ppm and 1.17 ppm.

Compound **11**, ^{1}H NMR:

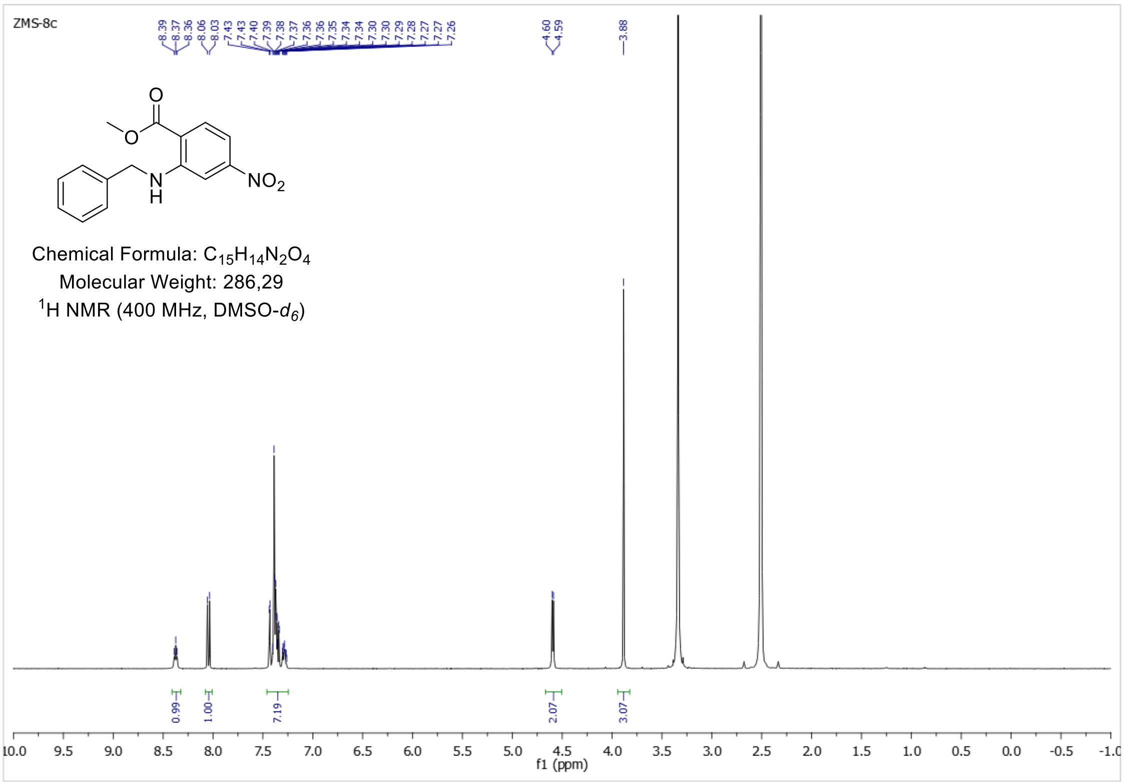

Compound **12**, ^{1}H NMR:

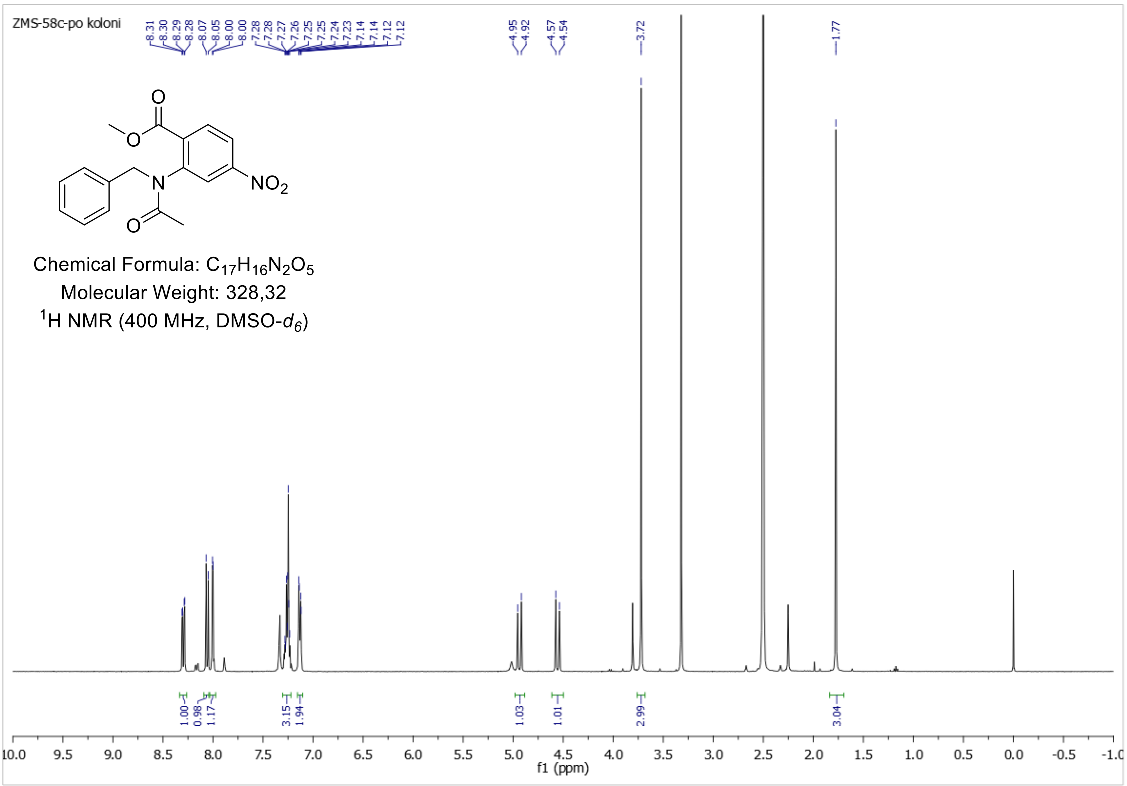

Compound **1f**, ^{1}H NMR:

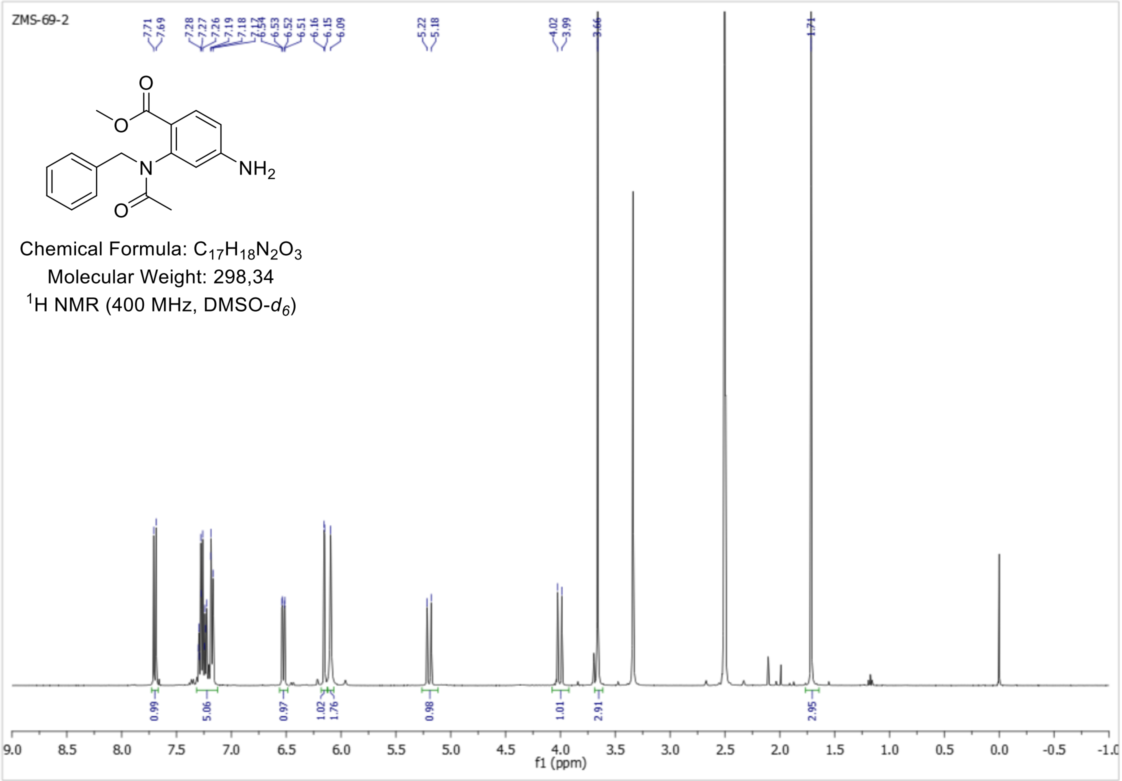

Compound **2f**, ^{1}H NMR:

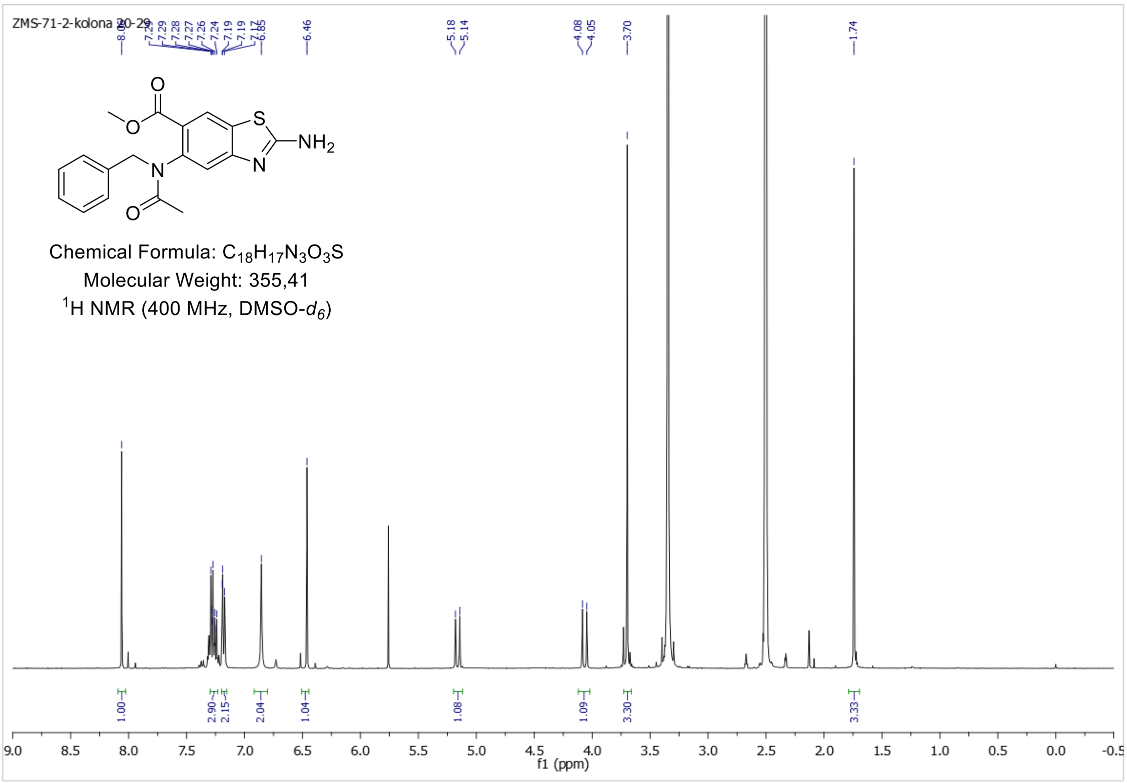

Compound **13**, ^{1}H NMR:

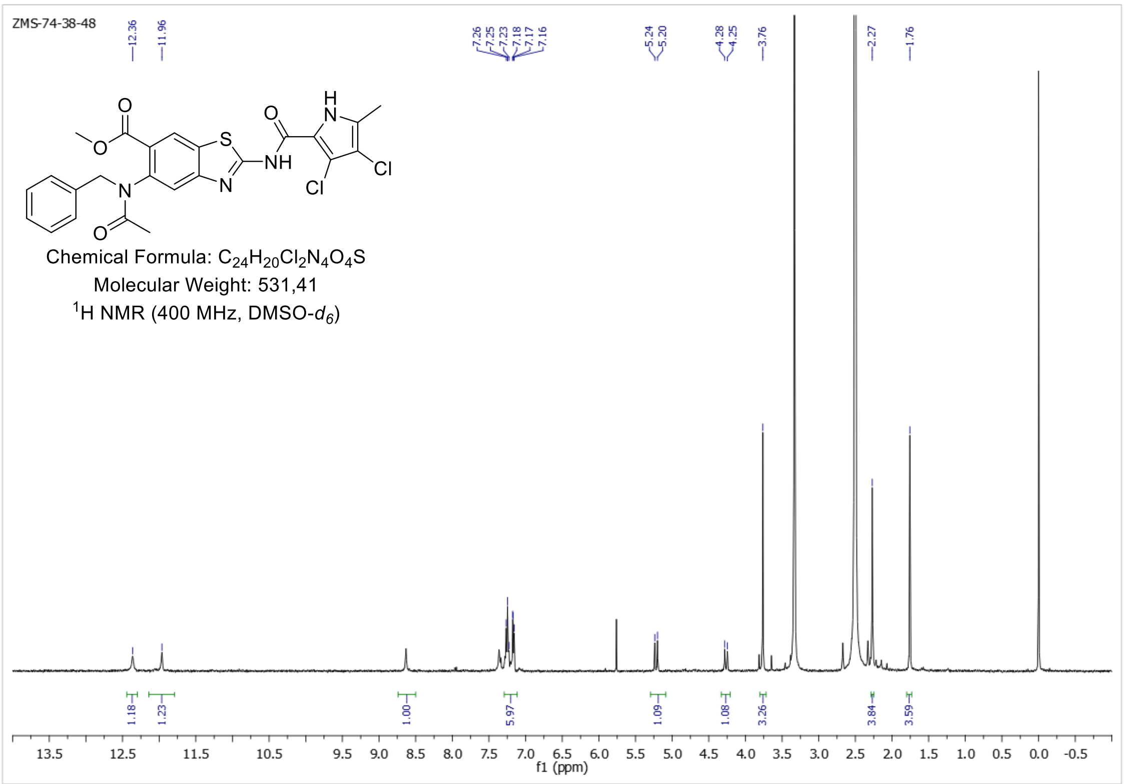

Compound **C**, ^{1}H NMR:

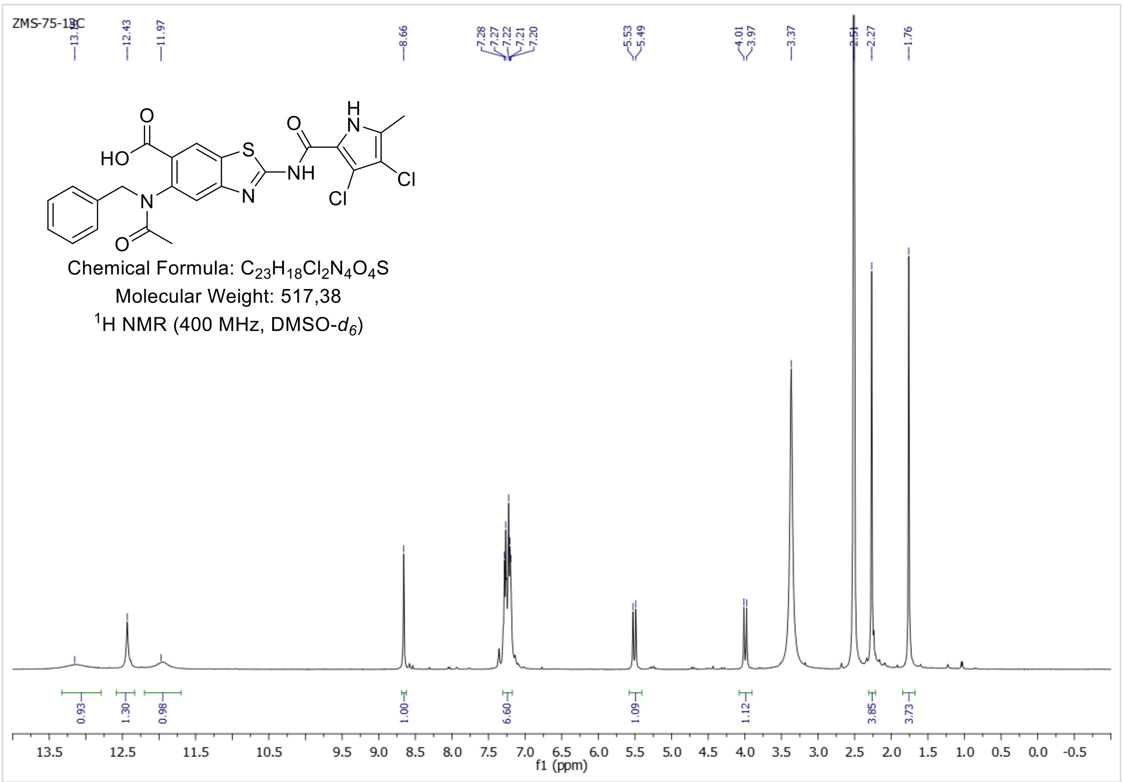

Compound **C**, ^{13}C NMR:

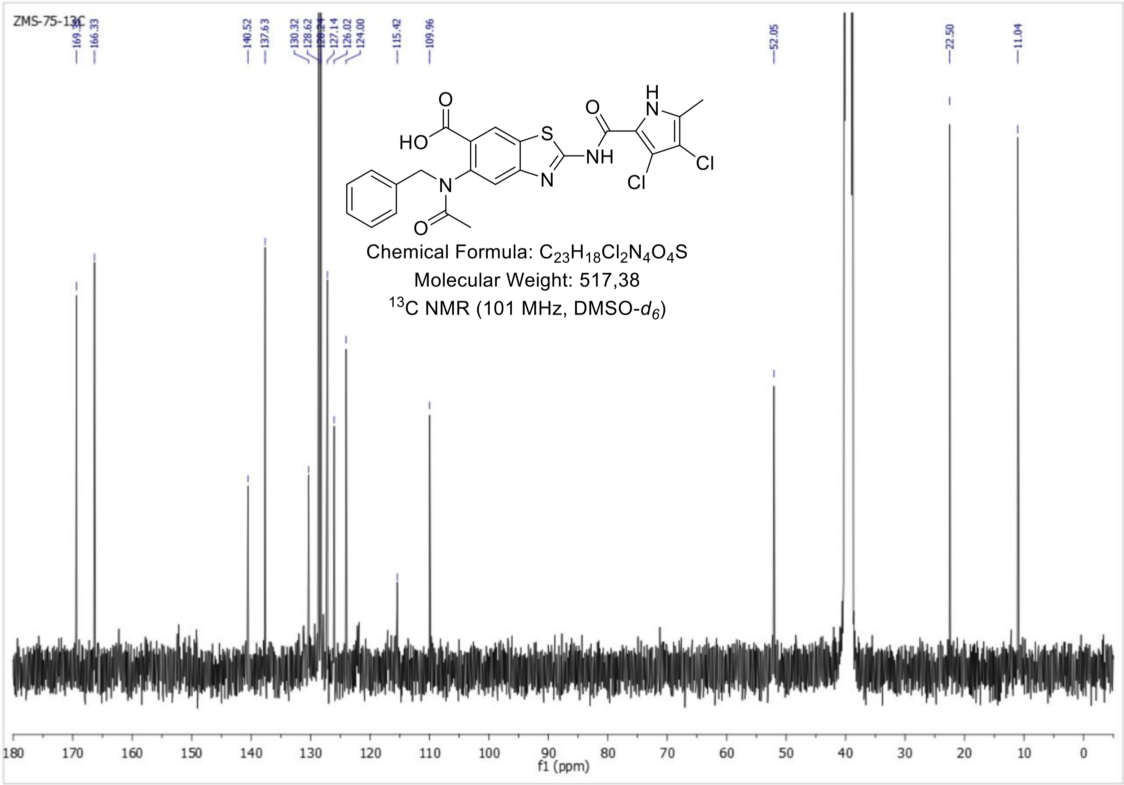

Compound **C**, HPLC trace:

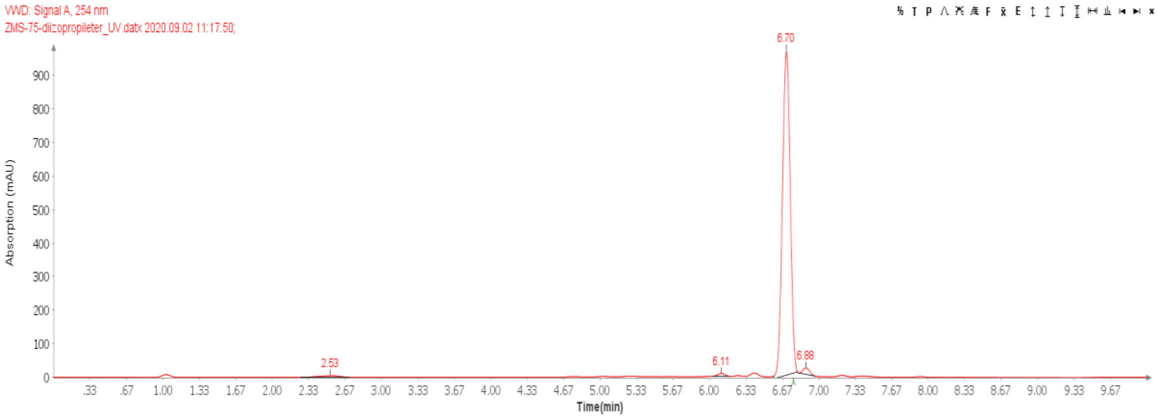

Peak	t_r (min)	Area	Area %
**1**	2.56	55.38	1.1
**2**	6.11	35.26	0.7
**3**	6.41	40.93	0.8
**C**	6.70	4806.13	95.7
**4**	6.88	82.34	1.6

Compound **15**, ^{1}H NMR:

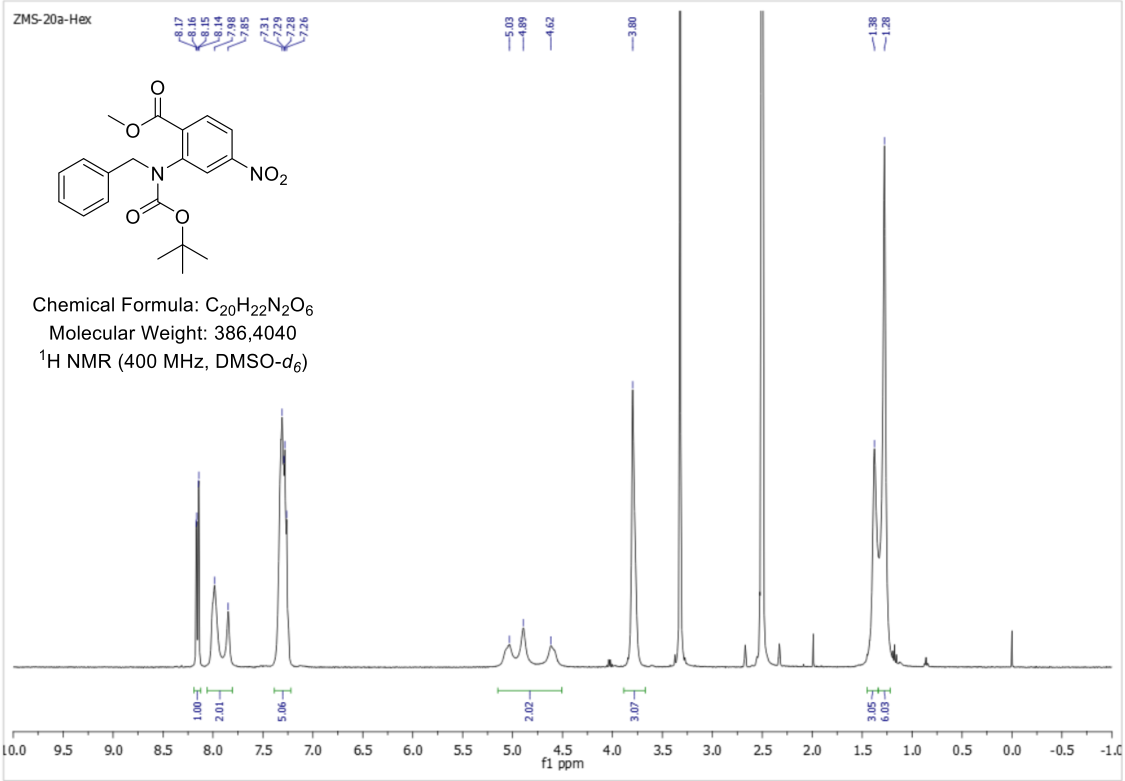

Compound **1d**, HPLC trace:

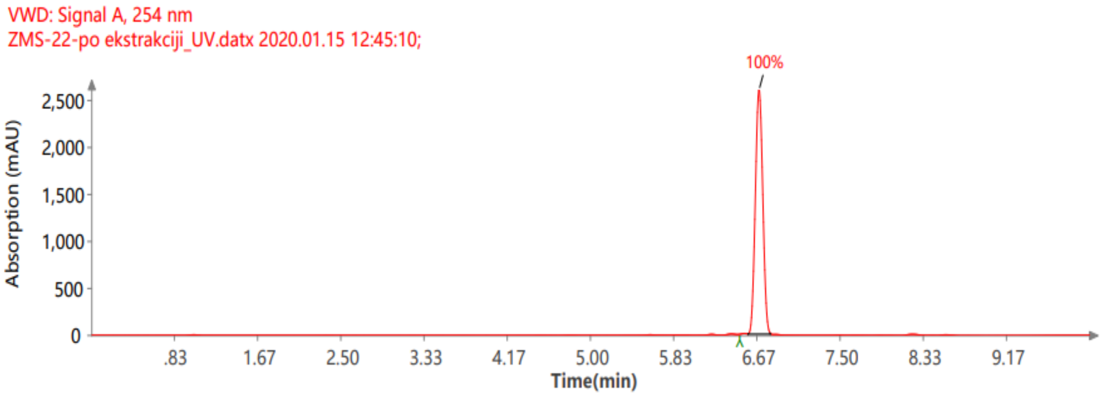

Peak	t_r (min)	Area	Area %
**1**	6.69	13415	100

Compound **2d**, ^{1}H NMR:

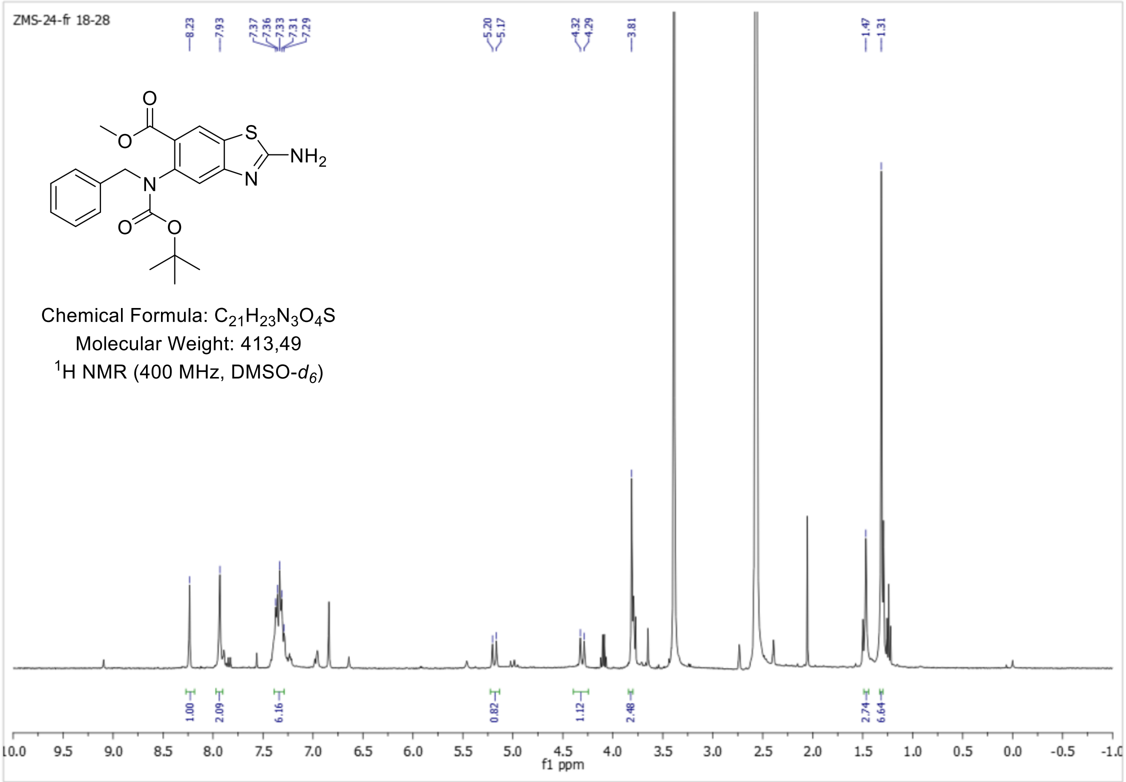

*Traces of ethyl acetate at 1.99 ppm, 4.03 ppm and 1.17 ppm.

Compound **17**, ^{1}H NMR:

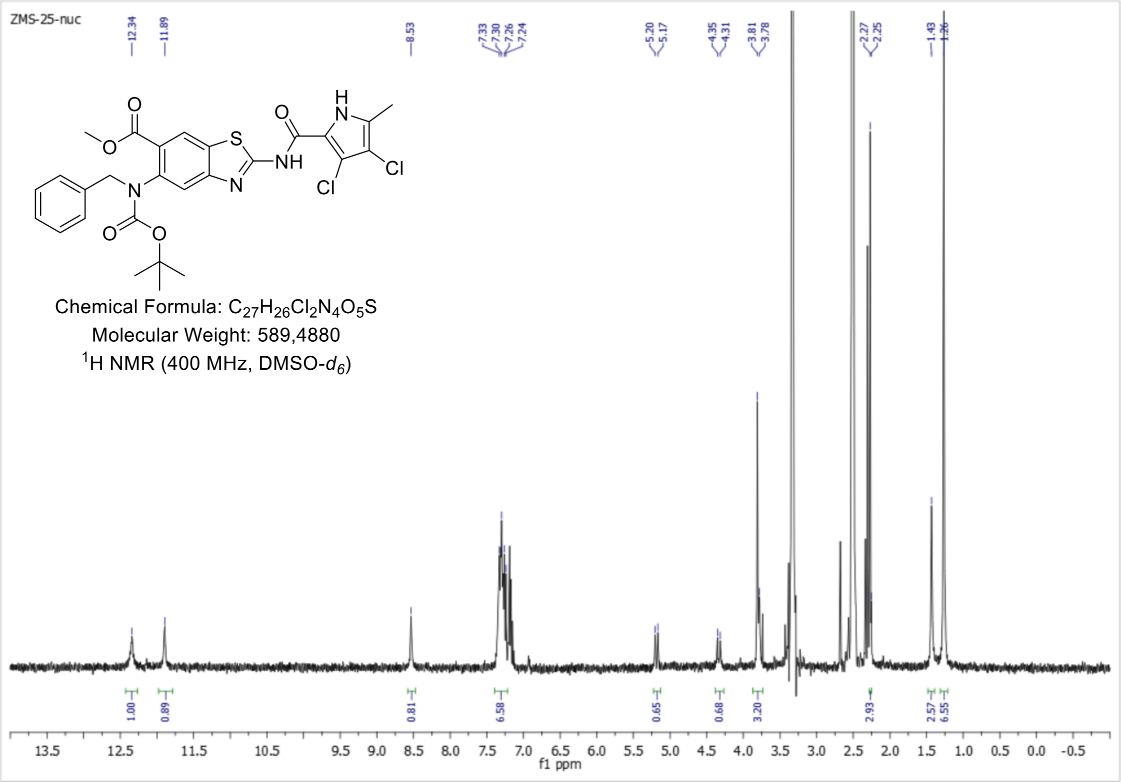

*Traces of toluene at 2.30 ppm, 7.18 ppm and 7.25 ppm.

Compound **19**, ^{1}H NMR:

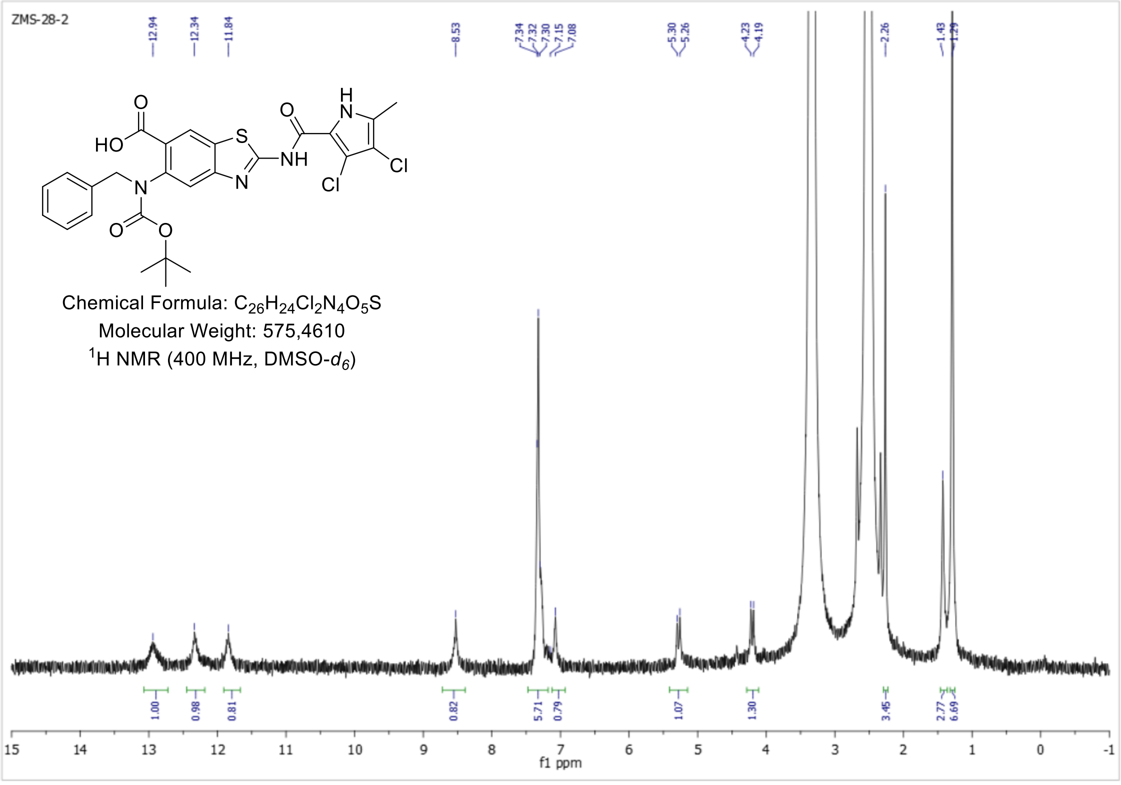

Compound **D**, ^{1}H NMR:

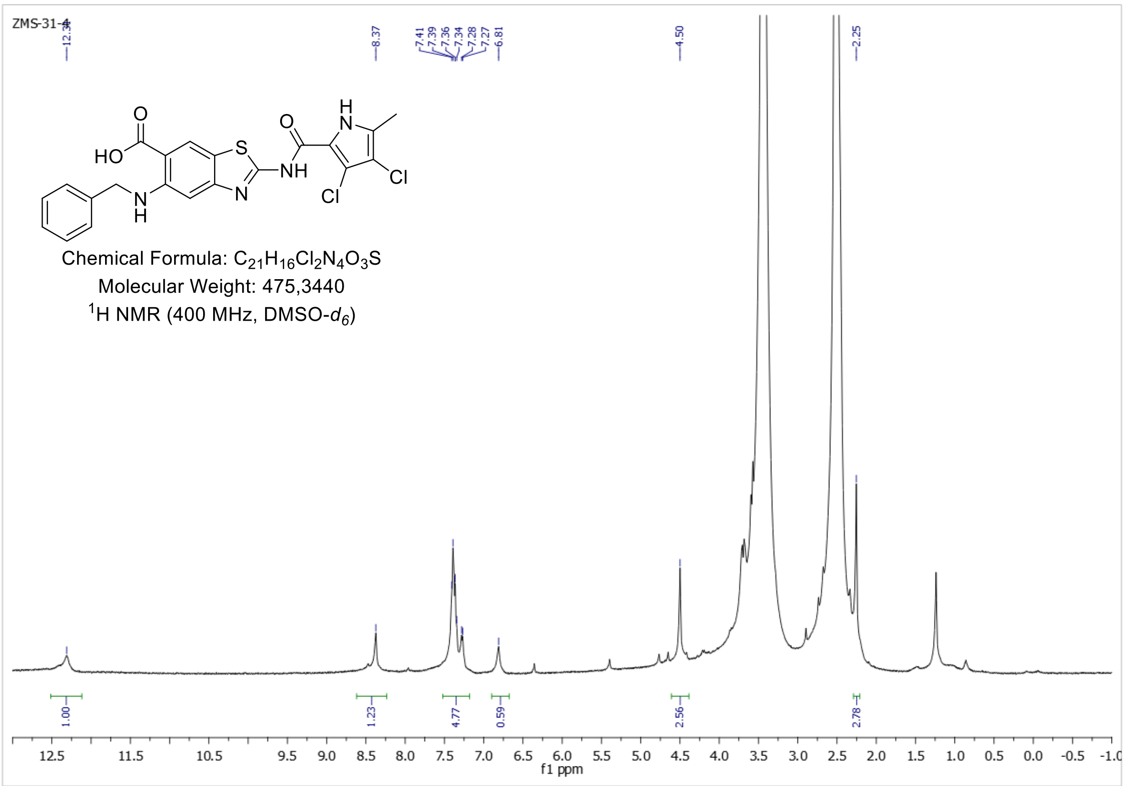

Compound **D**, HPLC trace:

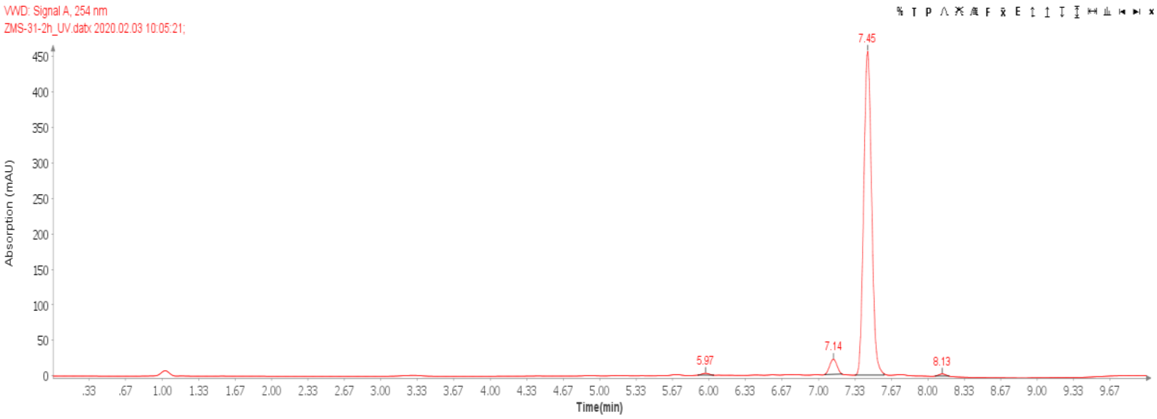

Peak	t_r (min)	Area	Area %
**1**	5.97	11.49	0.5
**2**	7.14	97.87	4.0
**D**	7.45	2300.39	95.0
**3**	8.13	12.28	0.5

Compound **14**, ^{1}H NMR:

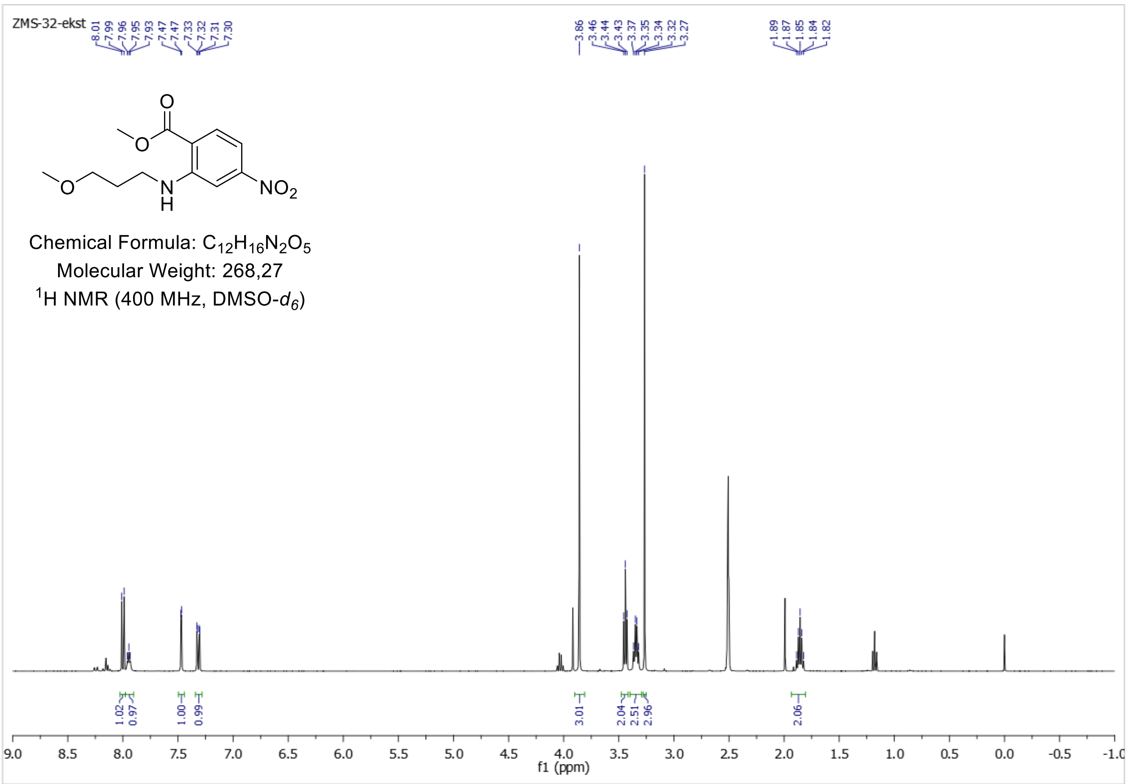

*Traces of ethyl acetate at 1.99 ppm, 4.03 ppm and 1.17 ppm.

Compound **16**, ^{1}H NMR:

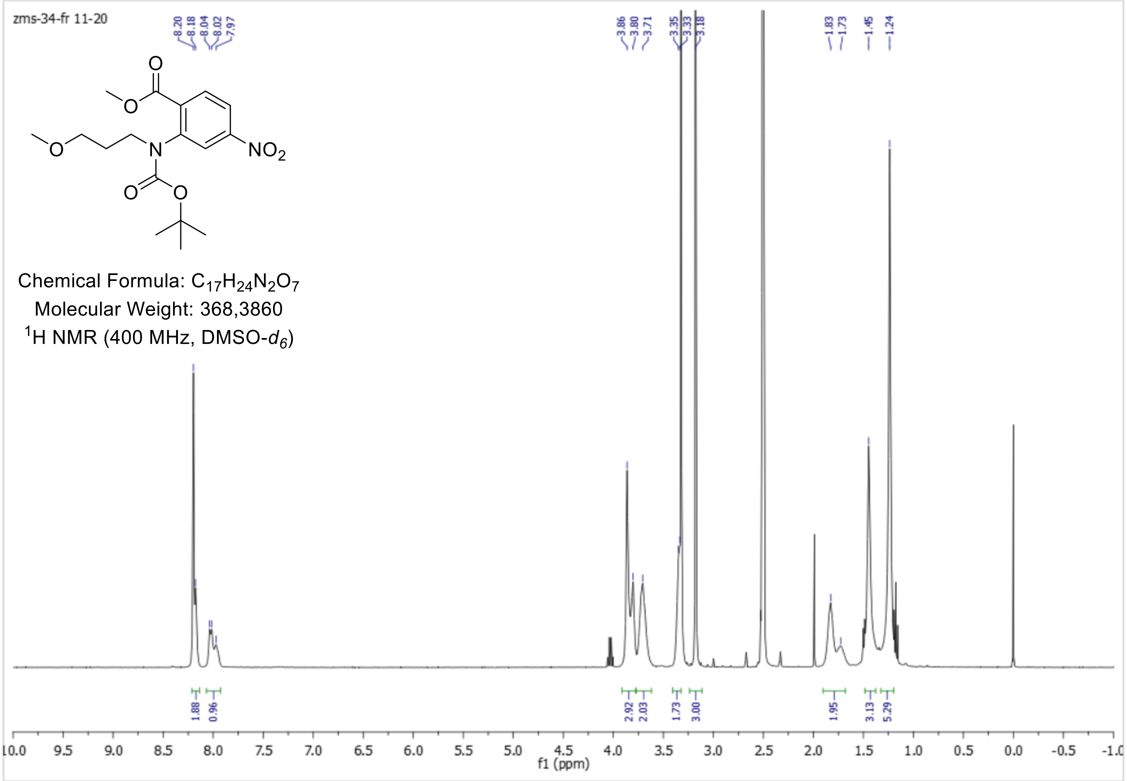

*Traces of ethyl acetate at 1.99 ppm, 4.03 ppm and 1.17 ppm.

Compound **1g**, ^{1}H NMR:

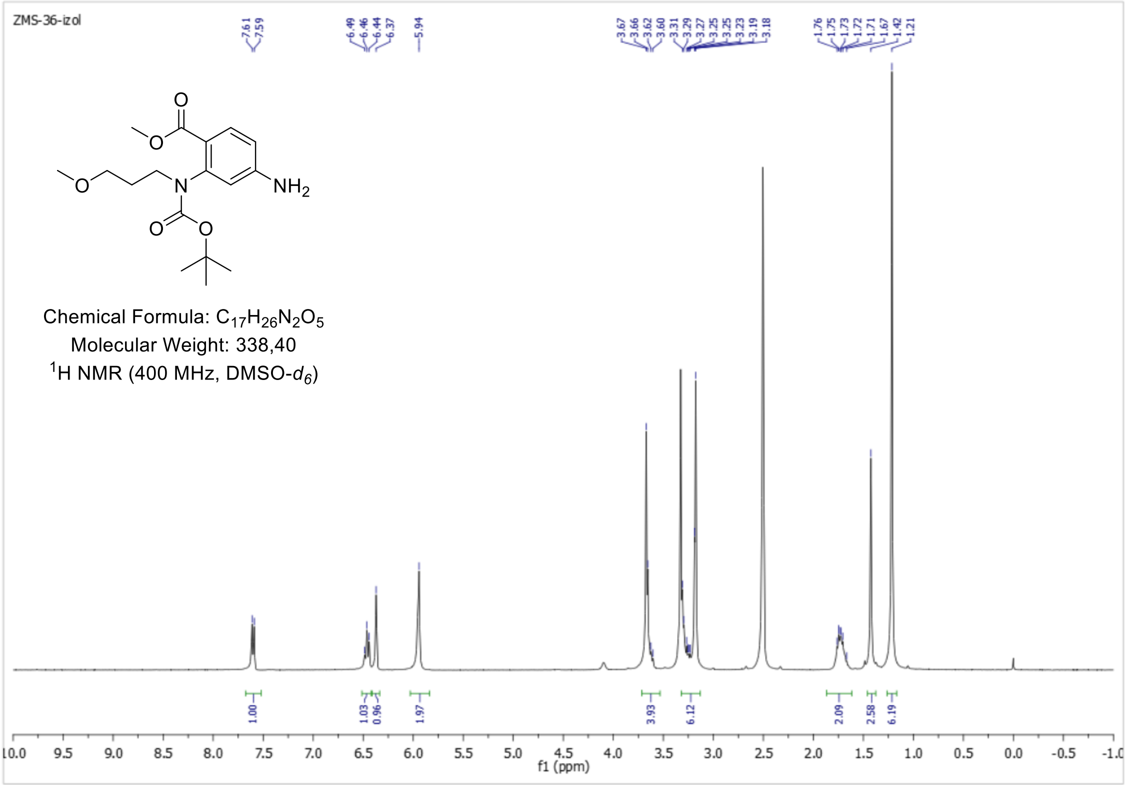

Compound **2g**, ^{1}H NMR:

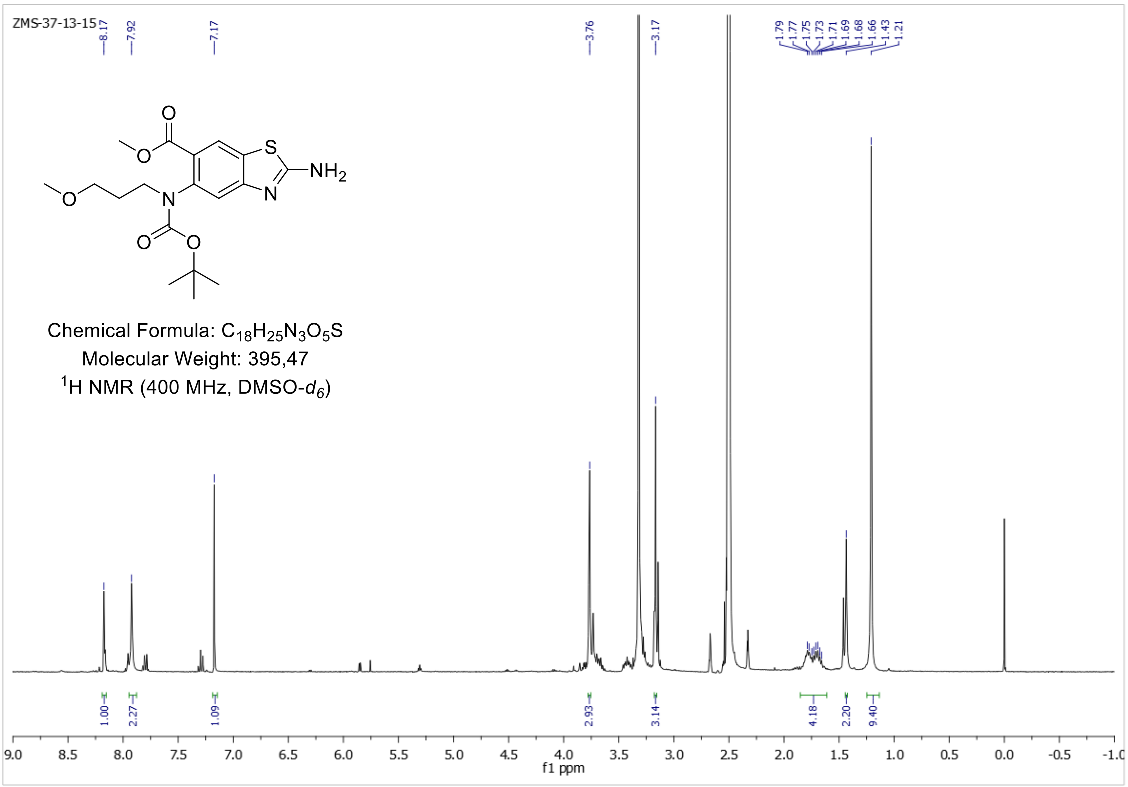

Compound **18**, ^{1}H NMR:

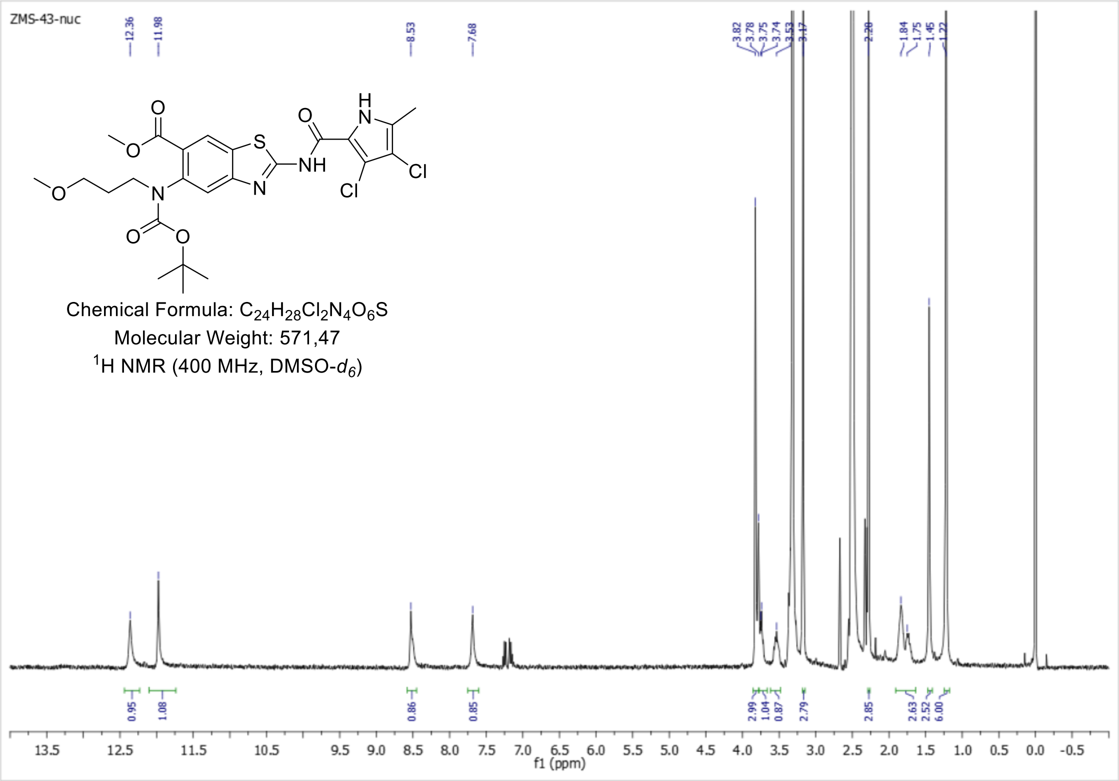

*Traces of toluene at 2.30 ppm, 7.18 ppm and 7.25 ppm.

Compound **20**, ^{1}H NMR:

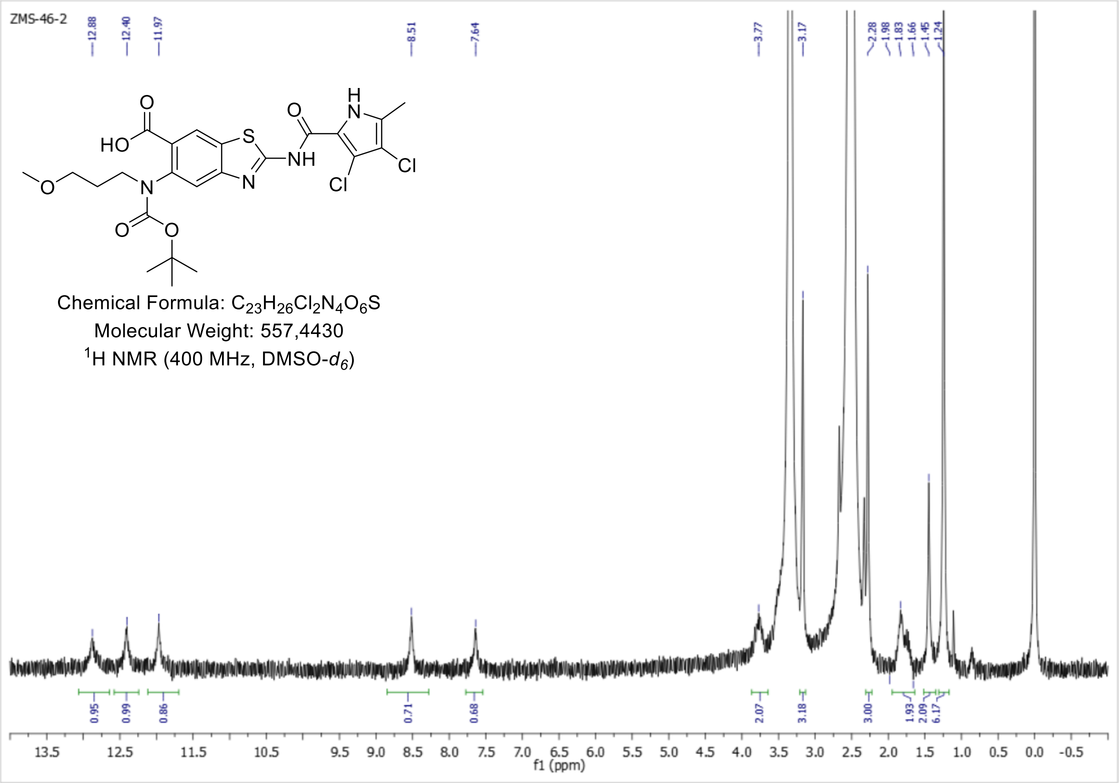

Compound **E**, ^{1}H NMR:

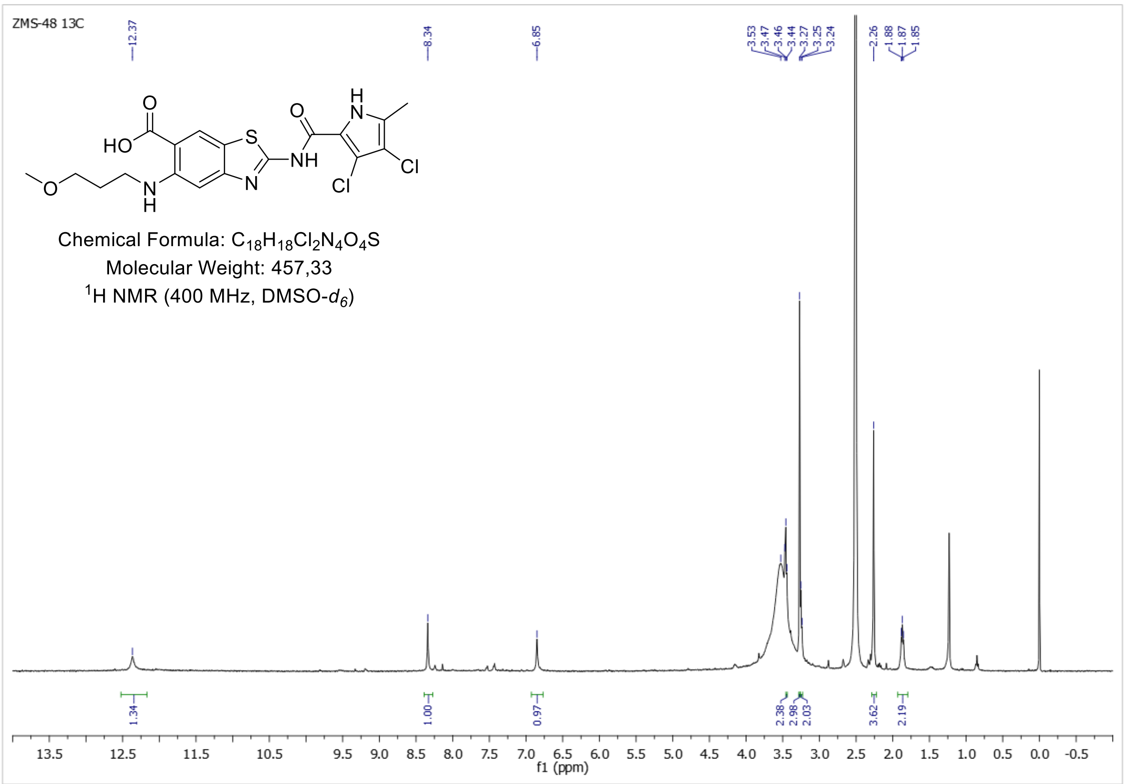

Compound **E**, ^{13}C NMR:

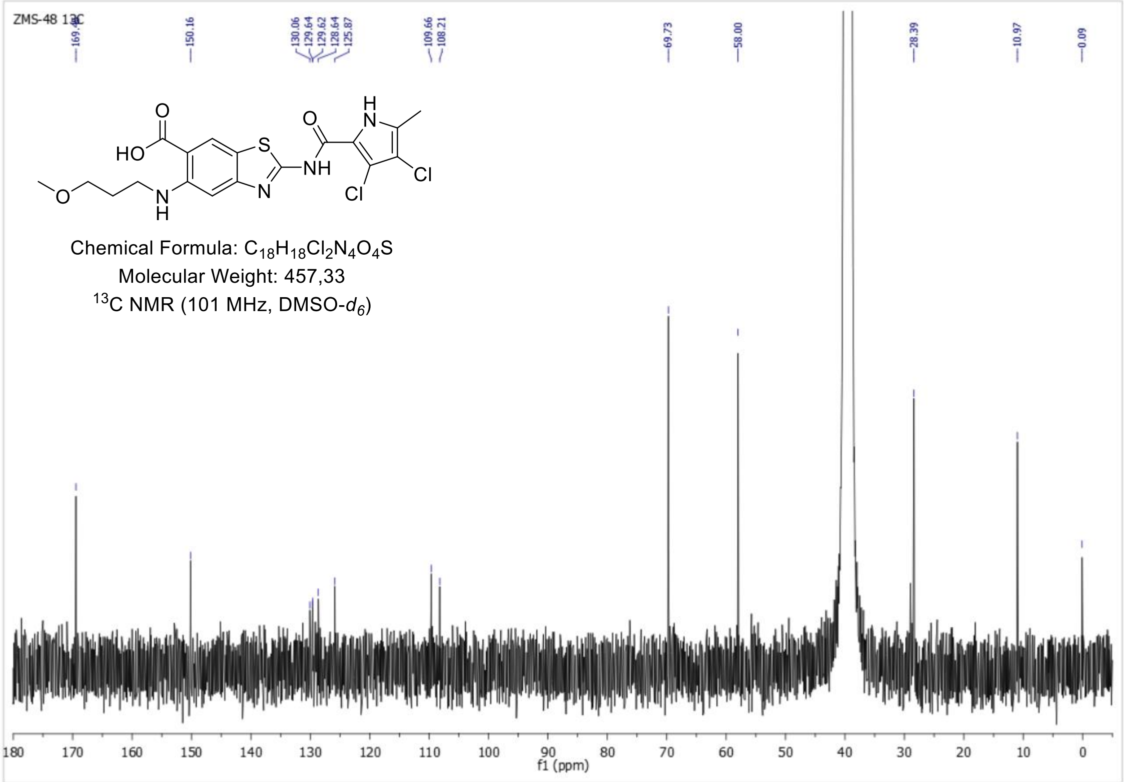

Compound **E**, HPLC trace:

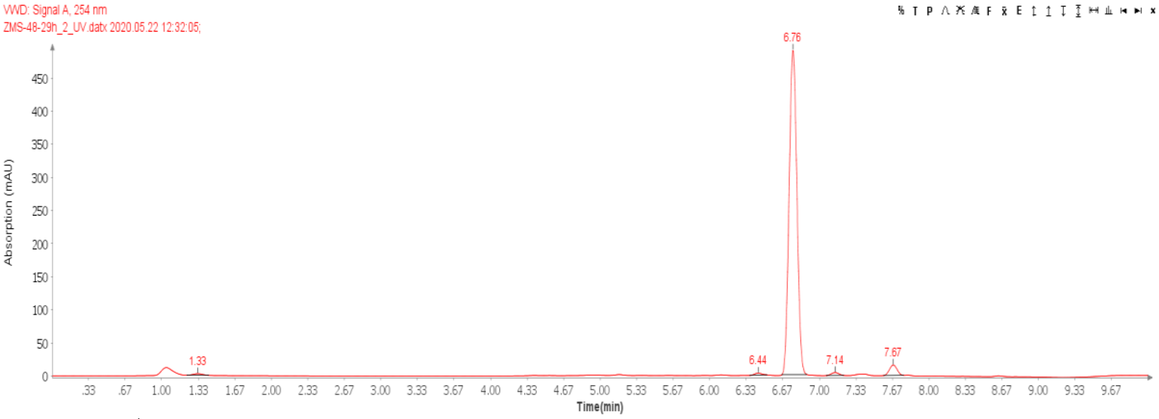

Peak	t_r (min)	Area	Area %
**1**	1.33	13.75	0.5
**2**	6.44	13.91	0.5
**E**	6.76	2426.93	95.1
**3**	7.14	20.84	0.8
**4**	7.68	75.53	3.0

Enzyme inhibition

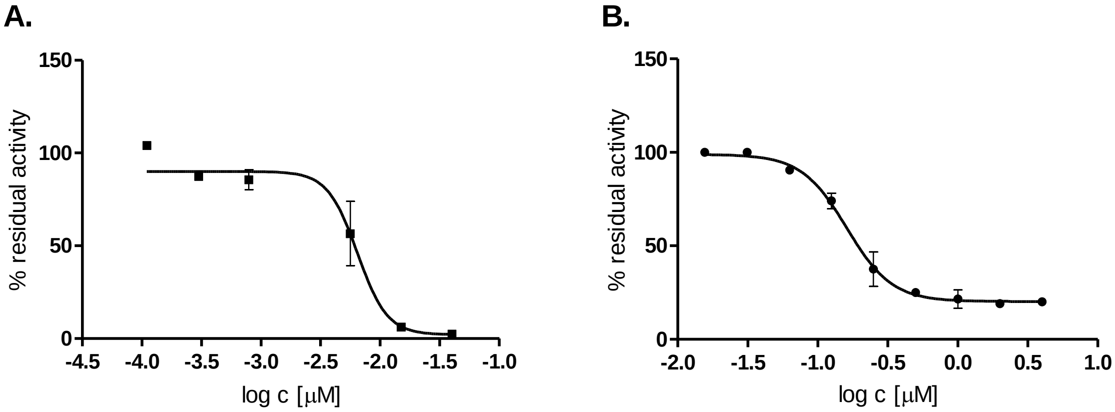

Figure S5. Dose-response curve for **E** against *E. coli* DNA gyrase (A.) and topo IV (B.) in supercoiling (for DNA gyrase) and relaxation (for topo IV) HTS assay, shown for an independent measurement in triplicate. The IC_{50} (mean ±SD) is the result of three independent measurements.

X-ray Data Collection and Refinement Statistics

Table S1. X-ray data collection and refinement statistics for *E. coli* GyrB24-LSJ38 complex

Data collection	
Beamline	I03 Diamond
Wavelength (Å)	0.9763
Detector	Pilatus3 6M
Resolution range (Å)	34.50 – 1.16 (1.18 – 1.16)
Space Group	*C*2
Cell parameters (Å)	a = 76.6, b = 50.3, c = 52.7, β = 102.4°
Total no. of measured intensities	426188 (17221)
Unique reflections	67664 (3328)
Multiplicity	6.3 (5.2)
Mean I/[illegible](I)	10.2 (1.4)
Completeness (%)	100.0 (100.0)
R_{merge}[a]	0.070 (1.128)
R_{meas}[b]	0.076 (1.261)
$CC_{½}$[c]	0.999 (0.490)
Wilson B value (Å^2)	11.5
Refinement	
Resolution range (Å)	33.94 – 1.16 (1.19 – 1.16)
Reflections: working/free[d]	64328/3335
R_{work}/ R_{free}[e]	0.142/0.170 (0.256/0.260)
Ramachandran plot: favoured/allowed/disallowed[f] (%)	98.4/1.1/0.5
R.m.s. bond distance deviation (Å)	0.011
R.m.s. bond angle deviation (º)	1.63
RSCC (inhibitor)[g]	0.99
Protein residues (ranges)	192 (9 – 98, 117 – 218)
No. of water/inhibitor	221/1
Mean B factors: protein/waters/inhibitor (Å^2)	18/34/12
Accession code	7P2N

Values in parentheses are for the outer resolution shell.

a Rmerge = $\sum_{hkl} \sum_i |I_i(hkl) - \langle I(hkl)\rangle| / \sum_{hkl} \sum_i I_i(hkl)$.

b Rmeas = $\sum_{hkl} [N/(N-1)]1/2 \times \sum_i |I_i(hkl) - \langle I(hkl)\rangle| / \sum_{hkl} \sum_i I_i(hkl)$, where Ii(hkl) is the ith observation of reflection hkl, ⟨I(hkl)⟩ is the weighted average intensity for all observations i of reflection hkl and N is the number of observations of reflection hkl.

[c] $CC_{½}$ is the correlation coefficient between symmetry equivalent intensities from random halves of the dataset.

[d] The data set was split into "working" and "free" sets consisting of 95 and 5% of the data respectively. The free set was not used for refinement.

e The R-factors Rwork and Rfree are calculated as follows: $R = \sum(| F_{obs} - F_{calc} |)/\sum| F_{obs} |$, where Fobs and Fcalc are the observed and calculated structure factor amplitudes, respectively.

[f] As calculated using MolProbity.

[g] Real Space Correlation Coefficient as calculated by the PDB validation server.

Experimental section

General chemistry information

Chemicals were purchased from Acros Organics (Geel, Belgium), Enamine Ltd. (Kyiv, Ukraine), Sigma-Aldrich (St.Louis, MO, USA), TCI (Tokyo, Japan), Fluorochem Ltd. (Derbyshire UK), and Apollo Scientific (Stockport, UK) and were used without additional purification. Air or moisture-sensitive reactions were carried out under an argon atmosphere. Analytical TLC was performed on silica gel Merck 60 F254 plates (0.25 mm), using visualization with UV light and spray reagents. Flash column chromatography was carried out on silica gel 60 (particle size 240-400 mesh). ^{1}H NMR (400 MHz, internal Me_4Si = 0 ppm), ^{13}C NMR (101 MHz, internal $CDCl_3$ = 77.16 ppm or DMSO-d_6 = 39.52 ppm) and ^{19}F NMR (376 MHz, external CCl_3F = 0 ppm) spectra were recorded on a Bruker AVANCE III 400 spectrometer (Bruker Corporation, Billerica, MA, USA) in DMSO-d_6 or $CDCl_3$ solution. HPLC-MS analyses were performed on an Agilent Technologies 1260 Infinity II LC System (Agilent Technologies, Inc., Santa Clara, CA, USA) coupled to an ADVION expression CMSL quadropole mass spectrometer (Advion Inc., Ithaca, USA). The column used was Waters XBridge C_{18} column (3.5 μm, 4.6 mm × 150 mm), with a flow rate of 1.5 mL/min, and sample injection volume of 10 μL. The mobile phase consisted of acetonitrile (solvent A) and 0.1% formic acid and 1% acetonitrile in ultrapure water (solvent B). The gradient (for solvent A) was 0–1 min, 25%; 1–6 min, 25–98%; 6–6.5 min, 98%; 6.5–7.5 min, 98–25%; 7.5–10.5 min, 25%. High resolution mass spectra were obtained using Exactive Plus Orbitrap mass spectrometer (Thermo Fisher Scientific, Waltham, Massachusetts, USA).

Synthetic procedures and analytical data

Methyl 2-Amino-5-fluorobenzo[*d*]thiazole-6-carboxylate (2c). To a yellow suspension of bromodimethylsulfonium bromide (0.39 g, 1.77 mmol, 1.5 eq) and sodium thiocyanate (0,34 g, 3.55 mmol, 3 eq) in anhydrous acetonitrile (12 mL) methyl 4-amino-2-fluorobenzoate (0.2 g, 1.2 mmol, 1 eq) was added in one portion. The reaction mixture was stirred at 80 °C overnight. The reaction mixture was quenched with saturated solution of $NaHCO_3$ (aq), the solid residue removed by filtration and washed with ethyl acetate. The organic layer was washed with water (20 mL), brine (20 mL) and dried over anhydrous Na_2SO_4. The crude product was further purified by flash column chromatography, eluent ethyl acetate/hexane 1:4 and reverse phase chromatography, eluent acetonitrile/water + 0.1% trifluoroacetic acid, gradient: 10% acetonitrile to 100% acetonitrile to obtain pure product as a white powder (13 mg, 5% yield). ^{1}H NMR (400 MHz, DMSO-d_6) δ 8.22 (d, *J* = 7.3 Hz, 1H), 8.16 (s, 2H), 7.19 (d, *J* = 12.6 Hz, 1H), 3.82 (s, 3H). ^{19}F NMR (376 MHz, DMSO-d_6) δ −113.59 (dd, *J* = 12.6, 7.6 Hz). ^{13}C NMR (101 MHz, DMSO-d_6) δ 171.74, 163.98 (d, *J* = 4.1 Hz), 158.23 (d, *J* = 36.9 Hz), 157.73 (d, *J* = 13.2 Hz), 126.58, 124.00, 110.20 (d, *J* = 11.8 Hz), 104.76 (d, *J* = 25.3 Hz), 51.99. MS (ESI$^+$) m/z: 227.1 ([M + H]$^+$). HRMS (ESI) m/z: [M – H]$^+$ Calcd for $C_9H_8O_2N_2FS$ 227.0285; found 227.0281 (– 1.64 ppm).

Methyl 2-Amino-5-((cyclohexylmethyl)amino)benzo[*d*]thiazole-6-carboxylate (2e). To a yellow suspension of bromodimethylsulfonium bromide (0.46 g, 2.1 mmol, 1.5 eq) and sodium thiocyanate (0.33 g, 4.14 mmol, 3 eq) in anhydrous acetonitrile (7 mL, 5 mL/mmol) methyl 4-amino-2-((*tert*-butyloxycarbonyl)(cyclohexylmethyl)amino)benzoate (0.5 g, 1.38 mmol, 1 eq) was added in one portion. The reaction mixture was stirred at 80 °C overnight. The reaction mixture was quenched with saturated solution of $NaHCO_3$ (aq) (10 mL), the solid residue removed by filtration and washed with ethyl acetate (10 mL). The organic layer was separated, washed with water (10 mL), brine (10 mL) and dried over anhydrous Na_2SO_4. The crude product was further purified by flash column chromatography, eluent dichloromethane/methanol 40:1 to obtain the title compound as a dark orange oil. (62 mg, 14% yield). ^{1}H NMR (400 MHz, DMSO-d_6) δ 8.02 (s, 1H), 7.81 (s, 2H), 7.72 (dd, *J* = 9.2, 3.9 Hz, 1H), 6.57 (s, 1H), 3.76 (s, 3H), 3.00 (t, J = 5.9 Hz, 2H), 1.85–1.51 (m, 6H), 1.18 (ddd, *J* = 19.7, 16.4, 10.8 Hz, 3H), 0.99 (dt, *J* = 12.0, 7.3 Hz, 2H). ^{13}C NMR (101 MHz, DMSO-d_6) δ 170.53, 168.00, 159.34, 150.52, 123.46, 117.59, 103.79, 98.88, 51.29, 48.87, 36.78, 30.67, 26.09, 25.50. MS (ESI$^+$) m/z: 319.9 ([M + H]$^+$). HRMS (ESI) m/z: [M – H]$^+$ Calcd for $C_{16}H_{22}O_2N_3S$ 320.1427; Found 320.1422 (– 1.57 ppm).

2-Amino-5-fluoro-4-methylbenzo[*d*]thiazole-6-carbonitrile (2a). KSCN (2.6 g, 26.6 mmol, 4 eq) was dissolved in acetic acid (40 mL) and after 20 minutes Br_2 (0.68 mL, 13.3 mmol, 2 eq) was added dropwise. After stirring at 22 °C for 30 minutes, the resulting suspension was added dropwise to the solution of 4-amino-2-fluoro-3-methylbenzonitrile (1.0 g, 6.6 mmol, 1 eq) in acetic acid (30 mL). The resulting mixture was stirred overnight at 22 °C and neutralised with 4 M NaOH (30 mL) to pH 7–8 at 0 °C. The precipitate was collected, washed with water and air-dried. The precipitate was percolated with methanol, concentrated and washed with ethyl acetate to get a title compound as a yellow solid (0.50 g, 33% yield). ^{1}H NMR (400 MHz, DMSO-d_6) δ 8.27 (s, 2H), 8.05 (d, *J* = 6.4 Hz, 1H), 2.35 (d, *J* = 1.9 Hz, 3H). ^{19}F NMR (376 MHz, DMSO-d_6) δ (−117.82)–(−117.87) (m). MS (ESI$^+$) m/z: 207.9 ([M + H]$^+$). HPLC purity: 99%.

2-Amino-5-fluoro-4-methylbenzo[*d*]thiazole-6-carboxylic Acid (7). To a suspension of **2a** (0.45 g, 2.17 mmol, 1 eq) in water (5 mL), were sequentially added H_2SO_4 (5 mL) and acetic acid (5 mL) at 0 °C. The resulting mixture was stirred overnight at 100 °C. Ice-cold water (15 mL) was added and the obtained precipitate was collected and washed with water

to get the title compound as a white solid (0.4 g, 83% yield). ^{1}H NMR (400 MHz, DMSO-d_6) δ 8.01 (d, *J* = 2.0 Hz, 1H), 8.00 (s, 1H), 2.33 (d, *J* = 2.0 Hz, 3H). ^{19}F NMR (376 MHz, DMSO-d_6) δ −118.26 (d, *J* = 4.6 Hz). MS (ESI$^+$) m/z: 268.1 ([M + CH_3CN + H]$^+$).

4-Methoxybenzyl 2-Amino-5-fluoro-4-methylbenzo[*d*]thiazole-6-carboxylate (8). To a solution of **7** (0.40 g, 1.77 mmol, 1 eq) in dry dimethyl formamide (10 mL), K_2CO_3 (2.6 mmol, 1.5 eq) and 4-methoxybenzyl chloride (0.25 mL, 1.86 mmol, 1 eq) were added. The reaction mixture was stirred overnight at 40 °C. The white precipitate was filtered off and the filtrate was concentrated. The crude solid was purified by flash column chromatography eluent dichloromethane/methanol 20:1 to get the title compound as a light yellow solid (0.2 g, 37% yield). ^{1}H NMR (400 MHz, DMSO-d_6) δ 8.07 (s, 2H), 8.04 (d, *J* = 7.1 Hz, 1H), 7.40 (d, *J* = 8.7 Hz, 2H), 6.96 (d, *J* = 8.7 Hz, 2H), 5.24 (s, 2H), 3.76 (s, 3H), 2.33 (d, *J* = 2.0 Hz, 3H).

4-Methoxybenzyl 2-(3,4-Dichloro-5-methyl-1*H*-pyrrole-2-carboxamido)-5-fluoro-4-methylbenzo[*d*]thiazole-6-carboxylate (9). To a solution of the above 2-amino benzothiazole **8** (0.22 g, 0.65 mmol, 1 eq) in dry dimethyl formamide (5 mL) Na_2SO_4 (68.6 mg, 0.65 mmol, 1 eq) and 2-trichloroacetyl-3,4-dichloro-5-methyl-1*H*-pyrrole (0.34 g, 1.16 mmol, 1.8 eq) were added. The resulting mixture was stirred at 60 °C overnight. The reaction mixture was concentrated and resuspended in 10% citric acid (5 mL). The precipitate was collected, washed with water (5 mL), ethyl acetate and with acetone to get the title compound as a dark grey solid (0.10 g, 21% yield). ^{1}H NMR (400 MHz, DMSO-d_6) δ 12.36 (s, 1H), 12.09 (s, 1H), 8.40 (s, 1H), 7.43 (d, *J* = 8.6 Hz, 2H), 6.97 (d, *J* = 8.6 Hz, 2H), 5.29 (s, 2H), 3.77 (s, 3H), 2.27 (s, 3H). (*water covers one signal for pyrrole CH_3 group). ^{19}F NMR (376 MHz, DMSO-d$_6$) δ −118.21 (s). MS (ESI$^-$) m/z: 521.2 ([M − H]$^-$). HPLC purity: 99%.

2-(3,4-Dichloro-5-methyl-1*H*-pyrrole-2-carboxamido)-5-fluoro-4-methylbenzo[*d*]thiazole-6-carboxylic Acid (B). **9** (73.0 mg, 0.14 mmol, 1 eq) was suspended in 1 M HCl in acetic acid (0.7 mL, 5 mL/mmol) and 4 M HCl in 1,4-dioxane (0.7 mL, 5 mL/mmol) and stirred for 30 h at 50 °C, then concentrated and washed with diethyl ether to obtain the title compound as a grey solid (0.05 g, 87% yield). ^{1}H NMR (400 MHz, DMSO-d_6) δ 12.76 (s, 1H), 12.25 (s, 1H), 8.44 (d, *J* = 6.8 Hz, 1H), 3.63 (s, 3H), 2.34 (s, 3H). ^{19}F NMR (376 MHz, DMSO-d_6) δ −118.39 (s). ^{13}C NMR (101 MHz, DMSO-d_6) δ 165.14, 159.31, 156.82, 130.07, 126.32, 122.41, 117.39, 116.67, 115.99, 115.12, 114.99, 110.05, 66.36, 11.04, 10.20. MS (ESI$^-$) m/z: 401.3 ([M − H]$^-$). HRMS (ESI) m/z: [M − H]$^-$ Calcd for $C_{15}H_9O_3N_3Cl_2FS$ 399.9731; Found 399.9729 (− 0.65 ppm). HPLC purity: 98%.

Methyl 2-Fluoro-4-nitrobenzoate (10).[25] To a solution of 2-fluoro-4-nitrobenzoic acid (2.0 g, 10.8 mmol, 1 eq) in methanol (50 mL) H_2SO_4 (1.15 mL, 21.6 mL, 2 eq) was added. The reaction mixture was stirred overnight at 65 °C and concentrated. To the solid residue saturated $NaHCO_3$(aq) (35 mL) and ethyl acetate (50 mL) were added. The organic phase was washed with water (50 mL) brine (2 x 50 mL), dried over Na_2SO_4 and concentrated to obtain the title compound as an orange solid (2.0 g, 93% yield). ^{1}H NMR (400 MHz, DMSO-d_6) δ 8.30–8.22 (m, 1H), 8.16 (qd, *J* = 8.6, 4.2 Hz, 2H), 3.92 (s, 3H).

Methyl 2-(Benzylamino)-4-nitrobenzoate (11). To a solution of **10** (4.0 g, 20.1 mmol, 1 eq) in acetonitrile (100 mL, 5 mL/mmol) benzylamine (2.63 mL, 24.10 mmol, 1.2 eq) and K_2CO_3 (5.5 g, 40.17 mmol, 2 eq) were added. The reaction mixture was stirred overnight at 60 °C and concentrated. To the solid residue was partitioned between 1 M HCl(aq) (100 mL) and ethyl acetate (100 mL). The organic layer was washed with water (100 mL) and brine (2 x 100 mL), dried over Na_2SO_4 and concentrated to obtain the crude compound as an orange solid. The crude product was purified by recrystallization from hexane to obtain the title compound as orange crystals (4.5 g, 79%). ^{1}H NMR (400 MHz, DMSO-d_6) δ 8.37 (t, *J* = 5.7 Hz, 1H), 8.05 (d, *J* = 8.7 Hz, 1H), 7.48–7.21 (m, 7H), 4.59 (d, *J* = 5.8 Hz, 2H), 3.88 (s, 3H).

Methyl 2-(*N*-Benzylacetamido)-4-nitrobenzoate (12). To a solution of **11** (4.5 g, 15.7 mmol, 1 eq) and 4-DMAP (0.19 g, 1.57 mmol, 0.1 eq) in dry dichloromethane (80 mL, 5 mL/mmol), DIEA (5.4 mL, 31.4 mmol, 2 eq) was added. After stirring for 10 minutes at 22 °C acetyl chloride (11.2 mL, 157.2 mmol, 10 eq) was added. The reaction mixture was stirred overnight at 35 °C and concentrated. The residue was partitioned between 1 M HCl (100 mL) and dichloromethane (100 mL). The combined organic layers were washed with brine, dried over Na_2SO_4, filtered and concentrated. The crude product was purified by flash column chromatography, eluent ethyl acetate/hexane 1:1 to get the title compound as an orange solid (4.4 g, 85%). ^{1}H NMR (400 MHz, DMSO-d$_6$) δ 8.30 (dd, *J* = 8.6, 2.3 Hz, 1H), 8.06 (d, *J* = 8.6 Hz, 1H), 8.00 (d, *J* = 2.3 Hz, 1H), 7.26 (qd, *J* = 5.6, 2.7 Hz, 3H), 7.13 (dd, *J* = 7.5, 1.8 Hz, 2H), 4.94 (d, *J* = 14.6 Hz, 1H), 4.56 (d, *J* = 14.6 Hz, 1H), 3.72 (s, 3H), 1.77 (s, 3H).

Methyl 4-Amino-2-(*N*-benzylacetamido)benzoate (1f). To a solution of **12** (4.3 g, 13.1 mmol, 1 eq) in acetic acid (65 mL, 5 mL/mmol) iron powder (7.3 g, 131 mmol, 10 eq) was added and the resulting suspension vigorously stirred for 2 h. Water (20 mL) and methanol (20 mL) were added, the resulting solution was filtered through cotton and concentrated under reduced pressure. The oily residue was partitioned between water (100 mL) and ethyl acetate (3 x 100 mL). The combined organic layers were washed with saturated $NaHCO_3$(aq) (100 mL), brine (100 mL), dried over Na_2SO_4, filtered

and concentrated to obtain the title compound as a brown oil (3.2 g, 81%). ^{1}H NMR (400 MHz, DMSO-d_6) δ 7.70 (d, *J* = 8.6 Hz, 1H), 7.35–7.11 (m, 5H), 6.53 (dd, *J* = 8.7, 2.3 Hz, 1H), 6.15 (d, *J* = 2.3 Hz, 1H), 6.09 (s, 2H), 5.20 (d, *J* = 15.0 Hz, 1H), 4.01 (d, *J* = 15.0 Hz, 1H), 3.66 (s, 3H), 1.71 (s, 3H). MS (ESI$^+$) m/z: 299.1 ([M + H]$^+$).

Methyl 2-Amino-5-(*N*-benzylacetamido)benzo[*d*]thiazole-6-carboxylate (2f). KSCN (4.1 g, 42.2 mmol, 4 eq) was dissolved in acetic acid (70 mL) and Br_2 (1.1 mL, 21.1 mmol, 2 eq) was added dropwise. After stirring at 22 °C for 30 minutes, the resulting suspension was added dropwise to the solution of the above aniline **1f** (3.15 g, 10.5 mmol, 1 eq) in acetic acid (45 mL). The resulting mixture was stirred overnight at 22 °C and neutralised with 4 M NaOH (500 mL) to pH 7–8 at 0 °C. The precipitate was collected, washed with water and air-dried. The resulting solid was percolated with methanol to obtain the crude product which was further purified by flash column chromatography, eluent dichloromethane/methanol 20:1 to get the title compound as a light brown solid (0.315 g, 8.4%). ^{1}H NMR (400 MHz, DMSO-d_6) δ 8.06 (s, 1H), 7.27 (ddd, *J* = 8.6, 6.4, 3.5 Hz, 3H), 7.21–7.15 (m, 2H), 6.85 (s, 2H), 6.46 (s, 1H), 5.16 (d, *J* = 15.0 Hz, 1H), 4.07 (d, *J* = 15.0 Hz, 1H), 3.70 (s, 3H), 1.74 (s, 3H). MS (ESI$^+$) m/z: 356.1 ([M + H]$^+$). HPLC purity: 98%.

Methyl 5-(*N*-Benzylacetamido)-2-(3,4-dichloro-5-methyl-1*H*-pyrrole-2-carboxamido)benzo[*d*]thiazole-6-carboxylate (13). To a solution of above 2-amino benzothiazole **2f** (0.312 g, 0.88 mmol, 1 eq) in dry dimethyl formamide (4.4 mL, 5 mL/mmol) Na_2SO_4 (0.093 g, 0.88 mmol, 1 eq) and 2-trichloroacetyl-3,4-dichloro-5-methyl-1*H*-pyrrole (0.29 g, 0.96 mmol, 1.1 eq) were added. The resulting mixture was stirred at 60 °C overnight. The reaction mixture was concentrated, resuspended in 10% citric acid, the precipitate collected and washed with water. The crude product was further purified by flash column chromatography eluent dichloromethane/methanol 20:1 to obtain the title compound as a black solid (0.14 g, 29%). ^{1}H NMR (400 MHz, DMSO-d_6) δ 12.36 (s, 1H), 11.96 (s, 1H), 8.63 (s, 1H), 7.37–6.99 (m, 6H), 5.22 (d, *J* = 14.6 Hz, 1H), 4.26 (d, *J* = 14.8 Hz, 1H), 3.76 (s, 3H), 2.27 (s, 3H), 1.76 (s, 3H). MS (ESI$^-$) m/z: 529.7 ([M – H]$^-$).

5-(*N*-Benzylacetamido)-2-(3,4-dichloro-5-methyl-1*H*-pyrrole-2-carboxamido)benzo[*d*]thiazole-6-carboxylic Acid (C). To a suspension of **13** (0.134 g, 0.25 mmol, 1 eq) in methanol (5 mL) 2 M NaOH (1.3 mL, 2.53 mmol, 10 eq) was added and stirred at 60 °C, monitored by LC-MS. The reaction mixture was neutralised with 4 M HCl (0.6 mL). The precipitate was collected and triturated with cold water and few drops of methanol to obtain title compound as a brown solid (0.05 g, 38%). ^{1}H NMR (400 MHz, DMSO-d_6) δ 13.15 (s, 1H), 12.43 (s, 1H), 11.97 (s, 1H), 8.66 (s, 1H), 7.50–6.95 (m, 6H), 5.51 (d, *J* = 14.8 Hz, 1H), 3.99 (d, *J* = 14.8 Hz, 1H), 2.27 (s, 3H), 1.76 (s, 3H). ^{13}C NMR (101 MHz, DMSO-d_6) δ 169.35, 166.33, 140.52, 137.63, 130.32, 128.62, 128.24, 127.14, 126.02, 124.00, 115.42, 109.96, 52.05, 22.50, 11.04. MS (ESI$^-$) m/z: 514.6 ([M – H]$^-$). HRMS (ESI) m/z: [M – H]$^-$ Calcd for $C_{23}H_{17}O_4N_4Cl_2S$ 515.0352; Found 515.0349 (–0.73 ppm). HPLC purity: 96%.

Methyl 2-(Benzyl(*tert*-butoxycarbonyl)amino)-4-nitrobenzoate (15). To the solution of **11** (0.64 g, 2.23 mmol, 1 eq) in tetrahydrofuran (23 mL) di-tert-butyl dicarbonate (4.9 g, 22.35 mmol, 10 eq) and 4-DMAP (54 mg, 0.45 mmol, 0.2 eq) were added and stirred at 80 °C for 24 h 7 days, then the reaction mixture was concentrated. The solid residue was partitioned between 1 M HCl (50 mL) and ethyl acetate (50 mL), the organic layer was washed with water (50 mL), brine (2 x 50 mL), dried over Na_2SO_4 and concentrated. The crude product was purified by flash column chromatography, eluent ethyl acetate/hexane 1:4 to get the title compound as a light yellow solid (0.3 g, 35%). ^{1}H NMR (400 MHz, DMSO-d_6) δ 8.15 (dd, *J* = 8.6, 2.2 Hz, 1H), 8.05–7.81 (m, *J* = 54.9 Hz, 2H), 7.29 (dd, *J* = 11.2, 7.2 Hz, 5H), 5.11–4.54 (m, *J* = 82.9 Hz, 2H), 3.80 (s, 3H), 1.38 (s, 3H), 1.28 (s, 6H).

Methyl 4-Amino-2-(benzyl(*tert*-butoxycarbonyl)amino)benzoate (1d). To a solution of **15** (0.30 g, 0.77 mmol, 1 eq) in acetic acid (7.7 mL) iron powder (0.43 g, 7,7 mmol, 10 eq) was added and the resulting suspension vigorously stirred at 22 °C for 2 h. Water (20 mL) and methanol (20 mL) were added, the resulting suspension filtered through cotton and concentrated under reduced pressure. The oily residue was partitioned between water (50 mL) and ethyl acetate (50 mL). The combined organic layers were washed with saturated $NaHCO_3$ (aq) (50 mL), brine (50 mL), dried over Na_2SO_4, filtered and concentrated (0.2 g, 76%). The product was used in the next synthetic step without further purification. MS (ESI$^+$) m/z: 357.0 ([M + H]$^+$). HPLC purity: 100%.

Methyl 2-Amino-5-(benzyl(*tert*-butoxycarbonyl)amino)benzo[*d*]thiazole-6-carboxylate (2d). KSCN (0.20 g, 2.1 mmol, 4 eq) was dissolved in acetic acid (5 mL) and Br_2 (54 μL, 1.04 mmol, 2 eq) was added dropwise. After stirring at 22 °C for 30 minutes, the resulting suspension was added dropwise to the solution of **1d** (0.20 g, 0.52 mmol, 1 eq) in acetic acid (5 mL). The resulting mixture was stirred overnight at 22 °C and neutralised with 4 M NaOH (43 mL) to pH 7–8 at 0 °C. The precipitate was collected, washed with water and air-dried. The resulting solid was percolated with methanol, and the crude product which was further purified by flash column chromatography, eluent ethyl acetate/hexane 1:1 to get the title compound as a yellow solid (35 mg, 16%). ^{1}H NMR (400 MHz, DMSO-d_6) δ 8.23 (s, 1H), 7.93 (s, 2H), 7.41–7.27 (m, 6H), 5.18 (d, *J* = 15.3 Hz, 1H), 4.31 (d, *J* = 15.1 Hz, 1H), 3.81 (s, 3H), 1.47 (s, 3H), 1.31 (s, 6H). MS (ESI$^-$) m/z: 412.0 ([M – H]$^-$).

Methyl 5-(Benzyl(*tert*-butoxycarbonyl)amino)-2-(3,4-dichloro-5-methyl-1*H*-pyrrole-2-carboxamido)benzo[*d*]thiazole-6-carboxylate (17). A mixture of 3,4-dichloro-5-methyl-1*H*-pyrrole-2-carboxylic acid (30 mg, 0.16 mmol,1.2 eq) and $SOCl_2$ (0.4 mL, 2.5 mL/mmol) was stirred at 75 °C for 40 minutes. The volatiles were removed under reduced pressure, then the above 2-amino benzothiazole 2d (54 mg, 0.13 mmol, 1 eq) and toluene (3 mL) were added. The resulting mixture was stirred at 130 °C overnight. The precipitate was collected and triturated with toluene and methanol to get the title compound as a black solid (43 mg, 56%). ^{1}H NMR (400 MHz, DMSO-d_6) δ 12.34 (s, 1H), 11.89 (s, 1H), 8.53 (s, 1H), 7.38–7.22 (m, *J* = 26.5, 9.7 Hz, 6H), 5.18 (d, *J* = 14.9 Hz, 1H), 4.33 (d, *J* = 15.3 Hz, 1H), 3.79 (d, *J* = 12.5 Hz, 3H), 2.26 (d, *J* = 6.3 Hz, 3H), 1.43 (s, 3H), 1.26 (s, 6H). MS (ESI$^-$) m/z: 587.9 ([M – H]$^-$).

5-(Benzyl(*tert*-butoxycarbonyl)amino)-2-(3,4-dichloro-5-methyl-1*H*-pyrrole-2-carboxamido)benzo[*d*]thiazole-6-carboxylic Acid (19). To a suspension of 17 (43 mg, 0.07 mmol, 1 eq) in methanol (2 mL) 2 M NaOH (0.55 mL, 1.1 mmol, 15 eq) was added portionwise and stirred at 60 °C for 96 h. The reaction mixture was neutralised with 1 M HCl (1.1 mL). The obtained suspension was filtered off and the solid was washed with cold water and few drops of methanol and ethyl acetate to get the title compound as a brown solid (15 mg, 36%). ^{1}H NMR (400 MHz, DMSO-d_6) δ 12.94 (s, 1H), 12.34 (s, 1H), 11.84 (s, 1H), 8.53 (s, 1H), 7.40–7.26 (m, *J* = 8.5 Hz, 5H), 7.08 (s, 1H), 5.28 (d, *J* = 16.3 Hz, 1H), 4.21 (d, *J* = 15.7 Hz, 1H), 2.26 (s, 3H), 1.43 (s, 3H), 1.29 (s, 6H). MS (ESI$^-$) m/z: 574.0 ([M – H]$^-$).

5-(Benzylamino)-2-(3,4-dichloro-5-methyl-1*H*-pyrrole-2-carboxamido)benzo[*d*]thiazole-6-carboxylic Acid (D). The reaction was carried out under an argon atmosphere. **19** (15 mg, 0.026 mmol, 1 eq) was dissolved in 4 M HCl in 1,4-dioxane (0.13 mL) and stirred at 22 °C monitored by LC-MS, then concentrated to obtain the title compound as a brown solid (10 mg, 81%). ^{1}H NMR (400 MHz, DMSO-d_6) δ 12.31 (s, 1H), 8.37 (s, 1H), 7.59–7.20 (m, *J* = 40.9, 8.7 Hz, 5H), 6.81 (s, 1H), 4.50 (s, 2H), 2.25 (s, 3H). MS (ESI$^+$) m/z: 476.0 ([M + H]$^+$). HRMS (ESI) m/z: [M – H]$^+$ Calcd for $C_{21}H_{17}O_3N_4Cl_2S$ 475.0393; Found 475.0382 (– 2.34 ppm). HPLC purity: 95%.

Methyl 2-((3-Methoxypropyl)amino)-4-nitrobenzoate (14). To a solution of **10** (2.0 g, 10 mmol, 1 eq) in acetonitrile (50 mL) 3-methoxypropan-1-amine (1.23 mL, 12 mmol, 1.2 eq) and K_2CO_3 (2.77 g, 20.1 mmol, 2 eq) were added. The reaction mixture was stirred overnight at 60 °C and concentrated. The solid residue was partitioned between 1 M HCl (50 mL) and ethyl acetate (50 mL). The organic phase layer washed with water (50 mL), brine (2 x 50 mL), dried over Na_2SO_4 and concentrated to obtain the crude compound as an orange solid, containing 10% of the starting material by ^{1}H NMR (2.3 g, 86%). ^{1}H NMR (400 MHz, DMSO-d_6) δ 8.00 (d, *J* = 8.7 Hz, 1H), 7.95 (t, *J* = 5.2 Hz, 1H), 7.47 (d, *J* = 2.3 Hz, 1H), 7.32 (dd, *J* = 8.7, 2.3 Hz, 1H), 3.86 (s, 3H), 3.44 (t, *J* = 5.9 Hz, 2H), 3.34 (dd, *J* = 12.2, 6.7 Hz, 2H), 3.27 (s, 3H), 1.85 (p, *J* = 6.5 Hz, 2H). MS (ESI$^+$) m/z: 268.9 ([M + H]$^+$).

Methyl 2-((*tert*-Butoxycarbonyl)(3-methoxypropyl)amino)-4-nitrobenzoate (16). To a solution of **14** (8.6 mmol, 1 eq) in tetrahydrofuran (43 mL), di-*tert*-butyl dicarbonate (9.4 g, 43 mmol, 5 eq) and 4-DMAP (1.7 mmol, 0.2 eq) were added and stirred at 80 °C for 7 days. The reaction mixture was concentrated and the solid residue was partitioned between 1 M HCl (50 mL) and ethyl acetate (50 mL). The organic layer was washed with brine, dried over Na_2SO_4 and concentrated. The crude product was purified by flash column chromatography eluent ethyl acetate/hexane 1:2 to get the title compound as a yellow solid (3.0 g, 95%). ^{1}H NMR (400 MHz, DMSO-d_6) δ 8.26 – 8.14 (m, *J* = 8.6 Hz, 2H), 8.09–7.92 (m, 1H), 3.96–3.77 (m, 3H), 3.71 (s, 2H), 3.34 (d, *J* = 5.7 Hz, 2H), 3.18 (s, 3H), 1.87 – 1.69 (m, 2H), 1.45 (s, 3H), 1.24 (s, 6H).

Methyl 4-Amino-2-((*tert*-butoxycarbonyl)(3-methoxypropyl)amino)benzoate (1g). To a solution of **16** (3.0 g, 8.14 mmol,1 eq) in methanol (50 mL), Pd/C (0.3 g, 81.4 mmol, 10 m/m%) was added and stirred under H_2 atmosphere (1 atm) for 1.5 h at 22 °C. The reaction mixture was filtered through Celite and the mother liquid was concentrated to obtain the title compound as an off white solid (1.8 g, 65%). ^{1}H NMR (400 MHz, DMSO-d_6) δ 7.60 (d, *J* = 8.5 Hz, 1H), 6.46 (t, *J* = 9.0 Hz, 1H), 6.37 (s, 1H), 5.94 (s, 2H), 3.76–3.55 (m, 4H), 3.31–3.13 (m, 6H), 1.82–1.63 (m, *J* = 25.6, 11.4 Hz, 2H), 1.42 (s, 3H), 1.21 (s, 6H).

Methyl 2-Amino-5-((*tert*-butoxycarbonyl)(3-methoxypropyl)amino)benzo[*d*]thiazole-6-carboxylate (2g). KSCN (2.1 g, 21.3 mmol, 4 eq) was dissolved in acetic acid (20 mL) and Br_2 (0.55 mL, 10.6 mmol, 2 eq) was added dropwise. After stirring at 22 °C for 30 minutes, the resulting suspension was added dropwise to the solution of **1g** (1.8 g,5.32 mmol, 1 eq) in acetic acid (15 mL). The resulting mixture was stirred overnight at 22 °C and neutralised with 4 M NaOH (152 mL) to pH 7–8 at 0 °C. The precipitate was collected, washed with water and air-dried. The resulting solid was percolated with methanol to obtain the crude product which was further purified by flash column chromatography, eluent ethyl acetate/hexane 1:1 to get the title compound as a white powder (0.08 g, 4%). ^{1}H NMR (400 MHz, DMSO-d_6) δ 8.17 (s, 1H), 7.92 (s, 2H), 7.17 (s, 1H), 3.76 (s, 3H), 3.17 (s, 3H), 1.72 (ddd, *J* = 28.4, 14.9, 6.2 Hz, 4H), 1.43 (s, 2H), 1.21 (s, 9H). MS (ESI$^+$) m/z: 395.8 ([M + H]$^+$).

Methyl 5-((*tert*-Butoxycarbonyl)(3-methoxypropyl)amino)-2-(3,4-dichloro-5-methyl-1*H*-pyrrole-2-carboxamido)benzo[*d*]thiazole-6-carboxylate (18). A mixture of 3,4-dichloro-5-methyl-1*H*-pyrrole-2-carboxylic acid

(44 mg, 0.23 mmol, 1.2 eq) and $SOCl_2$ (0.75 mL, 2.5 mL/mmol) was stirred at 75 °C for 40 minutes. The volatiles were removed under reduced pressure, then the above 2-amino benzothiazole 2g (75 mg, 0.19 mmol, 1 eq) and toluene (4.5 mL) were added. The resulting mixture was stirred at 130 °C overnight. The precipitate was collected and triturated with toluene and methanol to get the title compound as a black solid (54 mg, 50%). ^{1}H NMR (400 MHz, DMSO-d_6) δ 12.36 (s, 1H), 11.98 (s, 1H), 8.53 (s, 1H), 7.68 (s, 1H), 3.80 (d, *J* = 16.9 Hz, 3H), 3.75 (d, *J* = 4.8 Hz, 1H), 3.53 (s, 1H), 3.17 (s, 3H), 2.28 (s, 3H), 1.79 (d, *J* = 34.7 Hz, 2H), 1.45 (s, 3H), 1.22 (s, 6H).

5-((*tert*-Butoxycarbonyl)(3-methoxypropyl)amino)-2-(3,4-dichloro-5-methyl-1*H*-pyrrole-2-carboxamido)benzo[*d*]thiazole-6-carboxylic Acid (20). To a suspension of 18 (52 mg, 0.09 mmol, 1 eq) in methanol (5 mL) 2 M NaOH (0.23 mL, 0.456 mmol, 5 eq) was added portionwise until the reaction was completed while continuously heating at 60 °C for 48 h. The reaction mixture was neutralised with 1 M HCl. The precipitate was collected and the solid was washed with cold water containing few drops of methanol and ethyl acetate to get the title compound as a brown solid (0.04 g, 83%). ^{1}H NMR (400 MHz, DMSO-d_6) δ 12.88 (s, 1H), 12.40 (s, 1H), 11.97 (s, 1H), 8.51 (s, 1H), 7.64 (s, 1H), 3.77 (s, 2H), 3.17 (s, 3H), 2.28 (s, 3H), 1.83 (s, 2H), 1.45 (s, 3H), 1.24 (s, 6H) (*water or DMSO peak overlaps one signal of CH_2 group). MS (ESI$^-$) m/z: 556.0 ([M – H]$^-$).

2-(3,4-Dichloro-5-methyl-1*H*-pyrrole-2-carboxamido)-5-((3-methoxypropyl)amino)benzo[*d*]thiazole-6-carboxylic Acid (E). A mixture of **20** (40 mg, 0.07 mmol, 1 eq) and 4 M HCl in 1,4-dioxane (0.35 mL) was stirred at 22 °C monitored by LC-MS, then concentrated to obtain title compound as a dark brown solid (17 mg, 51%). ^{1}H NMR (400 MHz, DMSO-d_6) δ 12.37 (s, 1H), 8.34 (s, 1H), 6.85 (s, 1H), 3.50–3.41 (m, 2H), 3.27 (s, 3H), 3.24 (d, *J* = 6.6 Hz, 2H), 2.26 (s, 3H), 1.94–1.79 (m, 2H). ^{13}C NMR (101 MHz, DMSO-d_6) δ 169.45, 150.16, 130.06, 129.64, 129.62, 128.64, 125.87, 109.66, 108.21, 69.73, 58.00, 28.39, 10.97, 0.09. MS (ESI$^+$) m/z: 457.9 ([M + H]$^+$). HRMS (ESI) m/z: [M – H]$^+$ Calcd for $C_{18}H_{19}O_4N_4Cl_2S$ 457.0499; Found 457.0486 (– 2.64 ppm). HPLC purity: 95%.

Determination of Enzyme inhibitory activities

Determination of inhibitory activities on *E. coli* DNA gyrase and topoisomerase IV

Commercially available assay kits from Inspiralis were used for determination of the IC_{50} values for test compounds for inhibition of DNA gyrase supercoiling and Topo IV relaxation. The assay was performed according to previously reported procedures.[1] IC_{50} values were determined with seven concentrations of the inhibitors. GraphPad Prism program was used for calculating IC_{50} value which represents the concentration of inhibitor where the activity of the enzyme is reduced by 50%. IC_{50} values were determined in three independent measurements and their average value is given as a result. As a positive control novobiocin (Sigma-Aldrich) for *E. coli* gyrase was used.

Determination of inhibitory activities on human topoisomerase IIα

Inhibitory activities against topo IIα were determined with commercially available relaxation assay kits (Inspiralis Limited, Norwich, UK) on Pierce streptavidin coated 96-well microtiter plates (Thermo Scientific, Rockford, IL, USA). The plates were rehydrated with wash buffer (20 mM Tris·HCl, 137 mM NaCl, 0.01% w/v BSA, 0.05% v/v Tween 20, pH 7.6) and then biotinylated triplex forming oligonucleotide dissolved in wash buffer added for 5 min to immobilize. The unbound oligonucleotide was washed off with wash buffer. Next, enzymatic reaction was performed: the reaction volume of 30 μL in buffer (50 mM Tris·HCl, 10 mM $MgCl_2$, 125 mM NaCl, 5 mM DTT, 0.1 μg/mL albumin, 1 mM ATP, pH 7.5) contained 0.75 μg of supercoiled pNO1 plasmid, 1.5 U of human DNA topoisomerase II, inhibitor, 1% DMSO and 0.008% Tween 20. Reaction mixtures were incubated at 37 °C for 30 min. After that, the TF buffer (50 mM NaOAc, 50 mM NaCl and 50 mM $MgCl_2$, pH 5.0) was added and the mixtures were left for 30 min at RT, during which biotin–oligonucleotide–plasmid triplex was formed. The unbound plasmid was washed off with TF buffer. Then the solution of Diamond Dye in T10 buffer (10 mM Tris·HCl, 1 mm EDTA, pH 8.0) was added. After 15 min of incubation in the dark, fluorescence was measured with a microplate reader (BioTek Synergy H4, excitation: 485 nm, emission: 537 nm). Initial screening was done at 10 and 100 μM concentrations of inhibitors followed by IC_{50} determination for active compounds, using seven concentrations of tested compounds. GraphPad Prism 6 software was used to calculate the IC_{50} values. The results are reported as the average value of at least two independent measurements. As the positive control, etoposide (TCI, Tokyo, Japan; IC_{50} = 71 μM) was used.

Determination of antibacterial activity

Clinical microbiology strains of *A. baumannii* (ATCC 17978), *E. coli* (ATCC 25922), *Enterobacter cloacae* (ATCC 13047), *Enterococcus faecium* (ATCC 700221), *K. pneumoniae* (ATCC 10031), *P. aeruginosa* (ATCC 27853), *S. aureus* (ATCC 29213), methicillin resistant *S. aureus* (ATCC 43300) and vancomycin-intermediate *S. aureus* (ATCC 700699) were obtained from American Type Culture Collection (ATCC) via Microbiologics Inc. (St. Cloud, MN, USA). The GyrB mutant strains of methicillin- resistant *S. aureus* (ATCC 43300) and vancomycin-intermediate *S. aureus* (ATCC 700699) were generated according to the published protocol.[2] Antimicrobial assays (Minimum inhibitory concentrations (MICs)) were performed by standard serial broth microdilution method, following the Clinical and Laboratory Standards Institute guidelines.[3]

X-ray Crystallography

Protein expression and purification: E. coli GyrB24 protein, purified as described previously,[4] was concentrated to approximately 11 mg/mL in 50 mM Tris×HCl pH 7.9, 50 mM NaCl, 5 mM DTT. This construct corresponds to residues 1-220 of the full-length wild-type protein (UniProtKB entry P0AES6), with a calculated molecular weight of 24,157 Da, and is referred to as EcGyrB24.

Crystallization, X-ray data collection and structure solution: Crystals were grown using the sitting drop vapour diffusion method from the sample above in the presence of 1 mM **A**. Commercially available (Molecular Dimensions, Qiagen) and in-house crystallization screens were set up in MRC2 96-well crystallization plates (Swissci) with drops comprised of 0.3 μL precipitant and 0.3 μL of protein solution using an Oryx 8 liquid handling robot (Douglas Instruments) and then equilibrated against 50 μL of reservoir solution at a constant temperature of 19 °C. Crystals were mounted in Litholoops (Molecular Dimensions) before flash-cooling by plunging into liquid nitrogen prior to transport to the synchrotron. X-ray data were recorded on beamline I03 at the Diamond Light Source (Oxfordshire, UK) using a Pilatus 6M hybrid photon counting detector (Dectris), with crystals maintained at 100 K by a Cryojet cryocooler (Oxford Instruments).

X-ray data were integrated and scaled using XDS[5] via the XIA2 expert system[6] and merged using AIMLESS[7] (data statistics are shown in Table S1). All successive data processing was carried out using programs in the CCP4 suite via the CCP4i2 graphical user interface.[8] The structure was solved via molecular replacement in PHASER,[9] and the resultant model was finalised by successive iterations of model building in COOT,[10] and restrained refinement in REFMAC5[11] until no further improvements could be achieved.

Starting coordinates and restraints for the ligand were generated using AceDRG[12] before docking these into suitable electron density. The final model was validated using MOLPROBITY[13] and the PDB-validation server (https://validate-rcsb-2.wwpdb.org). Refinement and validation statistics are summarized in Table S1.

Crystals of the EcGyrB24-**A** complex were obtained using a precipitant comprised of 34% (w/v) PEG 4000, 95 mM $MgCl_2$, 100 mM Tris×HCl pH 8.0, and were cryo-cooled directly from the crystallization drop. Data were recorded to 1.16 Å resolution in space group *C*2. The structure was solved by molecular replacement using a non-isomorphous structure of EcGyrB24 (PDB accession code 1KZN) giving a single copy of the protein chain in the asymmetric unit with an estimated solvent content of 40%.

Molecular modelling

Molecular docking

Molecular docking calculations were performed in Schrödinger Release 2022-1 (Schrödinger, LLC, New York, NY, USA, 2022). Crystal structure of *E. coli* GyrB (PDB ID 4WUB) was retrieved from Protein Data Bank. Protein was then prepared by Protein Preparation Wizard using default settings. Receptor grid was calculated for the ligand-binding site and compounds **D** and **E** were docked using Glide XP protocol as implemented in Schrödinger Release 2022-1 (Glide, Schrödinger, LLC, New York, NY, USA, 2022). The highest ranked docking pose was used for visualization.

Molecular dynamic simulations

MD simulation of compound **D** or compound **E** in complex with *E. coli* GyrB was performed using NAMD package (version 2.9)[14] and the CHARMM36m[15] force field. Removal of potential steric clashes and optimization of the atomic coordinates of the docking complex were first performed by steepest descent (10.000 steps) and adopted basis Newton-Raphson (10.000 steps) energy minimizations. The system for MD simulation was prepared using CHARMM-GUI.[16,17] Structure of the compound **D**-GyrB or compound **E**-GyrB complex was first embedded in a box of TIP3P water molecules. Then the system was neutralized by addition of KCl. The MD simulation was run in the NPT ensemble using the periodic boundary conditions. Temperature (300 K) and pressure (1 atm) were controlled using the Langevin dynamics and Langevin piston methods, respectively. Short-range and long-range forces were calculated every 1 and 2 time steps, respectively, with a time step of 2.0 ps. The smooth particle mesh Ewald method was used to calculate the electrostatic interactions.[18] The short-range interactions were cut off at 12 Å. All of the chemical bonds between hydrogen and the heavy atoms were held fixed using the SHALE algorithm.[19] After equilibration of the complete system for 1 ns, an unconstrained 100 ns production was run.

Structure-based pharmacophore modeling

The 100 nm MD trajectory of *E. coli* GyrB in complex with compound **D** or compound **E** was used for pharmacophore feature analysis using LigandScout 4.4 Expert,[20] which resulted in 1000 structure-based pharmacophore models.

References

(1) Durcik, M.; Tammela, P.; Barančoková, M.; Tomašič, T.; Ilaš, J.; Kikelj, D.; Zidar, N. Synthesis and Evaluation of N-Phenylpyrrolamides as DNA Gyrase B Inhibitors. *ChemMedChem* **2018**, *13* (2), 186–198.

(2) Nyerges, A.; Tomašič, T.; Durcik, M.; Revesz, T.; Szili, P.; Draskovits, G.; Bogar, F.; Skok, Ž.; Zidar, N.; Ilaš, J.; Zega, A.; Kikelj, D.; Daruka, L.; Kintses, B.; Vasarhelyi, B.; Foldesi, I.; Kata, D.; Welin, M.; Kimbung, R.; Focht, D.; Mašič, L. P.; Pal, C. Rational Design of Balanced Dual-Targeting Antibiotics with Limited Resistance. *PLoS Biol.* **2020**, *18* (10), No. e3000819.

(3) Carpenter, D. E. Methods for Antimicrobial Susceptibility Testing of Anaerobic Bacteria. 10.

(4) Lewis, R. J.; Singh, O. M.; Smith, C. V.; Maxwell, A.; Skarzynski, T.; Wonacott, A. J.; Wigley, D. B. Crystallization of Inhibitor Complexes of an N-Terminal 24 KDa Fragment of the DNA Gyrase B Protein. *J. Mol. Biol.* **1994**, *241* (1), 128–130.

(5) Kabsch, W. XDS. *Acta Crystallogr. D Biol. Crystallogr.* **2010**, *66*, 125–132.

(6) Winter, G. Xia2: An Expert System for Macromolecular Crystallography Data Reduction. *J. Appl. Cryst.* **2010**, *43* (1), 186–190.

(7) Evans, P. R.; Murshudov, G. N. How Good Are My Data and What Is the Resolution? *Acta Crystallogr. D Biol. Crystallogr.* **2013**, *69*, 1204–1214.

(8) Potterton, L.; Agirre, J.; Ballard, C.; Cowtan, K.; Dodson, E.; Evans, P. R.; Jenkins, H. T.; Keegan, R.; Krissinel, E.; Stevenson, K.; Lebedev, A.; McNicholas, S. J.; Nicholls, R. A.; Noble, M.; Pannu, N. S.; Roth, C.; Sheldrick, G.; Skubak, P.; Turkenburg, J.; Uski, V.; von Delft, F.; Waterman, D.; Wilson, K.; Winn, M.; Wojdyr, M. CCP4i2: The New Graphical User Interface to the CCP4 Program Suite. *Acta Crystallogr. D Struct. Biol.* **2018**, *74*, 68–84.

(9) McCoy, A. J.; Grosse-Kunstleve, R. W.; Adams, P. D.; Winn, M. D.; Storoni, L. C.; Read, R. J. Phaser Crystallographic Software. *J. Appl. Crystallogr.* **2007**, *40*, 658–674.

(10) Emsley, P.; Cowtan, K. Coot: Model-Building Tools for Molecular Graphics. *Acta Crystallogr. D Biol. Crystallogr.* **2004**, *60*, 2126–2132.

(11) Murshudov, G. N.; Skubák, P.; Lebedev, A. A.; Pannu, N. S.; Steiner, R. A.; Nicholls, R. A.; Winn, M. D.; Long, F.; Vagin, A. A. REFMAC5 for the Refinement of Macromolecular Crystal Structures. *Acta Crystallogr. D Biol. Crystallogr.* **2011**, *67*, 355–367.

(12) Long, F.; Nicholls, R. A.; Emsley, P.; Gražulis, S.; Merkys, A.; Vaitkus, A.; Murshudov, G. N. *AceDRG* : A Stereochemical Description Generator for Ligands. *Acta Crystallogr. D Struct. Biol.* **2017**, *73* (2), 112–122.

(13) Davis, I. W.; Leaver-Fay, A.; Chen, V. B.; Block, J. N.; Kapral, G. J.; Wang, X.; Murray, L. W.; Arendall, W. B.; Snoeyink, J.; Richardson, J. S.; Richardson, D. C. MolProbity: All-Atom Contacts and Structure Validation for Proteins and Nucleic Acids. *Nucleic Acids Res.* **2007**, *35*, W375-383.

(14) Phillips, J. C.; Braun, R.; Wang, W.; Gumbart, J.; Tajkhorshid, E.; Villa, E.; Chipot, C.; Skeel, R. D.; Kalé, L.; Schulten, K. Scalable Molecular Dynamics with NAMD. *J. Comput. Chem.* **2005**, *26* (16), 1781–1802.

(15) Huang, J.; Rauscher, S.; Nawrocki, G.; Ran, T.; Feig, M.; de Groot, B. L.; Grubmüller, H.; MacKerell, A. D. CHARMM36m: An Improved Force Field for Folded and Intrinsically Disordered Proteins. *Nat. Methods* **2017**, *14* (1), 71–73.

(16) Jo, S.; Kim, T.; Iyer, V. G.; Im, W. CHARMM-GUI: A Web-Based Graphical User Interface for CHARMM. *J. Comput. Chem.* **2008**, *29* (11), 1859–1865.

(17) Lee, J.; Cheng, X.; Swails, J. M.; Yeom, M. S.; Eastman, P. K.; Lemkul, J. A.; Wei, S.; Buckner, J.; Jeong, J. C.; Qi, Y.; Jo, S.; Pande, V. S.; Case, D. A.; Brooks, C. L.; MacKerell, A. D.; Klauda, J. B.; Im, W. CHARMM-GUI Input Generator for NAMD, GROMACS, AMBER, OpenMM, and CHARMM/OpenMM Simulations Using the CHARMM36 Additive Force Field. *J. Chem. Theory Comput.* **2016**, *12* (1), 405–413.

(18) Essmann, U.; Perera, L.; Berkowitz, M. L.; Darden, T.; Lee, H.; Pedersen, L. G. A Smooth Particle Mesh Ewald Method. *J. Chem. Phys.* **1995**, *103* (19), 8577–8593.

(19) Ryckaert, J.-P.; Ciccotti, G.; Berendsen, H. J. C. Numerical Integration of the Cartesian Equations of Motion of a System with Constraints: Molecular Dynamics of n-Alkanes. *J. Comput. Phys.* **1977**, *23* (3), 327–341.

(20) Wolber, G.; Langer, T. LigandScout: 3-D Pharmacophores Derived from Protein-Bound Ligands and Their Use as Virtual Screening Filters. *J. Chem. Inf. Model* **2005**, *45* (1), 160–169.
